# Successive Ring Expansion Strategies for the Synthesis of Macrocycles and Medium-Sized Rings

**DOI:** 10.1021/acs.chemrev.6c00360

**Published:** 2026-07-31

**Authors:** Jonathan P. Knowles, William P. Unsworth

**Affiliations:** † School of Geography and Natural Sciences, 5995 Northumbria University, Ellison Place, Newcastle upon Tyne NE1 8ST, U.K.; ‡ Department of Chemistry, 8748 University of York, Heslington, York YO10 5DD, U.K.

## Abstract

Macrocycles and medium-sized rings have myriad uses in various applied fields. Effective and scalable methods for their synthesis are therefore of high value. Ring expansion reactions are important in this context, as they allow larger rings to be “grown” from more easily accessible smaller ring systems, while avoiding inefficient end-to-end cyclization reactions. This review is focused on successive ring expansion strategies, in which two (or more) ring expansion steps have been performed in sequence to enable the synthesis of functionalized macrocycles and medium-sized rings.

## Graphical abstract

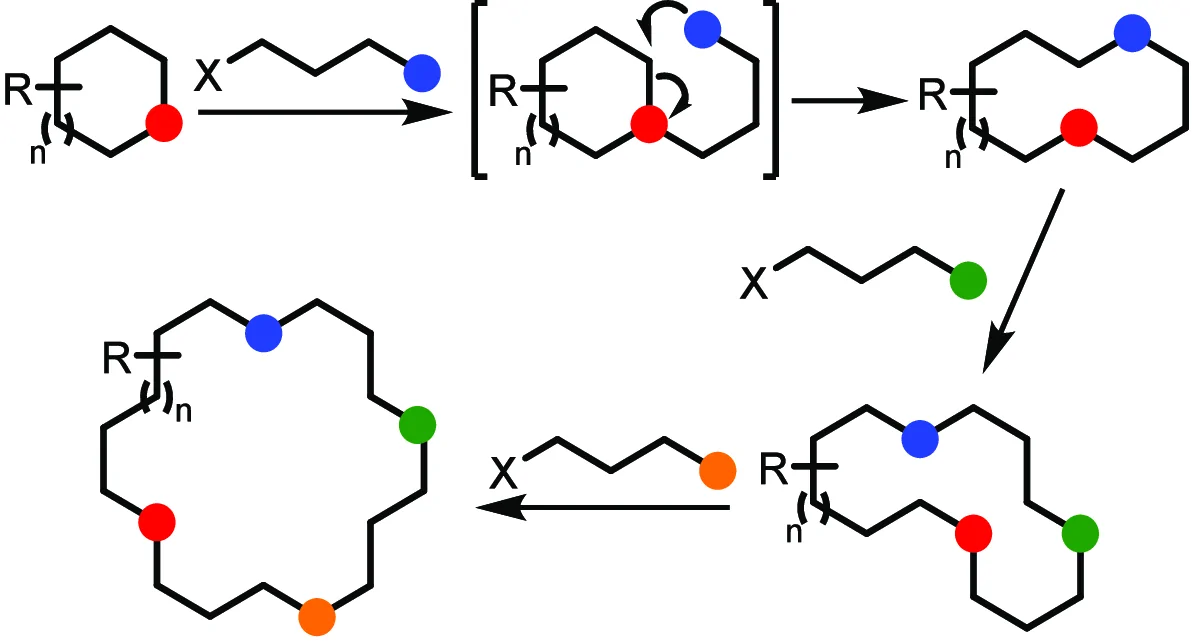

## 1 Introduction

Ring expansion strategies have a celebrated history in synthetic organic chemistry,^1–8^ with the “named” ring expansion reactions developed in the groups of Grob^9–11^ and Eschenmoser^12^ probably the most well-known. These important works stand alongside vital contributions from the groups of Hesse,^13,14^ Cookson,^15^ and many others.^16–26^ Ring expansion strategies enable cyclic molecules to be formed through controlled rearrangement reactions, with fundamentally altered scaffolds compared to their precursors. Such transformations are especially useful if they allow access to ring systems that are difficult to prepare via standard methods, notable examples being macrocycles (≥12-membered rings) and medium-sized rings (8- to 11-membered rings). Both classes are difficult to synthesize via direct end-to-end cyclization reactions (e.g., **1** → **2**, Scheme 1) compared to smaller ring sizes, with intermolecular side reactions (e.g., dimerization, **1** → **3**) often outcompeting the desired cyclization; this is due to a combination of loss of entropy upon cyclization, conformational effects and transannular strain (especially pertinent for medium-sized rings).^5,7,8,27,28^

**Scheme 1:**
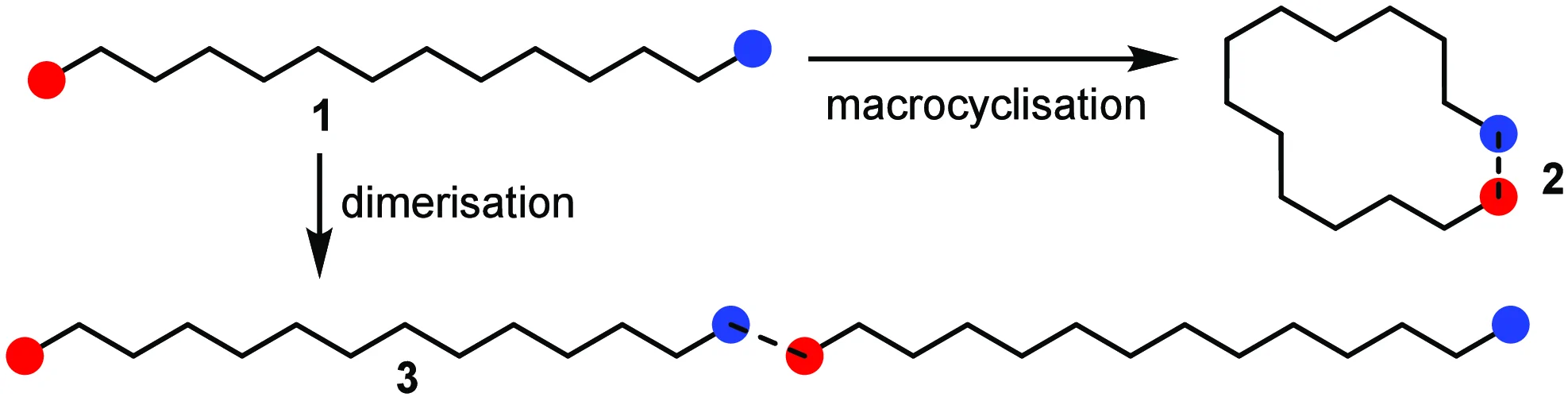
Macrocyclization versus Dimerization

Various strategies have been developed to help improve the efficiency of macrocyclization,^29–32^ the most common being to perform macrocyclization reactions at high dilution to minimize intermolecular side reactions. High-dilution methods can be effective; however, they tend to compromise the practicality of the synthesis, especially on scale. Macrocycles and medium-sized rings have myriad useful applications, spanning use as ligands,^33–35^ sensors,^36,37^ self-assembly/supramolecular applications,^38–42^ and in medicinal chemistry.^43–50^ Therefore, there is high demand for effective, scalable methods to synthesize diversely functionalized large ring molecules.

Ring expansion methods are important in this context, as they allow the inefficient end-to-end cyclization step that can negatively impact large ring syntheses to be completely bypassed.^1–8^ Ring expansion reactions that can be performed in sequence can be even more powerful, by enabling macrocycles and medium-sized rings to be “grown” from more easily accessible smaller rings systems. This idea is summarized in the general reaction scheme in Scheme 2; breaking down the conversion of **4** into **9** into three efficient four-atom ring expansion reactions, each proceeding via “fast” six-membered ring cyclization steps, can be more efficient than the corresponding “slow” end-to-end macrocyclization of a long linear precursor **10**.

**Scheme 2:**
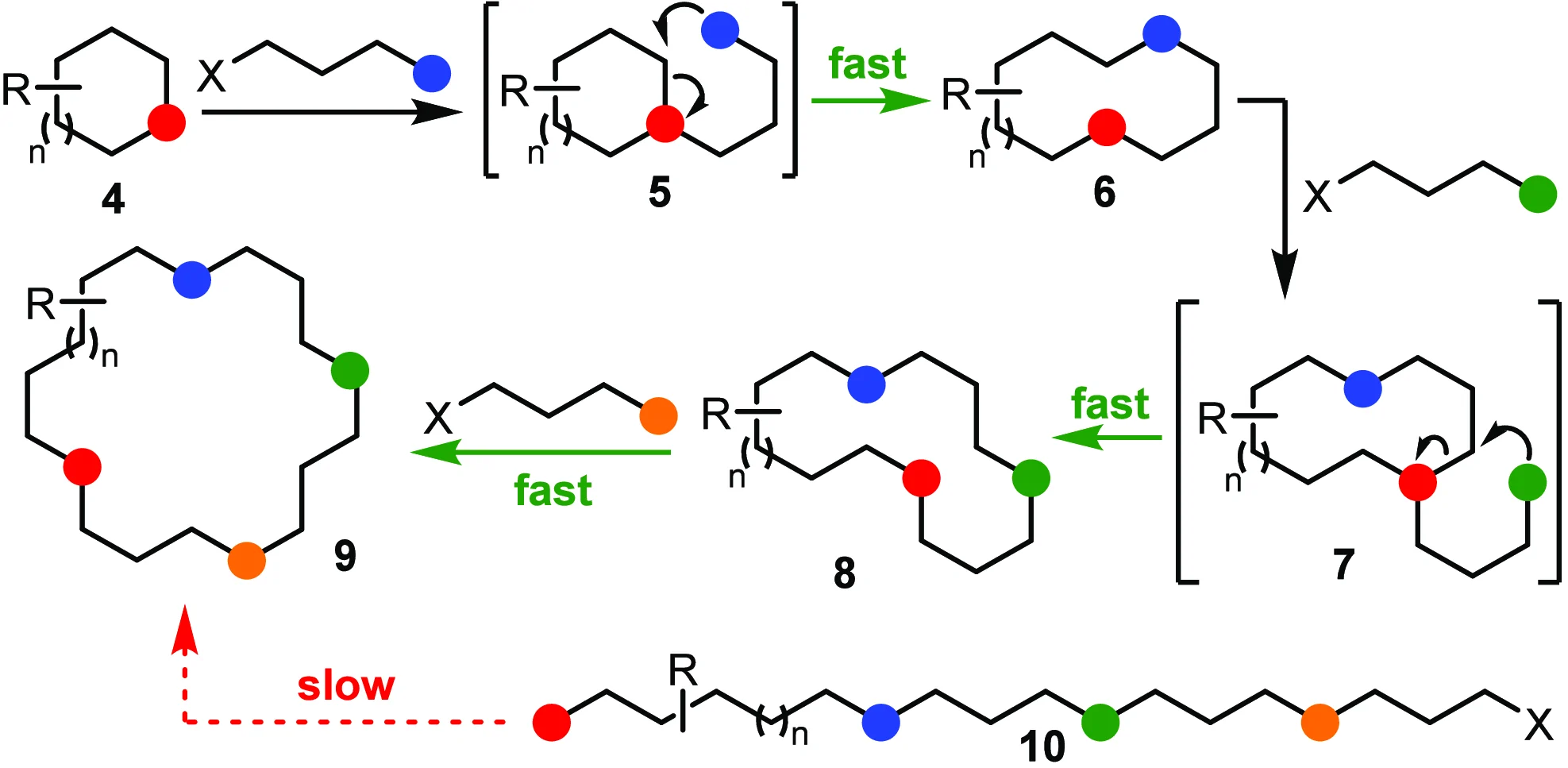
Ring Expansion versus End-to-End Macrocyclization

### 1.1 Scope and Organization

This review focuses on successive ring expansion strategies being used for the synthesis of macrocycles and medium-sized rings. When selecting material for inclusion in the review, two key criteria have been applied:

(1) The final products formed must be either macrocycles or medium-sized rings (i.e., ≥8-membered rings).

(2) The products must have been formed via two or more controlled, sequential ring expansion steps, via either a cascade process or separate synthetic steps.

The intention was that this review would include discussion of all published synthetic strategies that meet both of these criteria. Almost inevitably, we will fall short of this goal, and we apologize in advance to the authors of any studies that are inadvertently omitted. For example, we fully anticipate that there will be published synthetic sequences in which two or more common homologation methods (e.g., Beckmann-, Baeyer–Villiger-, and/or Tiffeneau–Demjanov-type reactions)^51–54^ are used to effect one-atom ring expansion that are missed. Locating such examples is particularly challenging when the ring expansion steps are separated by multiple other synthetic steps within longer synthetic sequences, especially if the advancement of ring expansion methodologies is not the primary goal of the study (for example, in total synthesis).

The review is split into three sections, with the material arranged chronologically within each section. Section 2 features cascade ring expansion reactions. This refers to strategies in which a starting material is set up to undergo two or more ring expansions in sequence, via a controlled cascade process. Section 3 focuses on iterative ring expansion reactions. This involves strategies whereby the same ring expansion or conceptually similar ones are performed iteratively via separate synthetic steps, thus allowing controlled, stepwise ring enlargement. In principle, the iterative strategies in section 3 can all be repeated indefinitely. Section 4 features consecutive ring expansion reactions. As in section 3, this section contains strategies in which ring expansion reactions are performed separately and in sequence to enable controlled stepwise ring enlargement. However, this section is focused on strategies in which fundamentally different ring expansion methods are applied sequentially, in most cases using different ring expansion reactions for each step, thus increasing the range of functional groups that can be introduced into the ring-enlarged products.

The review is focused on fully controlled ring expansion approaches; therefore, despite recent notable efforts to control the extent of this process through the use of host–guest chemistry,^55^ methods for the synthesis of cyclic polymers via ring expansion polymerization are not included.^56–60^ Double ring expansion methods with no clear sequence, for example, the double ring expansion of diketopiperazine derivatives (e.g., **11** → **12**, Scheme 3A)^61–64^ and the Rh-catalyzed double Stevens rearrangement of bis­(thioethers) (e.g., **13** → **14**, Scheme 3B), are also not covered.^65^

**Scheme 3:**
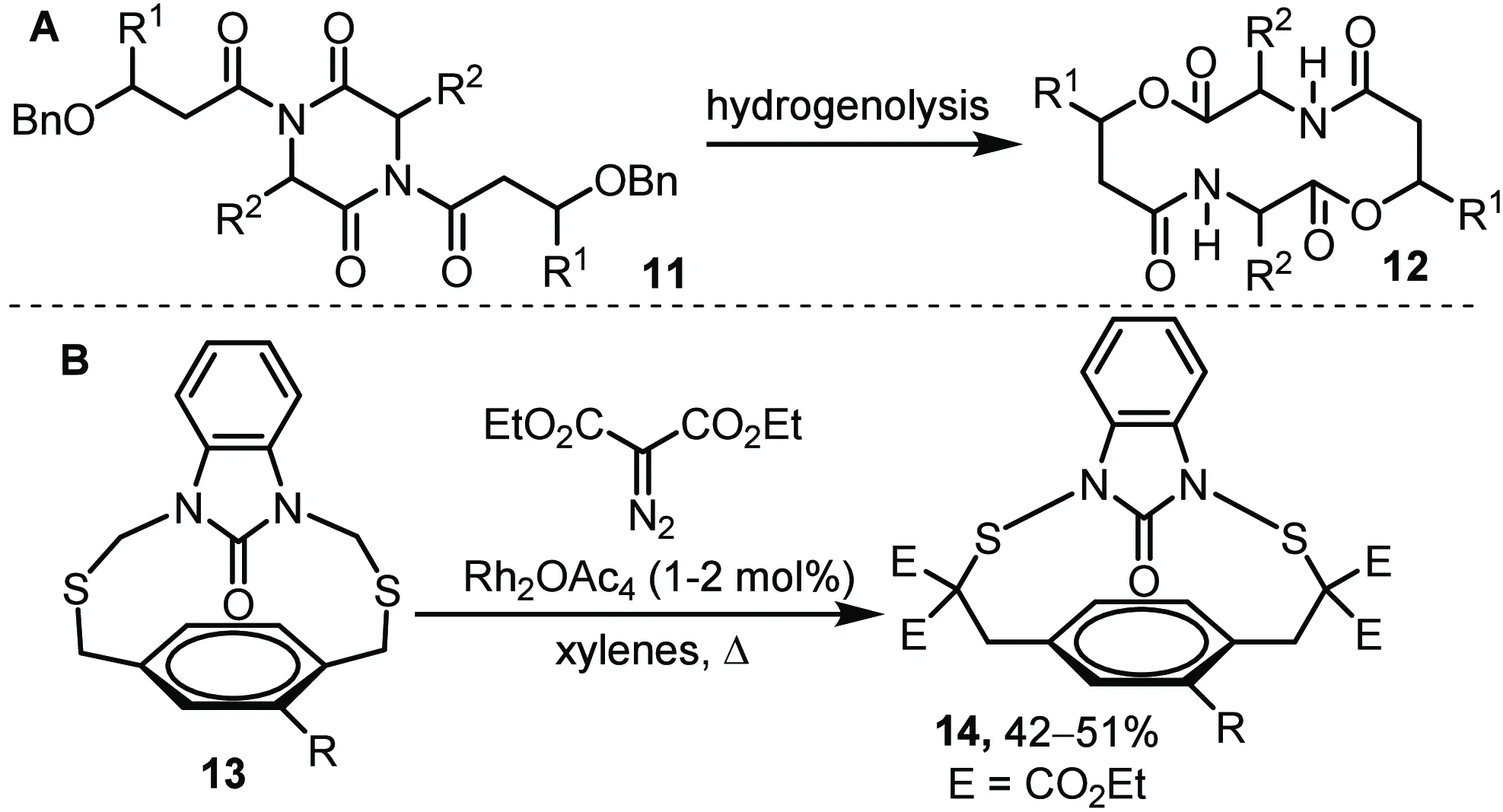
Double Ring Expansion of (A) Diketopiperazine Derivatives and (B) Bis­(thioethers)

## 2 Cascade Ring Expansion Reactions

This section is focused on strategies in which a starting material is set up to undergo two or more ring expansions in a single cascade process, leading to the formation of a medium-sized ring or macrocyclic product. Methods starting from both cyclic and linear starting materials are included.

### 2.1 Transesterification Cascades

A seminal study by Corey and co-workers^66^ showed that successive cascade ring expansion can be achieved via reversible transesterification reactions that operate under thermodynamic control. Thus, 15-membered lactone **17** was produced via two intramolecular transesterifications (Scheme 4). In this work, nine-membered-ring lactone precursor **15** was first reacted with TBAF in THF to form dihydroxylactone **16a**. Then, ring expansion was then promoted using catalytic *p*-TSA to produce 15-membered lactone **17** in an impressive 90% yield. As each ring expansion step results in no overall change in the types of bond in the product (i.e., lactone → lactone), the efficiency of this rearrangement likely reflects the relief of strain upon rearranging a medium-sized ring to form an isomeric 15-membered-ring macrocycle.^5,27,28^

**Scheme 4:**
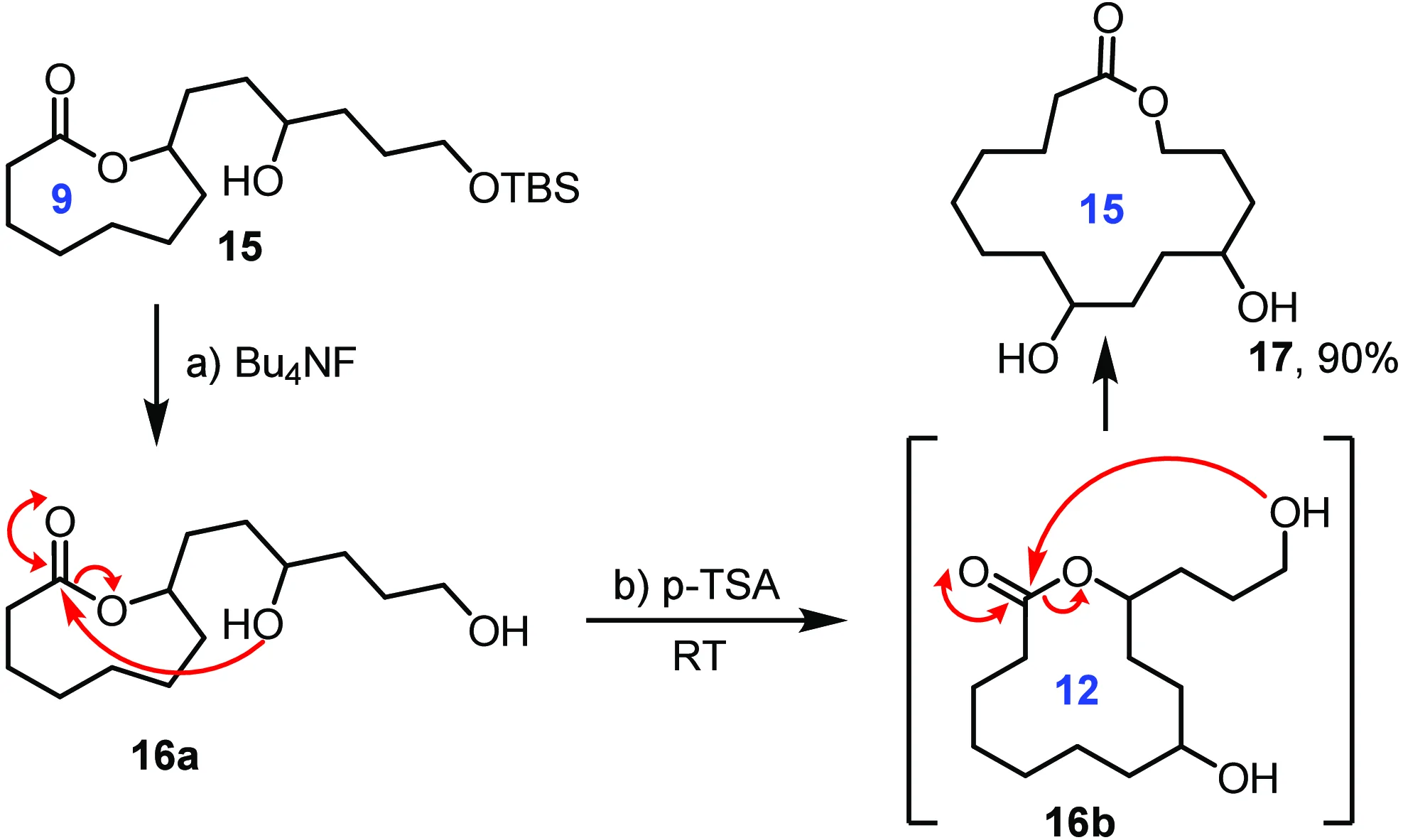
Transesterification Cascade to Lactone **17**

### 2.2 Transamidation Cascades and Zip Reactions

Hesse and co-workers pioneered a series of cascade ring expansions known as “zip reactions”, named “in analogy with the behavior of a zip fastener”.^67–69^ Zip reactions enable polyamine macrocycles to be assembled via successive transamination reactions, as illustrated in Scheme 5A.^67^ The reaction of sodiated laurolactam **18** with acrylonitrile, reduction to form amine **19**, and a second round of alkylation and reduction afforded diamine **20a**. Treatment of **20a** under basic conditions at reflux then set up a cascade rearrangement, resulting in two sequential ring expansion steps (**20a** → **20b** → **20c**). The formation of a resonance-stabilized anion (i.e., the amide anion of **21**) is thought to provide the driving force for this rearrangement. The same strategy can also be used to generate large macrocycles via longer cascades; for example, 33-membered polyamine lactam **22** was synthesized in high yield using the same approach—this time via five sequential ring expansion reactions.^68^ An even more striking example was the Hesse group’s synthesis of 54-membered macrocycle **24** in 38% yield from 13-membered lactam polyamine derivative **23** (Scheme 5B).^69^

**Scheme 5:**
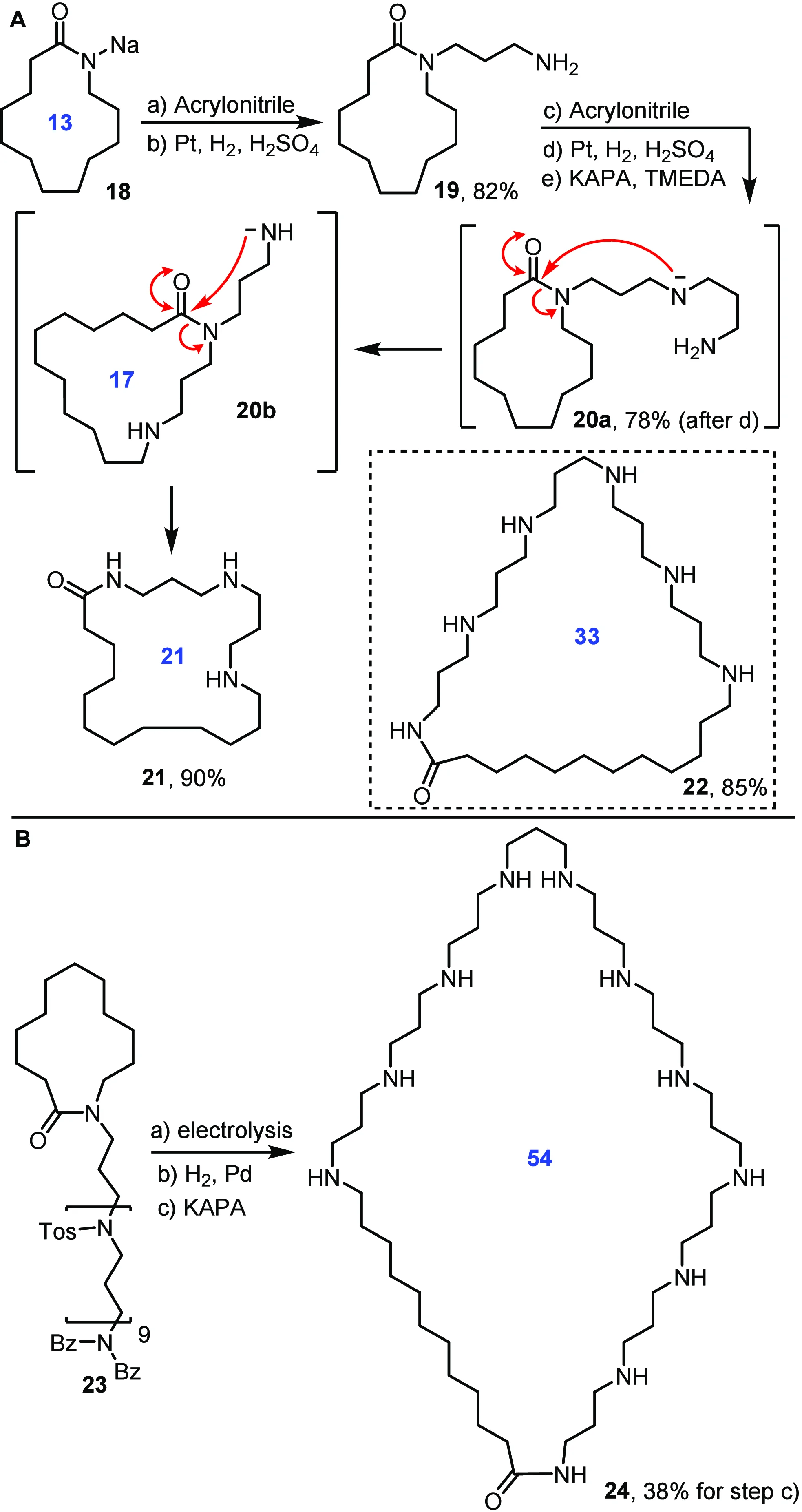
Zip Reactions to Generate Polyamine Macrocycles **22** and **24**

Ring expansion of normal-sized rings (five- to seven-membered rings) to form medium-sized rings can often be particularly challenging, in comparison to other ring systems,^5^ especially if the reactions are under thermodynamic control. Medium-sized rings often suffer from destabilizing transannular interactions and other strain compared with normal-sized rings, thus providing an increased barrier to their formation.^5^ In some cases, the problems associated with the instability of medium-sized ring products can be overcome by performing consecutive ring expansion reactions in one-pot. For example, an instructive example concerns the cascade ring expansion of six-membered-ring barbiturate **25a** into 14-membered macrocycle **26** (Scheme 6). In this case, the intermediate 10-membered ring **25b** is unstable with respect to ring contraction back to its six-membered precursor **25a**. However, the second ring expansion in the cascade means that an equilibrium is established in which the more stable 14-membered-ring product **26** is formed; in this case, the second ring expansion benefits from release of strain, upon converting a medium-sized ring into a macrocycle.^70^

**Scheme 6:**
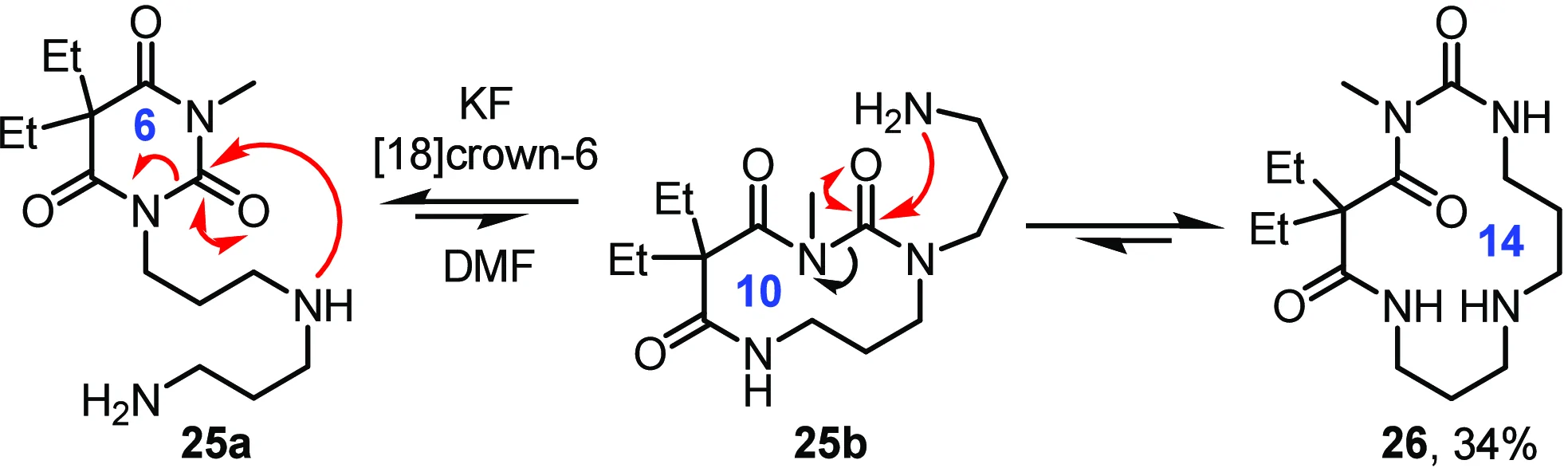
Reversible 6- to 10- to 14-Membered-Ring Cascade Ring Expansion

Takahashi and co-workers published a similar approach during their synthesis polyamine alkaloid-like compounds, designed for use as Fe­(II) chelators in water.^71^ Thus, coumarin (**27**) was reacted with tetramine **28** in methanol at reflux, and after a long reaction time, macrocycle **29d** was formed in 20% yield (Scheme 7). The reaction is thought to operate via an initial conjugate addition, followed by an intramolecular amidation and one atom ring expansion (**27** → **29a** → **29b**), followed by sequential transamidation reactions (**29b** → **29c** → **29d**). The sequence is presumably under thermodynamic control, with relief of the strain associated with the medium-sized ring **29c** providing a driving force for the final ring expansion to generate macrocycle **29d**.

**Scheme 7:**
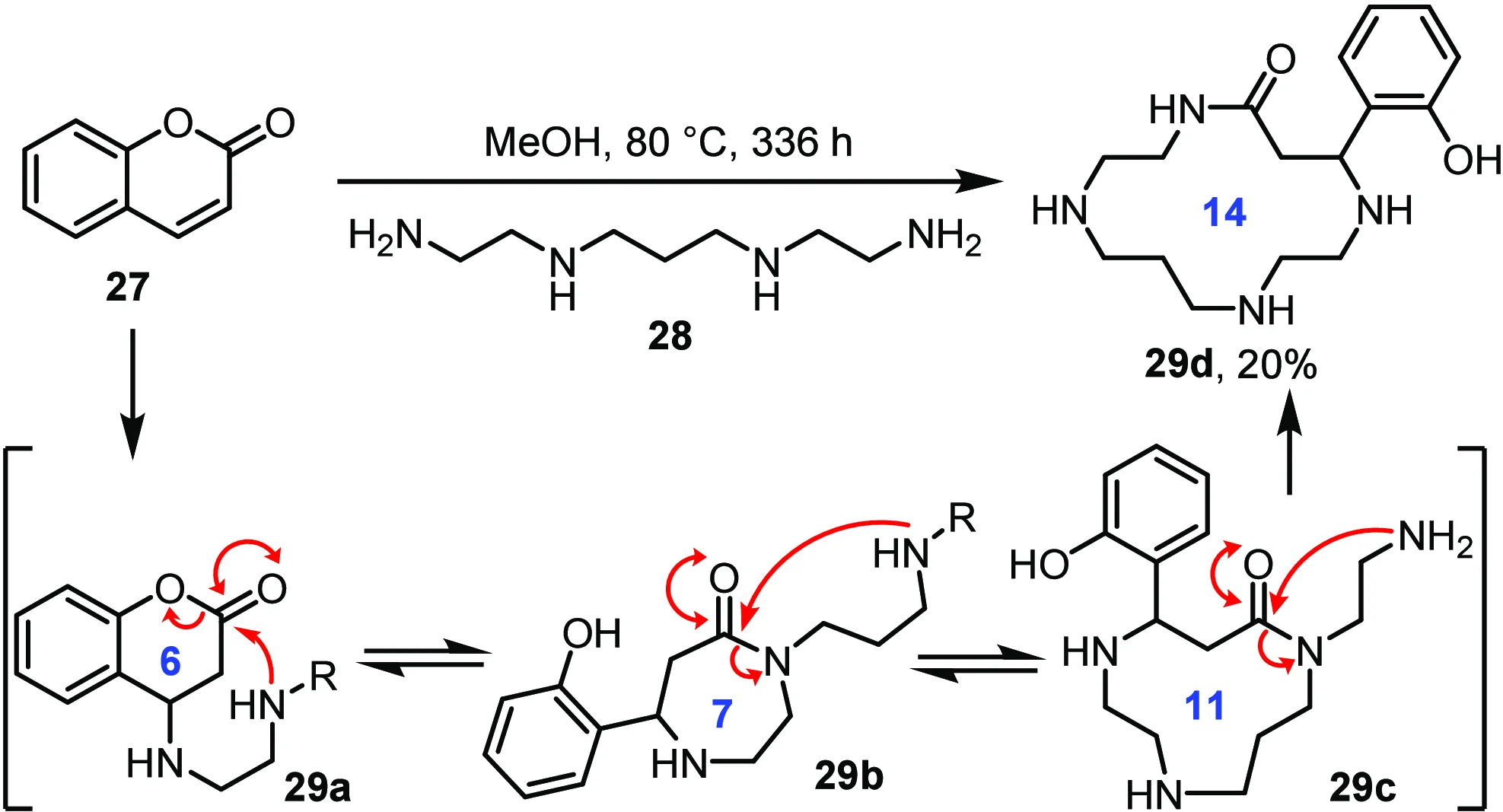
Cascade Ring Expansion Reactions for the Conversion of Coumarin (**27**) into 14-Membered Lactam **29d**

Tam and co-workers^72,73^ reported an innovative “thia-zip reaction” for the synthesis of cysteine-rich macrocyclic peptides, inspired by Hesse and co-workers’ aza-zip reaction summarized in Scheme 5 above. Tam’s strategy relies on reversible thiolactone exchange reactions and has been demonstrated to provide a significant cyclization rate enhancement in the formation of >90-membered-ring macrocycles. The general strategy is summarized in Scheme 8. Thus, starting from a cysteine-rich linear peptide, an initial thiolactone ester formation (to form an intermediate of the form **31**) promotes a series of reversible thiolactone exchange reactions, which operate in aqueous conditions at pH 7.5. It is proposed that the formation of macrocycle **32** is driven by a final irreversible S-to-N acyl migration (**31d** → **32**) using an N-terminal cysteine residue, with this amide-forming step providing a thermodynamic driving force for the overall cascade.

**Scheme 8:**
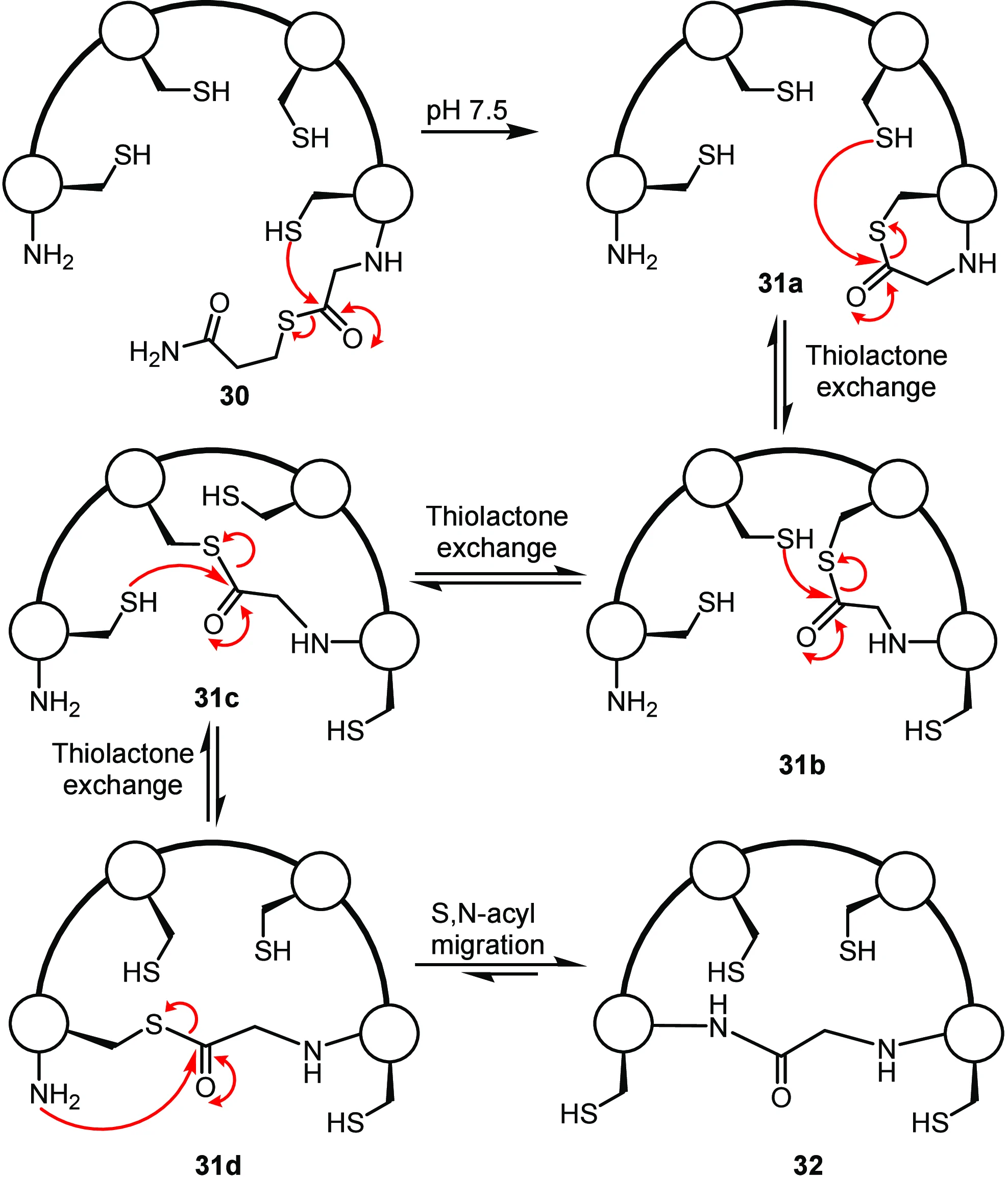
“Thia-Zip” Cascade Ring Expansion

### 2.3 Internal Nucleophilic Catalyst-Mediated Cascades

A cascade ring expansion method for the synthesis of medium-sized-ring lactones and lactams developed by Unsworth and co-workers is summarized in Scheme 9A.^74–76^ In this work linear alcohols (**33**, X = O) or amines (**33**, X = NR/NH) are converted into lactones or lactams (**35**) respectively, by cyclization onto an activated carboxylic acid. As discussed above, direct cyclization reactions to form medium-sized rings are often inefficient due to competing intermolecular reactions. A cascade ring expansion cascade approach was therefore designed that avoids direct end-to-end cyclization and operates exclusively through normal-sized ring (five- to seven-membered) cyclization reactions.

**Scheme 9:**
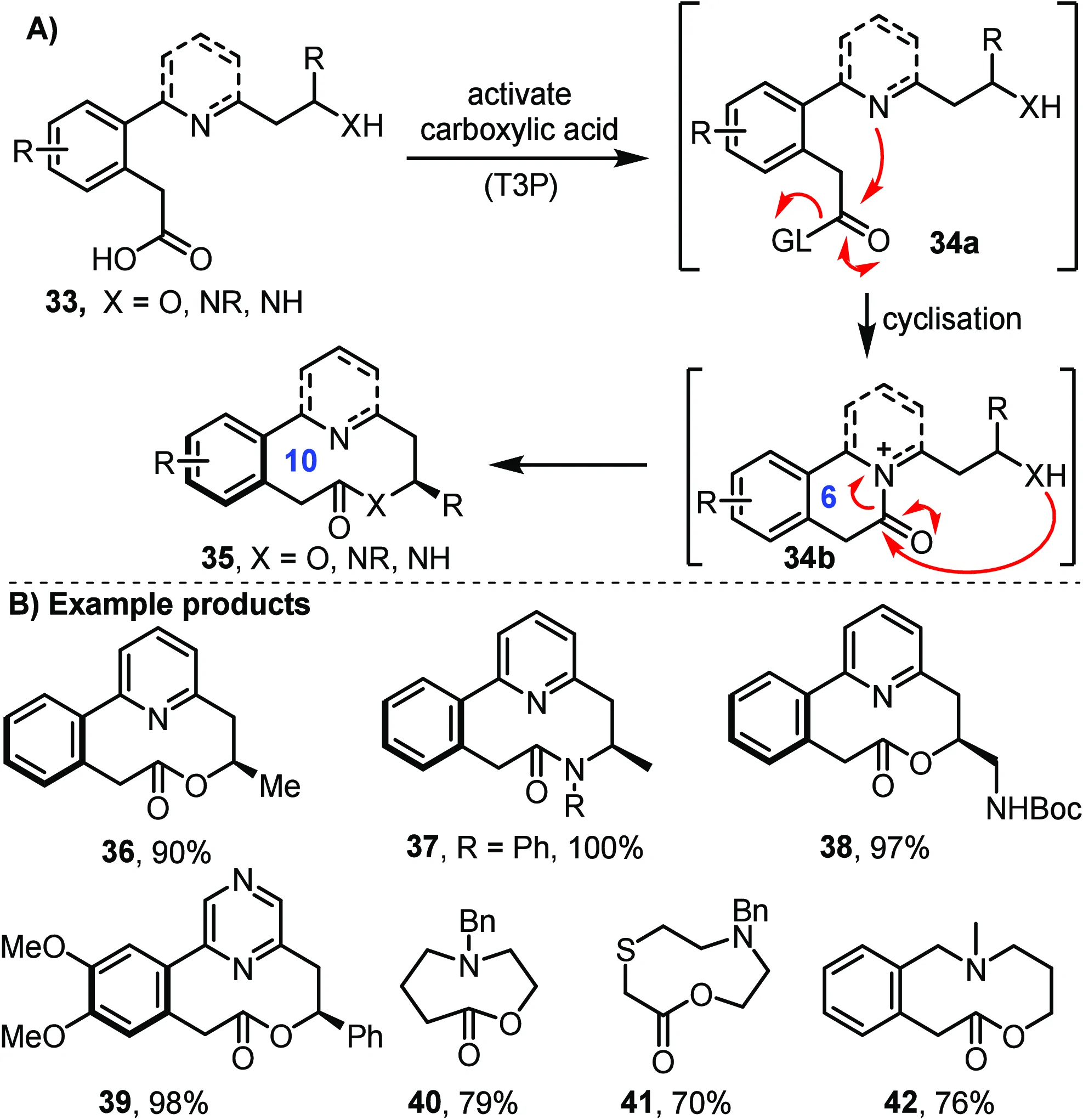
Internal Nucleophilic Catalyst-Mediated Cyclization/Ring Expansion Cascade Reactions

This is achieved via the placement of pyridine/tertiary amine groups in the linear starting material that can act as internal nucleophilic catalysts. Thus, a linear carboxylic acid of the form **33** can be activated (e.g., with T3P) to form an activated derivative **34a** primed to react with the internal pyridine/amine group to form a six-membered-ring acyl ammonium intermediate **34b**. This positively charged intermediate can then undergo ring expansion *in situ*, following nucleophilic attack from a tethered alcohol or amine group to form **35**. The ring expansion step is thought to be driven by neutralization of the positive charge in reactive intermediate **34b**. Various medium-sized-ring lactones and lactams have been prepared using this method (e.g., **36**–**42**, Scheme 9B). The reactions are performed at RT, are insensitive to air and moisture and do not require high dilution to proceed efficiently, with ∼0.1 M reaction concentration typically used in this ring expansion approach. When forming biaryl products (**36**–**39**), formation via a cascade brings an additional benefit of enabling a single atropisomer to be formed in the reaction, with the point stereogenic center imparting complete facial selectivity during the acyl transfer step.^74,77,78^

The cyclization/ring expansion (CRE) concept summarized in Scheme 9 was subsequently extended to encompass a wider range of reactions, with nine distinct CRE reaction modes now demonstrated.^79^ Most are based on the reaction of linear precursors with two terminal nucleophiles with bis-electrophiles (Scheme 10A). For example, diamino alcohol **43** can be converted into thiocarbamate **45** in 83% yield upon reaction with thiophosgene at RT. This reaction is proposed to proceed via an initial cyclization to form five-membered-ring cationic intermediate **43a**, followed by *in situ* ring expansion to nine-membered-ring product **45**. Operating via a similar pathway, diol **47** can be converted smoothly into cyclic sulfite **49** upon reaction with thionyl chloride *via* cationic intermediate **47a**. Diamine **51** can also be converted into cyclic urea **53** *via* cationic intermediate **51a**, via a reaction based on CO_2_ trapping and activation *in situ*. For all of these CRE modes, control reactions were performed using substrates lacking the internal nucleophile (**44**, **48**, **52**), with these results strongly supporting operation via cyclization and subsequent ring expansion as proposed. The syntheses of ∼100 diversely functionalized medium-sized rings have been reported using CRE approaches to date, with selected examples (**55a**–**h**) shown in Scheme 10B.^76,77,79^

**Scheme 10:**
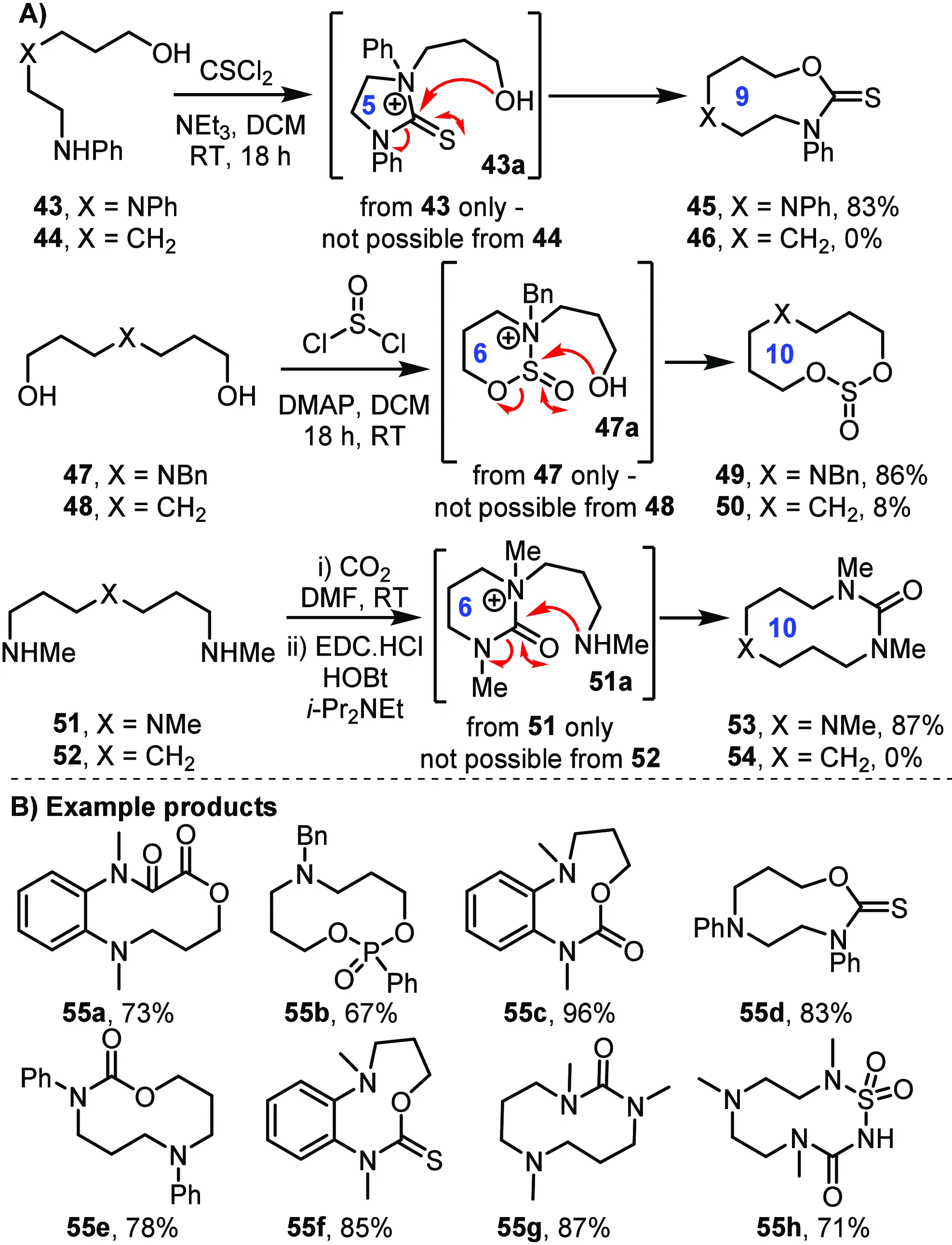
Internal Nucleophilic Catalyst-Mediated Cyclization/Ring Expansion Cascades: (A) Reaction Modes and (B) Example Products

Extended cascades based on the method described in Schemes 9 and 10 have also been developed to allow the synthesis of macrocyclic products.^79^ This is done via the incorporation of more than one internal nucleophile in the linear starting material; this enables longer cascade sequences while still maintaining the key design principle that the reactions operate solely via normal-sized-ring cyclization steps (Scheme 11). This idea is exemplified by the reaction of linear starting material **60**: activation of the carboxylic acid initiates cyclization (**60** → **61a**), followed by two successive ring expansion steps (**61a** → **61b** → **62**) to afford 14-membered-ring lactone **62** in 73% yield, formed as a single atropisomer. The linear starting materials for such reactions are typically easy to prepare from simple building blocks, with the synthesis of 16 macrocyclic products using extended CRE reactions of this type reported to date. This includes lactone **63**, which was formed via three consecutive ring expansion steps, to afford 17-membered macrocycle **63**, with selenide, sulfide, and tertiary amine groups each proposed to act as internal nucleophiles in the cascade ring expansion.

**Scheme 11:**
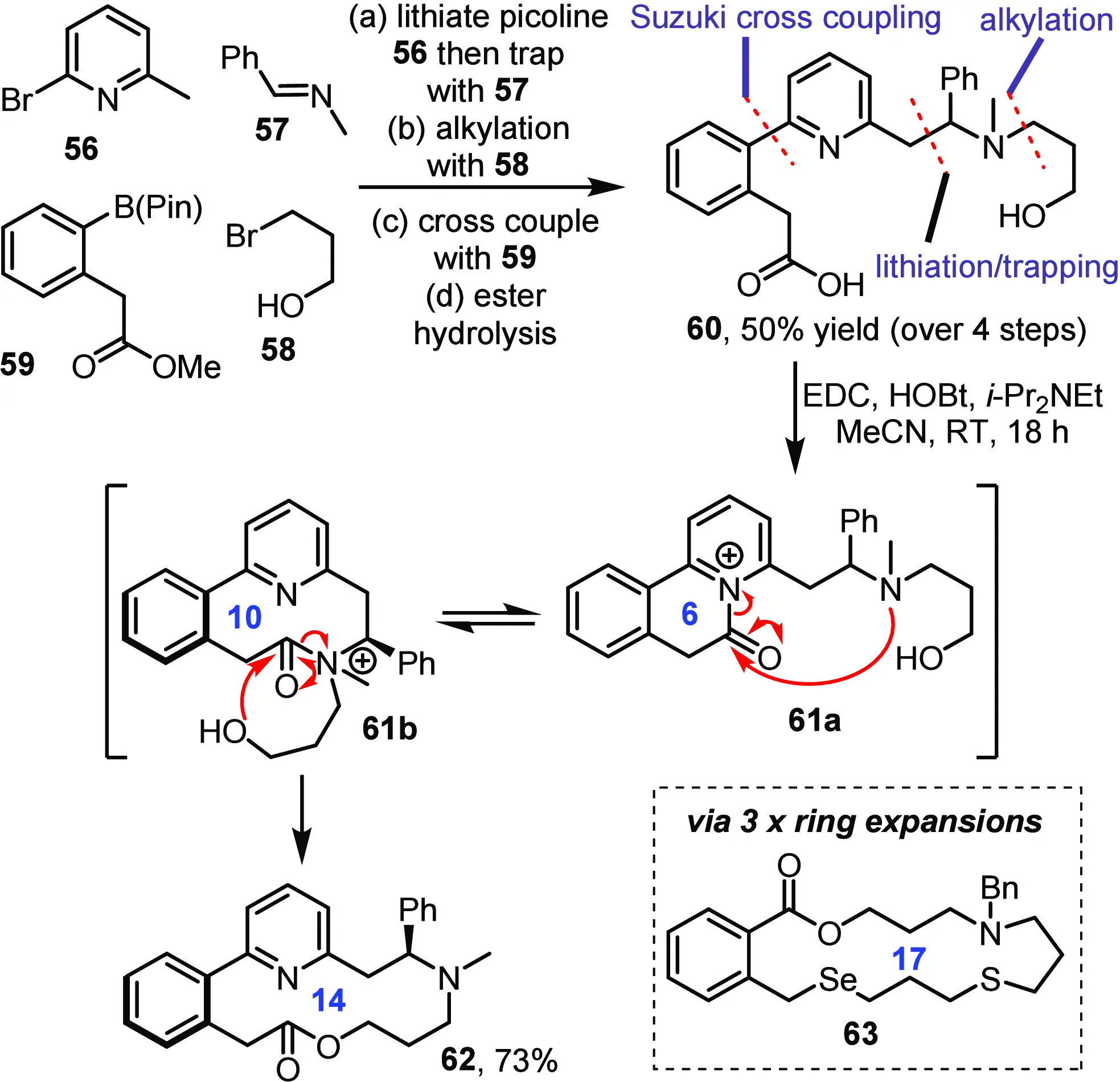
Extended Internal Nucleophilic Catalyst-Mediated Cyclization/Ring Expansion Cascades

## 3 Iterative Ring Expansion Reactions

This section is focused on the strategies for the iterative ring expansion of cyclic molecules. The ability to perform variants of the same class of ring expansion iteratively represents a powerful strategy for the synthesis of medium-sized rings and macrocycles, enabling their assembly via the controlled insertion of linear fragments into cyclic starting materials, to access ring-enlarged products. All of the iterative ring expansion methods that follow share the same key design feature. Following ring expansion, the key reactive functional group in the cyclic starting material that enabled ring expansion to occur must be retained or regenerated to enable subsequent iterations of ring expansion. The regeneration of the reactive functional group can occur directly during the ring expansion or following additional functional group interconversions. For functional group regeneration to occur alongside efficient ring expansion, careful reaction design is required. We therefore aim to highlight the most important design features of the innovative iterative ring expansion strategies that follow.

### 3.1 Iterative 2,3-Sigmatropic Rearrangement Approaches

To the best of our knowledge, the first iterative ring expansion strategy used to form medium-sized rings and macrocycles was reported by Schmid and Schmid,^80^ who developed a highly innovative iterative cyclic sulfide expansion based on 2,3-sigmatropic rearrangements (Scheme 12). The sequence starts with *S*-allylation of α-vinyl cyclic sulfide **64** following reaction with allyl bromide under acidic conditions (**64** → **65**). Then treatment with aqueous potassium hydroxide promotes ylide formation (**65** → **66**) and 2,3-sigmatropic rearrangement (**66** → **67**) to deliver the ring-expanded product **67**. Because the product **67** contains the same α-vinyl cyclic sulfide motif found in the starting material **64**, further iterations of the same two-step sequence can be performed. Thus, it was shown that eight-membered cyclic sulfide **67** can be further expanded into 11-membered ring **70** and subsequently 14-membered derivative **71** following additional iterations.

**Scheme 12:**
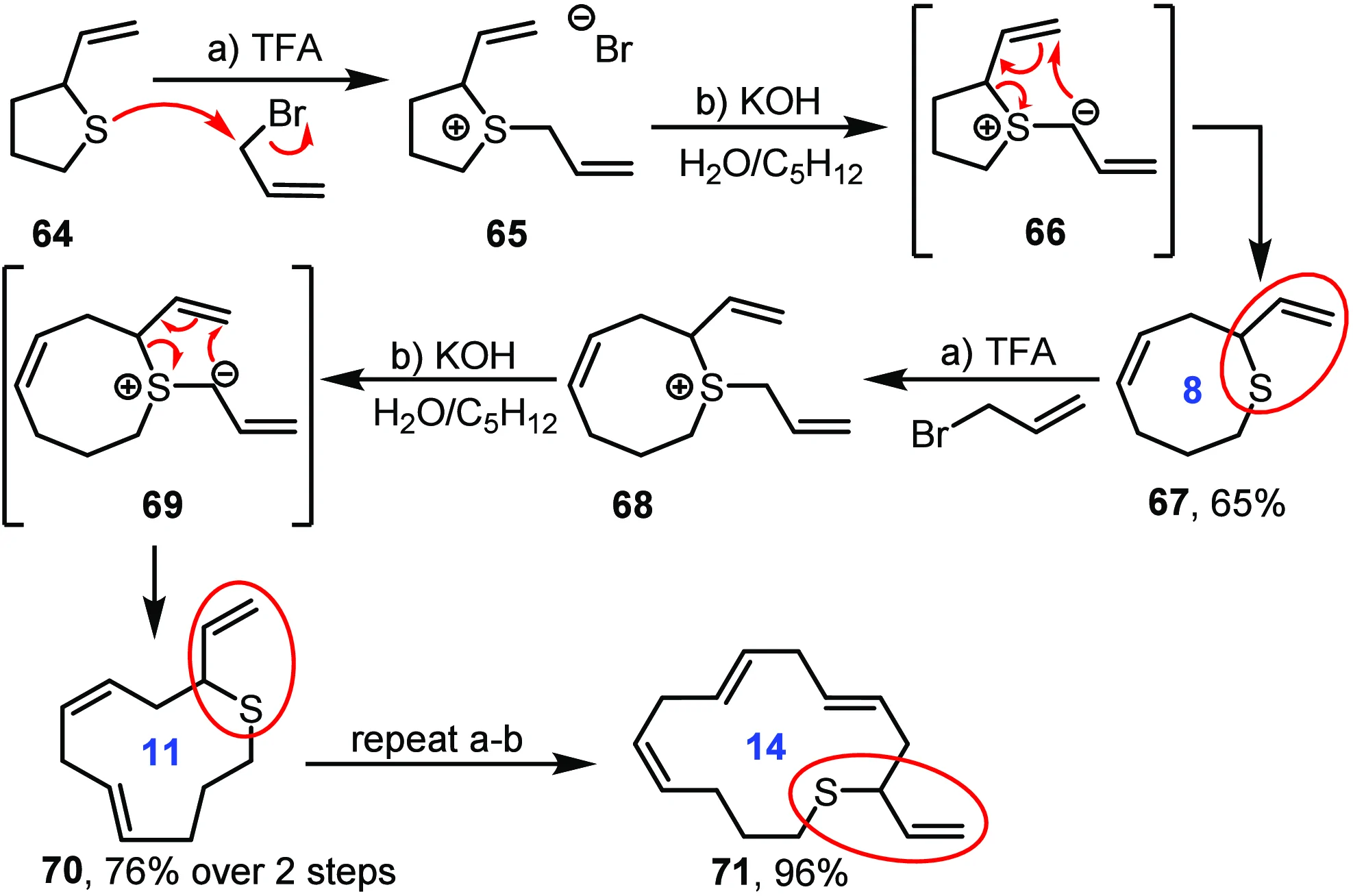
Iterative 2,3-Sigmatropic Rearrangements of Cyclic Vinyl Sulfides via *S*-Allylation

Vedejs and co-workers further developed the idea of using 2,3-sigmatropic rearrangements to enable iterative ring expansion (Scheme 13).^81–83^ This is exemplified by the sequence starting from α-vinyl cyclic sulfide **64**—the same starting material used in the previous work of Schmid and Schmid described in Scheme 12 above.^80^ Sulfide **64** was first converted into sulfonium salt **72** upon treatment with a diazocarbonyl reagent. Then, upon addition of base, the stabilized ylide **73** was formed, which undergoes spontaneous 2,3-sigmatropic rearrangement to afford ring-expanded cyclic alkene **74**. The rearrangement proceeds similarly to in the previous work from Schmid and Schmid,^80^ although in this system a Wittig reaction is needed to convert the ketone side chain of **74** into an alkene (**75**) to enable subsequent iterations. From **75**, a second iteration of the same ring expansion sequence was performed; in this case, a different diazo carbonyl species was used for the second iteration, but the chemistry proceed in broadly the same way; α-vinyl cyclic sulfide **75** was reacted with diazomalonate in the presence of copper at 100 °C to form 11-membered-ring product **77** via the same sequence of *S*-alkylation, ylide formation and 2,3-sigmatropic rearrangement.

**Scheme 13:**
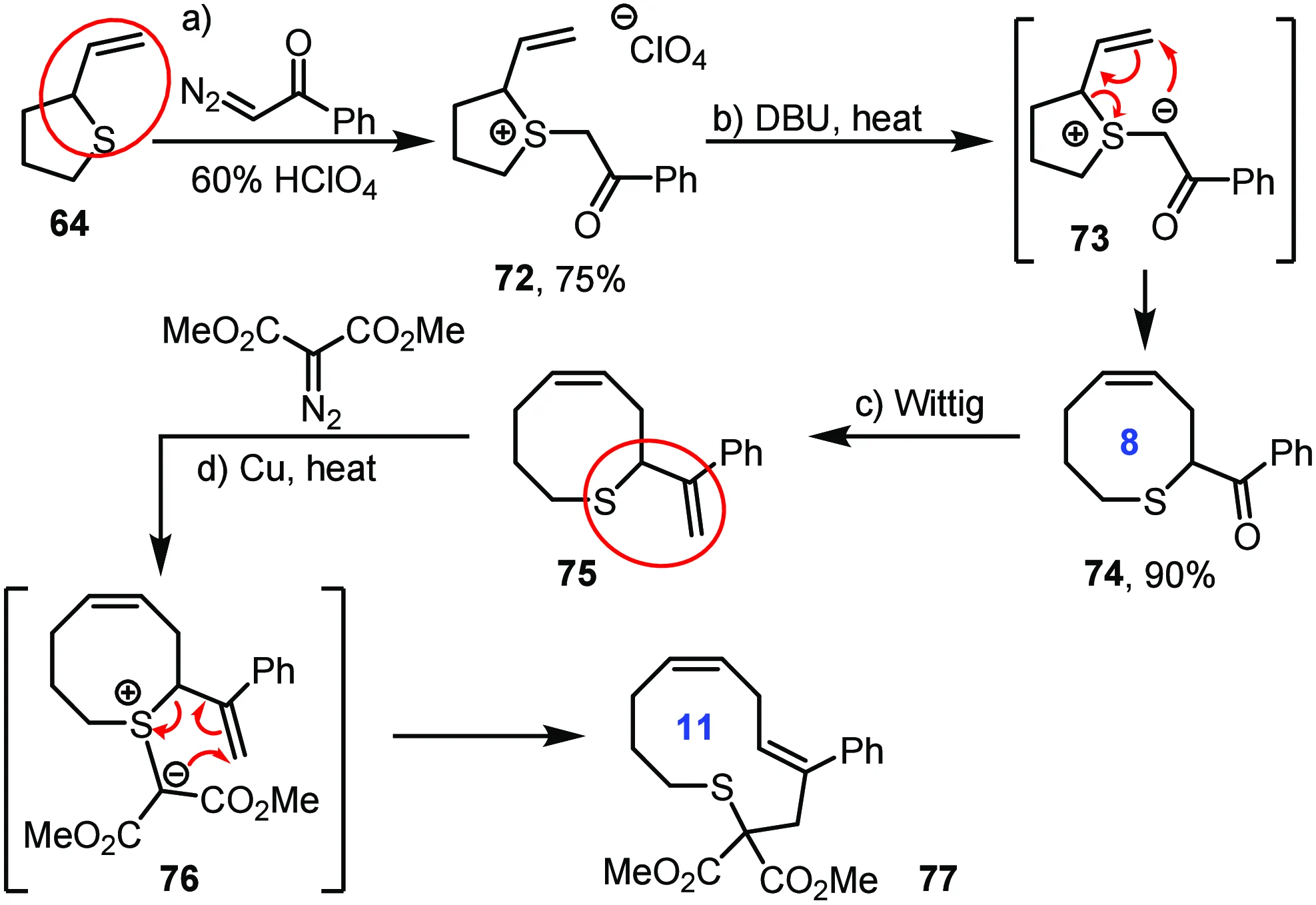
Iterative 2,3-Sigmatropic Rearrangements of Cyclic Vinyl Sulfides via Diazo Carbonyls

Compared with the earlier work by Schmid and Schmid, an extra step is needed to reform the alkene in Vedejs’ system. Nonetheless, the use of diazocarbonyl compounds enable different functionality to be introduced into the ring-enlarged products. Although not demonstrated, it is likely that both complementary methods could be applied within the same sequence.

### 3.2 Iterative One-Carbon Homologation Methods

The one-carbon homologation of boracyclanes, developed by Brown and co-workers,^84–86^ is an excellent example of a well-designed iterative ring expansion strategy. This study expands on an approach introduced by Matteson and Ray.^87^ Thus, the treatment of *B*-methoxyborinane **78** with (chloromethyl)­lithium furnishes one-carbon ring-expanded homologue **80** directly via the formation and *in situ* rearrangement of boronate complex **79**, as shown in Scheme 14. With a new *B*-methoxyborinane formed directly as the product, subsequent iterations of the same procedure can then be performed to enable iterative ring expansion. Via this approach, seven-membered boracyclane **80** was converted into 12-membered **81e** via five iterative ring expansions, all proceeding in good yield. The ring expansion worked consistently well for all ring sizes, indicating that there is a strong driving force for rearrangement.

**Scheme 14:**
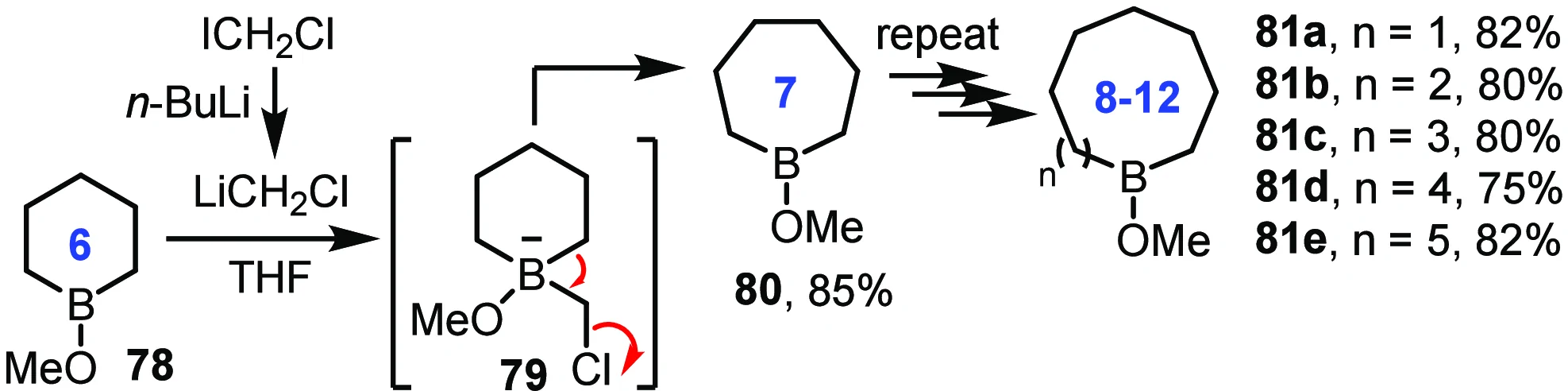
Iterative One-Carbon Homologation of Boracyclanes

Rousseau and co-workers^88^ developed a procedure for the iterative one-carbon ring expansion of lactones via sequential carbene-mediated cyclopropanation (Scheme 15). First, the seven-membered-ring lactone **82** is converted into silyl enol ether **83**. Then, reaction with ethyl carbene **84** (formed *in situ* from CH_2_CHCl_2_ and *n*-BuLi) affords cyclopropane **85**, which rearranges upon heating at reflux in toluene, to undergo desilylation and ring expansion (**85** → **86**). Then, following alkene hydrogenation to form **87**, the system is set up for another ring enlargement iteration to be performed; thus, the eight-membered lactone can then re-enter this ring expansion sequence, allowing subsequent iterations to be performed with chlorocarbene reagent **84** or **88**, enabling the synthesis of 9- and 10-membered lactones **89** and **90** in the same way.

**Scheme 15:**
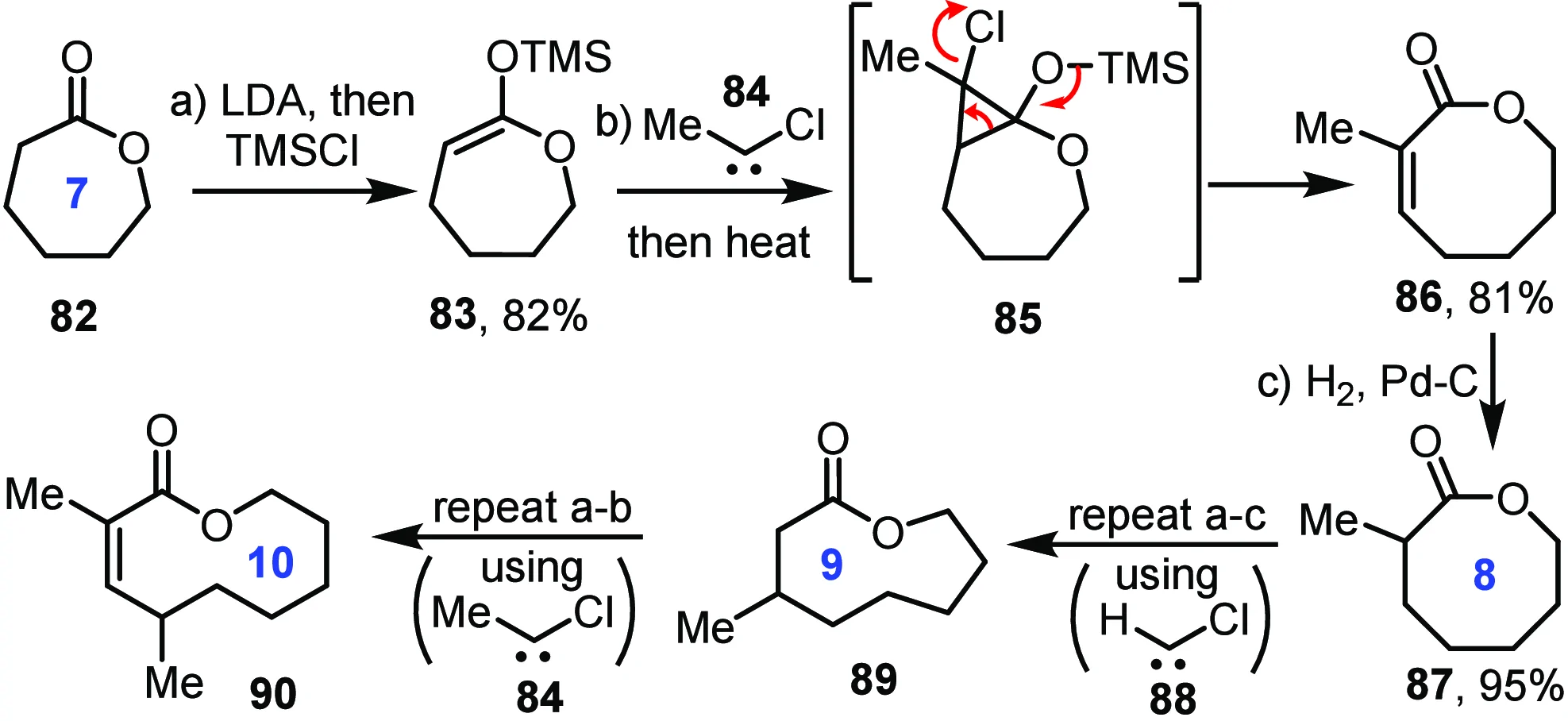
Iterative Ring Expansion of Lactones via Carbene-Mediated Cyclopropanation

### 3.3 Iterative Aza-Cope and Aza-Claisen Rearrangements

An iterative 3,3-sigmatropic rearrangement approach was described by Back and co-workers^89^ during their total synthesis of motuporamine A (Scheme 16). The sequence started with the reaction of 2-vinylpyrrolidine **91** with an acetylenic sulfone to form zwitterionic reactive intermediate **92**, which then underwent a 3,3-aza-Cope rearrangement *in situ* to form ring-expanded enamine product **93**. To enable a second iteration, the installation of a new exocyclic alkene group into this product was required, which was done via sequential hydrogenation (**93 → 94**), treatment with triflic acid, and the addition of a vinyl Grignard reagent to afford **95**. Removal of the tosyl group was then performed using a sodium–mercury amalgam to form **96**. Following these additional steps, the resulting product **96** then contains the required vinyl group (circled) needed for a second iteration of the conjugate addition/aza-Cope rearrangement sequence to be performed, thus enabling its ring expansion into 13-membered macrocyclic enamine **98**. An additional five steps (not shown) were then performed to complete the total synthesis of motuporamine A (**99**).

**Scheme 16:**
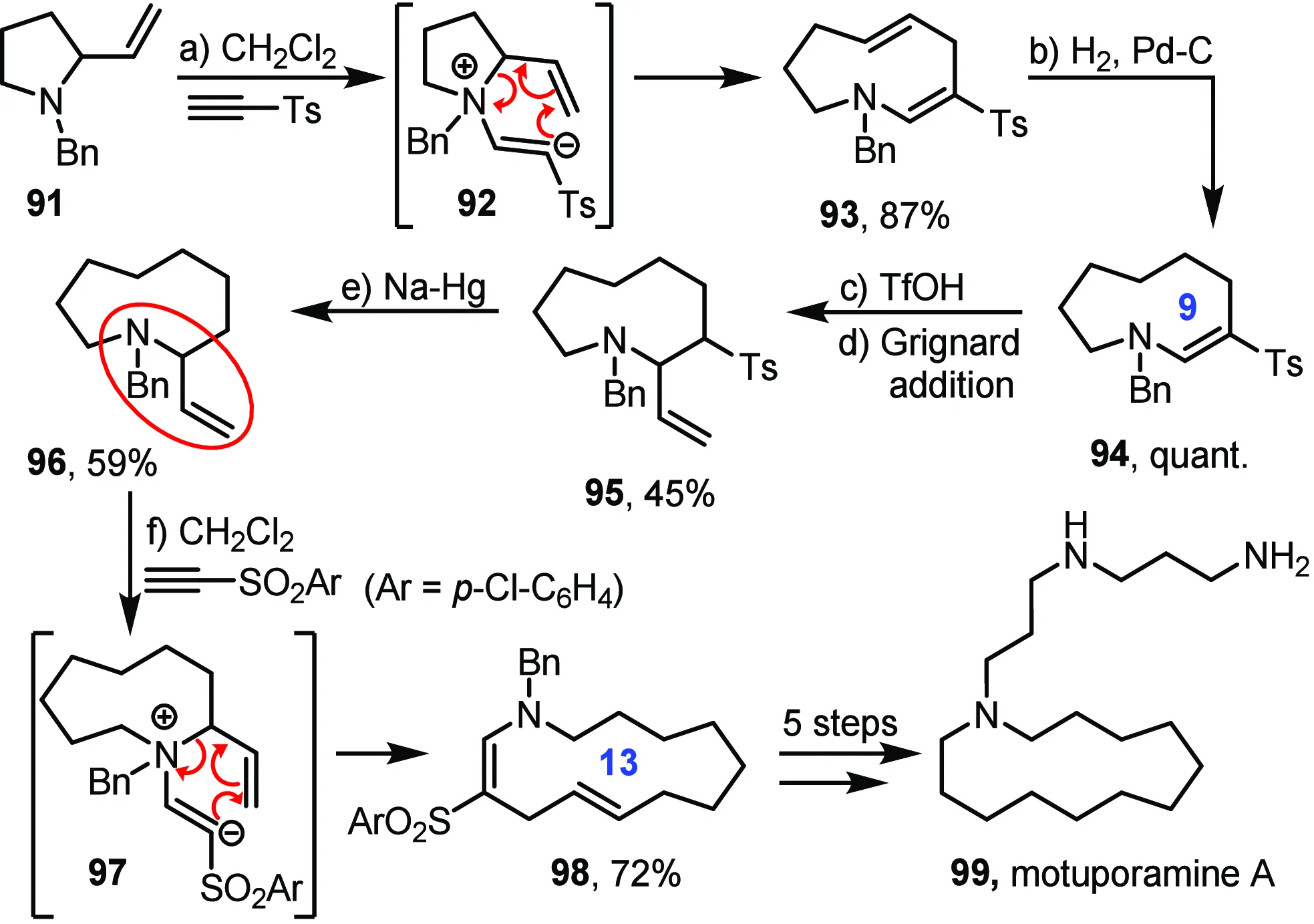
Iterative 3,3-Aza-Cope Rearrangement Ring Expansion Reactions

Suh and co-workers utilized iterative stereoselective aza-Claisen rearrangement reactions during their total syntheses of fluvirucinines A_1_ and A_2_ (Scheme 17).^90–92^ The sequence started from piperidinone **100**, which was synthesized by an asymmetric Evans alkylation and stereoselective vinylation. In the key step, treatment with LiHMDS forms enolate **101**, which rearranges into lactam **102** via a stereospecific 3,3-sigmotropic rearrangement. Before the second iteration could be performed, an eight-step sequence was then required to convert **102** into **103**. LiHMDS was again then used again to form the amide enolate and initiate the second aza-Claisen rearrangement, which was successful in forming 14-membered ring **105** with >10:1 diastereoselectivity. Additional steps from **105** were also performed to form natural products fluvirucinine A_1_ and A_2_ (not shown).

**Scheme 17:**
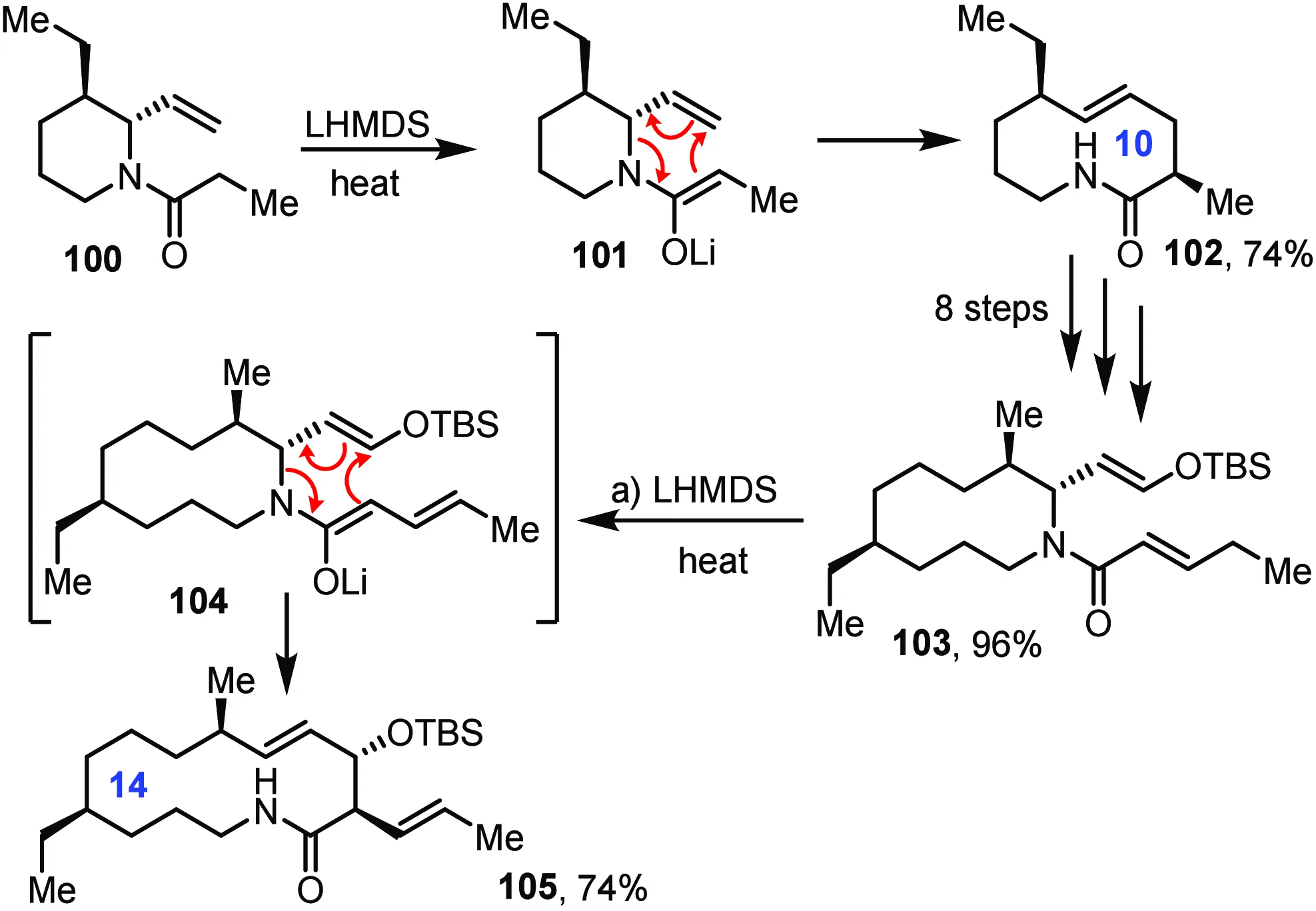
Consecutive Aza-Claisen Reactions in the Total Synthesis of Fluvirucinine Natural Products

### 3.4 Iterative Two-Carbon Homologation Methods

Yoshimi and co-workers built upon their original methodology involving photoinduced decarboxylative radical macrolactonization^93^ to allow the iterative two-carbon ring expansion of macrocyclic lactones.^94^ As shown in Scheme 18A, this allowed conversion of lactone **106** into its 18- and 20-carbon homologues **107** and **108**. The three-step overall ring expansion sequence operates via initial lactone hydrolysis (ring opening), reaction with acryloyl chloride, and radical-mediated photocyclization of the carboxylic acid tethered acrylate ester **110**. The ring expansion of **107** to the 20-membered-ring lactam **108** was also possible, albeit without the possibility of subsequent expansion. Similarly, starting with the corresponding 17-membered lactone, the analogous 19- and 21-membered systems were also accessible (not shown).

**Scheme 18:**
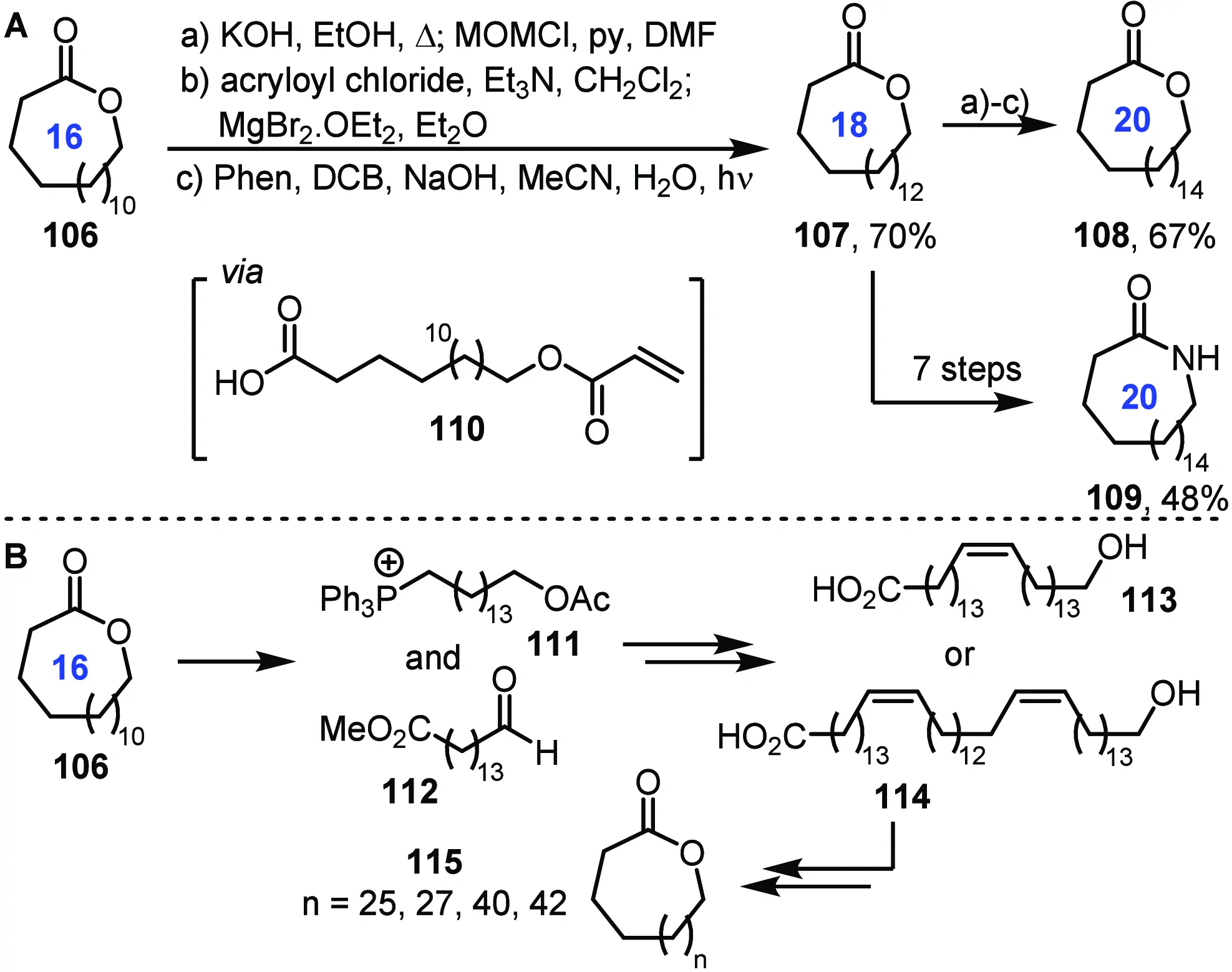
(A) Iterative Two-Carbon Ring Expansion of Macrocyclic Lactones; (B) Extensions to Larger Macrocycles

Further developments to this approach (Scheme 18B) have involved the synthesis of building blocks **111** and **112** from lactone **106**, which enabled the synthesis of a range of lactones of ring sizes 31 to 48, via seco acid intermediates of type **114**.^95^ Use of alternative building blocks allowed the formation of smaller ring sizes in the range 23 to 29.^96^ All such routes require multiple steps per iteration, and require ring opening and reclosure as part of each ring expansion process. The ring closure step makes use of either a Yamaguchi macrolactonization^97^ or a photoinduced decarboxylative radical macrolactonization at relatively high dilution, depending on the ring size desired. Thus, while both cyclization steps are relatively high yielding (typically 70–85%), this needs to be considered against the requirement to use a high dilution macrocyclization method.

A powerful example of a iterative two-carbon ring expansion was reported by Zhao, Li, and Sun (Scheme 19A).^98^ Thus, it was shown that upon reaction with a Lewis acid, cyclic acetal derivatives (**116**, where X = O) can be converted into two-carbon homologated products **121** via oxonium formation (**118**, X = O) followed by a formal [2 + 2] cycloaddition and electrocyclic ring-opening sequence. The same group later went on to develop an analogous protocol for the formation of homologated lactams from hemiaminal precursors (**116**, X = NTs).^99^ Both methods work across a range of ring sizes, and the approach has also been demonstrated to perform well iteratively (Scheme 19B). For example, five-membered acetal **122** was expanded into seven-membered lactone **124** via the method described, using alkyne **123**. Then, following hydrogenation, lactone reduction, and acetylation, the new acetal derivative **125** was formed, enabling a second iteration using the same method and alkyne **126** to afford nine-membered-ring lactone **127**.

**Scheme 19:**
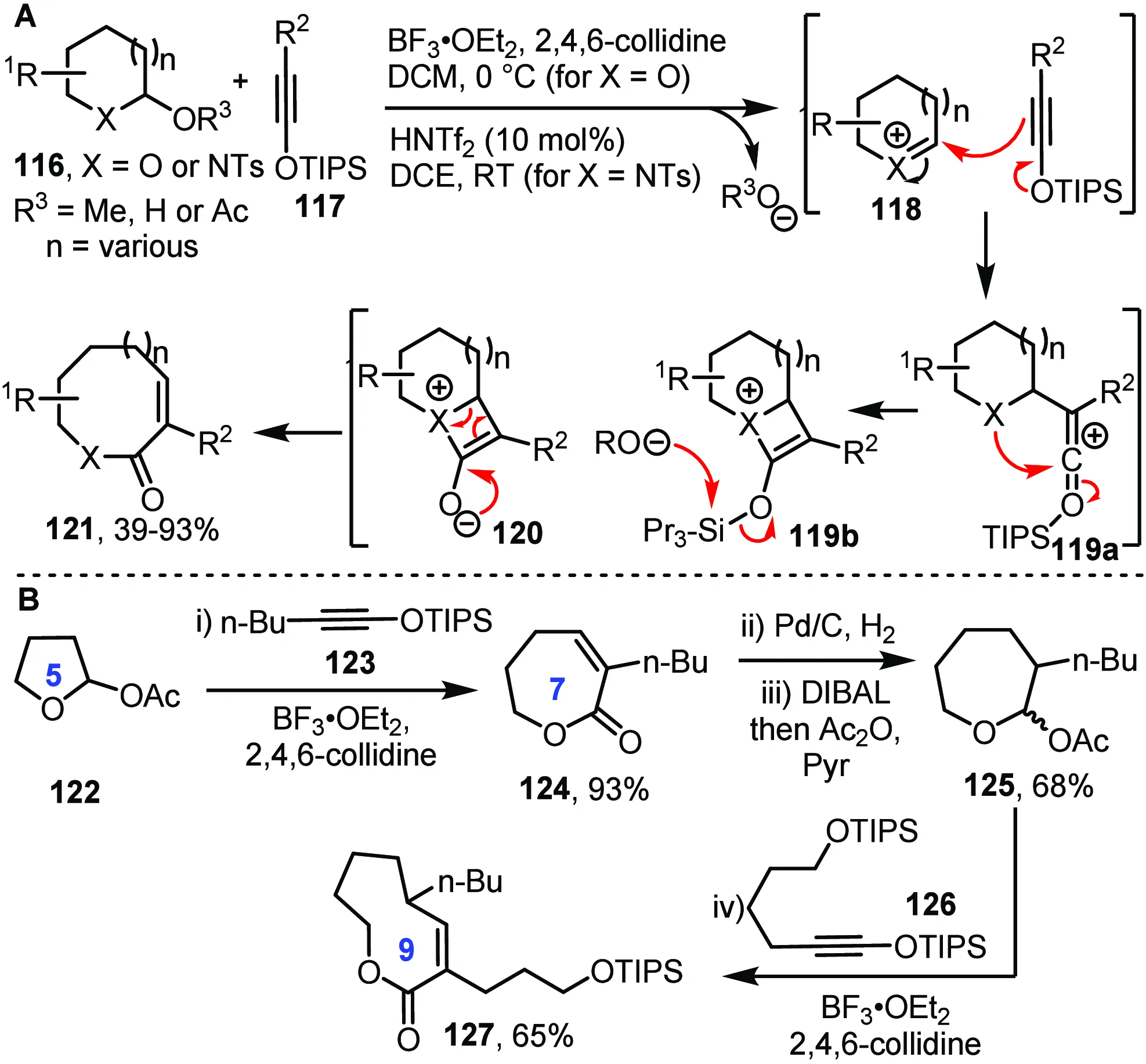
Two-Carbon Homologation of Cyclic Acetal Derivatives

### 3.5 Iterative Side-Chain Insertion of Amino Acids and Hydroxy Acids

A method for the iterative three- and four-atom ring reaction expansion of cyclic β-keto esters introduced by Unsworth and co-workers is summarized in Scheme 20A.^100–102^ In this sequence, the reaction of 12-membered-ring cyclic β-keto ester **128** with Fmoc-β-ala-Cl **129** under basic conditions with MgCl_2_ as an additive enables the *C*-acylation reaction to form **130**. Then, treatment with piperidine initiates a cascade ring expansion via cleavage of the Fmoc protecting group to form amine **131a**, followed by spontaneous cyclization and fragmentation to afford 16-membered-ring product **132** in 80% overall yield. The formation of an amide bond and a stabilized β-keto ester carbanion during the fragmentation step provides a strong thermodynamic driving force for ring expansion.^102^ This feature enables use of the method to expand to a wide range of cyclic β-keto esters with a range of ring sizes and substitution patterns. The fact that the β-keto ester motif in the starting material **128** is regenerated in the ring-expanded product **132** is important, as it enables the subsequent iterations of the same method to be performed straightforwardly (e.g., **132** → **133** → **134**). The same approach can also be used to prepare ring-expanded lactone products by replacing the Fmoc-protected amine with a benzyl-protected alcohol and revealing the alcohol using hydrogenolysis (not shown).^100^ The iterative application of the lactam- and lactone-forming variants enables relatively complex macrocycles to be formed via the insertion of different linear fragments, such as macrocycle products **135** and **136** (Scheme 20B).

**Scheme 20:**
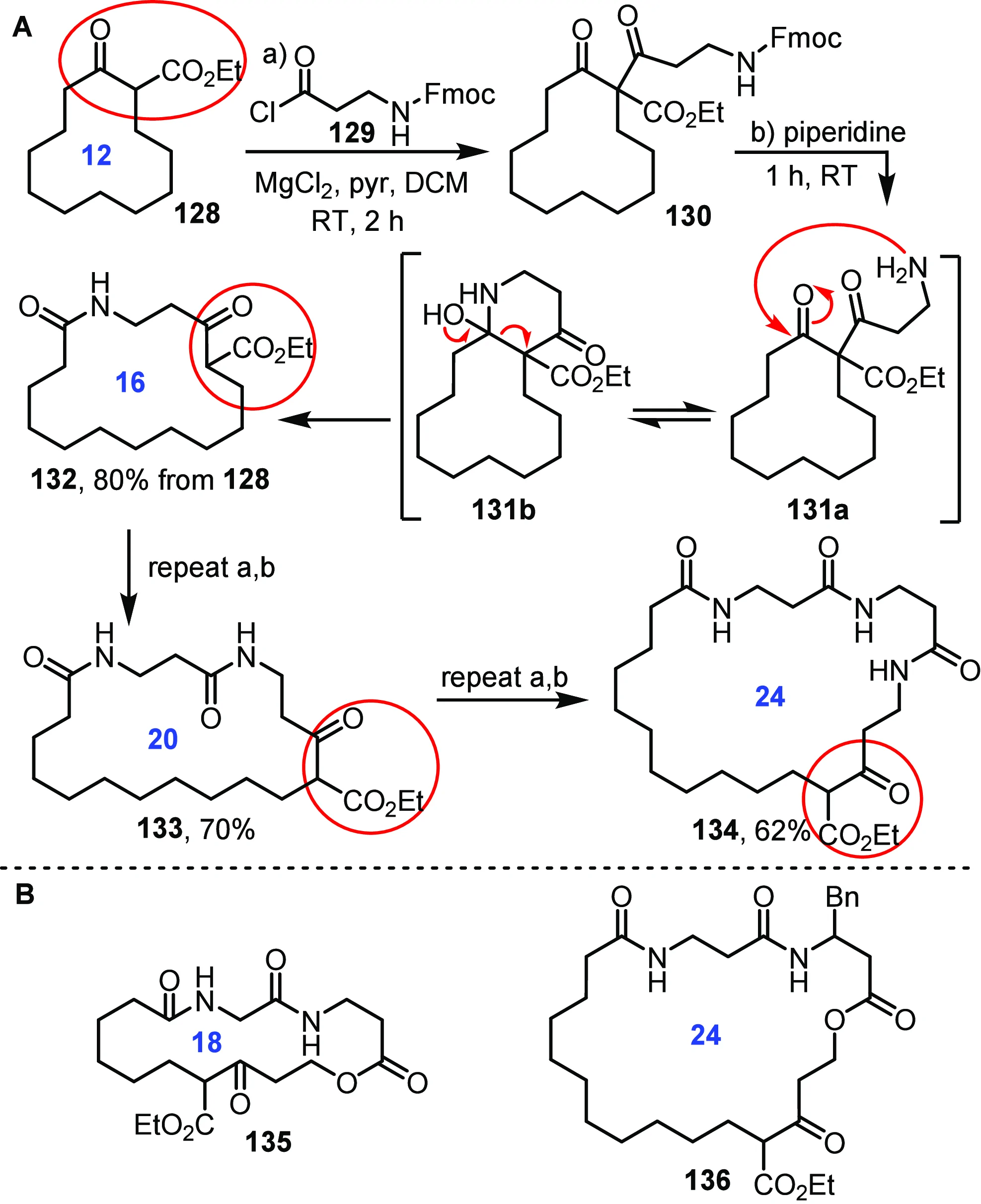
(A) Iterative Ring Expansions of Cyclic β-Keto Esters; (B) Example Products

The approach described in Scheme 20 was subsequently adapted to enable the iterative three- and four-atom ring expansion of lactam derivatives.^103^ A representative example of this approach is summarized in Scheme 21. In this case, *N*-acylation of lactam **137** with acid chloride **138** affords imide intermediate **139**, which is primed to undergo ring expansion following Fmoc protecting group cleavage, via **140a** and **140b**, to form **141**. The rearrangement results in the overall conversion of an imide and an amine (in **140a**) into two stable amide bonds (in **141**), which provides a clear thermodynamic driving force for ring expansion. Regeneration of the NH lactam group (circled) directly following the ring expansion enables further iterations of the *N*-acylation and ring expansion sequence (e.g., **141** → **142** → **143**).

**Scheme 21:**
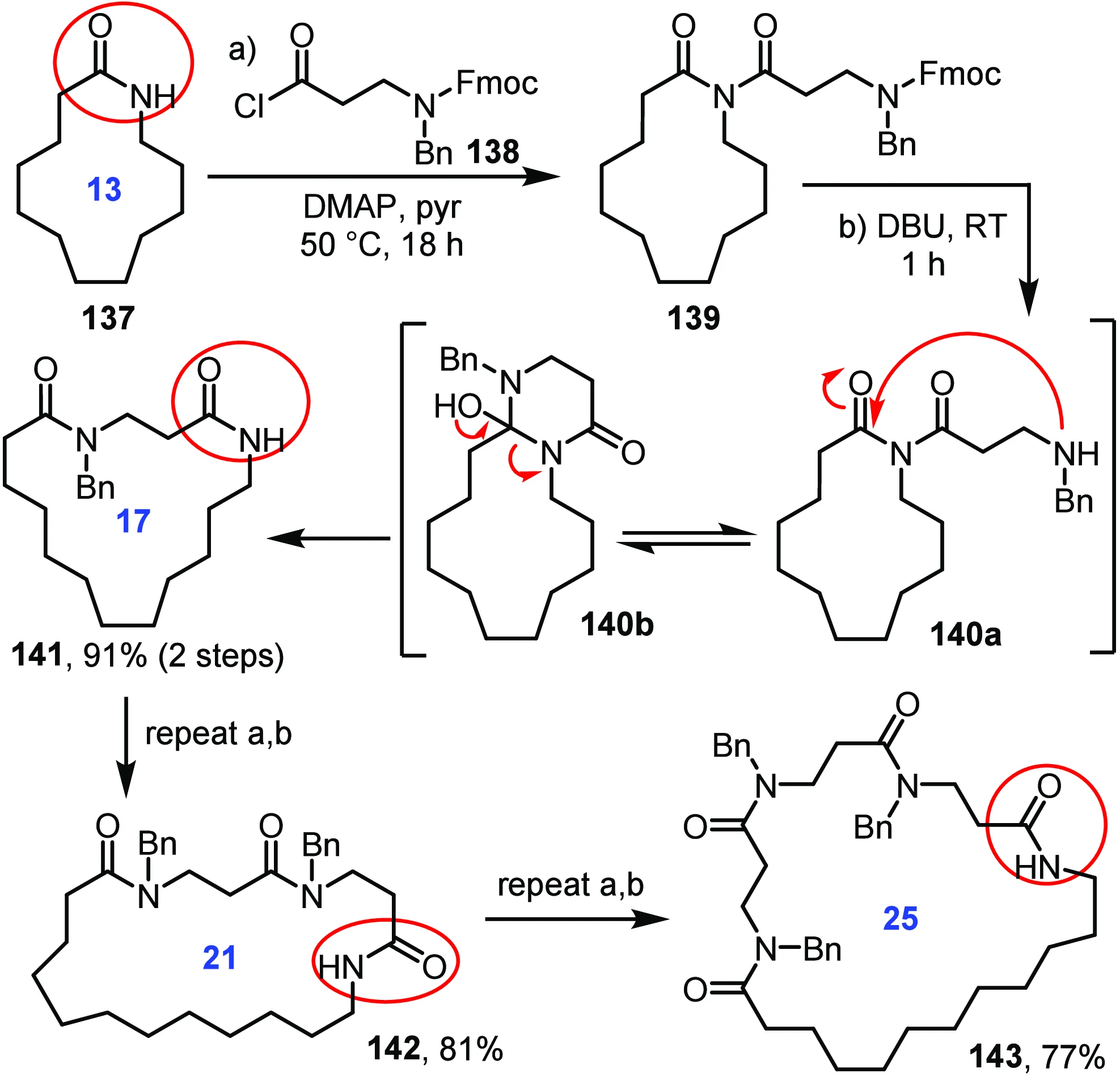
Iterative Ring Expansions of Lactams

Several other ring expansion reactions of this type have been reported by the same team (Scheme 22). Variants based *N*-acylation with acid chlorides containing protected amines (to form lactams),^103^ alcohols (to form lactones),^104^ and thiols (to form thiolactones)^105^ have all been described (Scheme 22A). These methods have been used to perform a range of three- and four-atom ring expansion reactions and generate a diverse array of macrocyclic products (e.g., **147**–**149**, Scheme 22B). The rearrangement reactions that lead to ring expansion are thought to be under thermodynamic control, and reaction outcomes can also be predicted based on the calculated ground state Gibbs free energies of the isomeric species involved the ring expansion cascade.^102^ The methods summarized across Schemes 20–22 are collectively called “successive ring expansion” (“SuRE”) methods in the publications describing their development.^100–106^

**Scheme 22:**
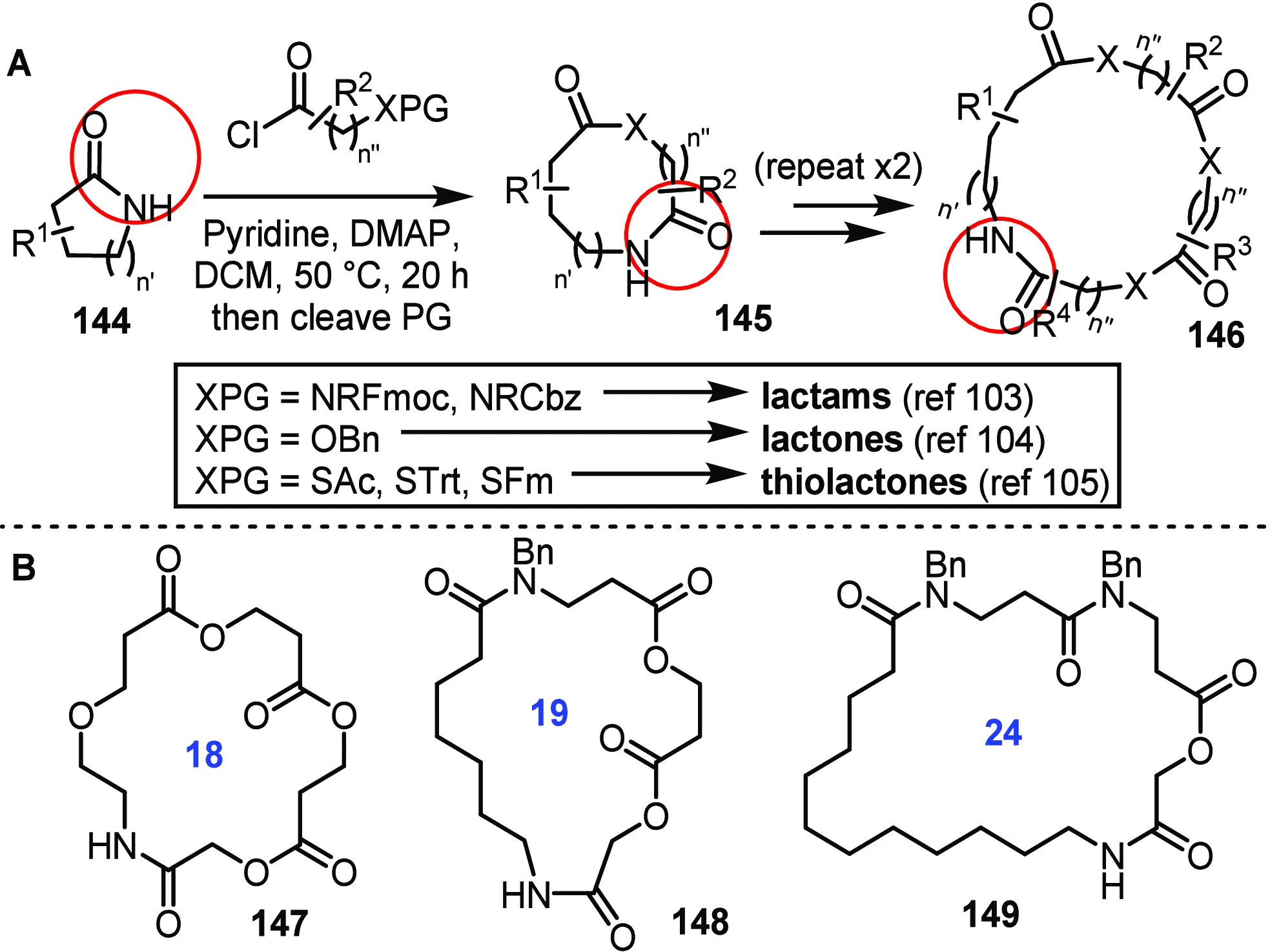
(A) Iterative Ring Expansions of Lactams; (B) Representative Products

To prepare several of the starting materials needed for the studies summarized in Schemes 21 and 22, iterative ring expansion reactions based on classical one-atom homologation methods were used (Scheme 23).^51–53^ To support the development of the new ring expansion methods, scalable routes to 9- to 11-membered-ring β-keto esters **151b**–**d** and lactams **152b**–**d** were required.^102,103^ This was achieved via a divergent, iterative ring expansion approach, starting from eight-membered cyclic ketone **150a**. Thus, using a procedure modified from that of Liu and Majumdar,^107^ ketone **150a** was reacted with ethyl diazoacetate and boron trifluoride diethyl etherate to form one-carbon homologated β-keto ester **151b** via a one-carbon Tiffeneau–Demjanov-type ring expansion homologation.^51^ Furthermore, the same starting ketone **150a** could also be converted into nine-membered lactam **152b** via a Beckman rearrangement, also via a one-atom ring expansion. To enable access larger ring sizes, β-keto ester **151b** was heated in wet DMSO, resulting in concomitant ester hydrolysis and decarboxylation to form nine-membered cyclic ketone **150b**. From this ketone, the same two ring expansion reactions could be repeated, affording 10-membered-ring systems **151c** and **152c**. Then, by performing the same hydrolysis/decarboxylation reaction on β-keto ester **151c** to form 10-membered ketone **150c**, ring expansion to form 11-membered-ring products **151d** and **152d** was also enabled.

**Scheme 23:**
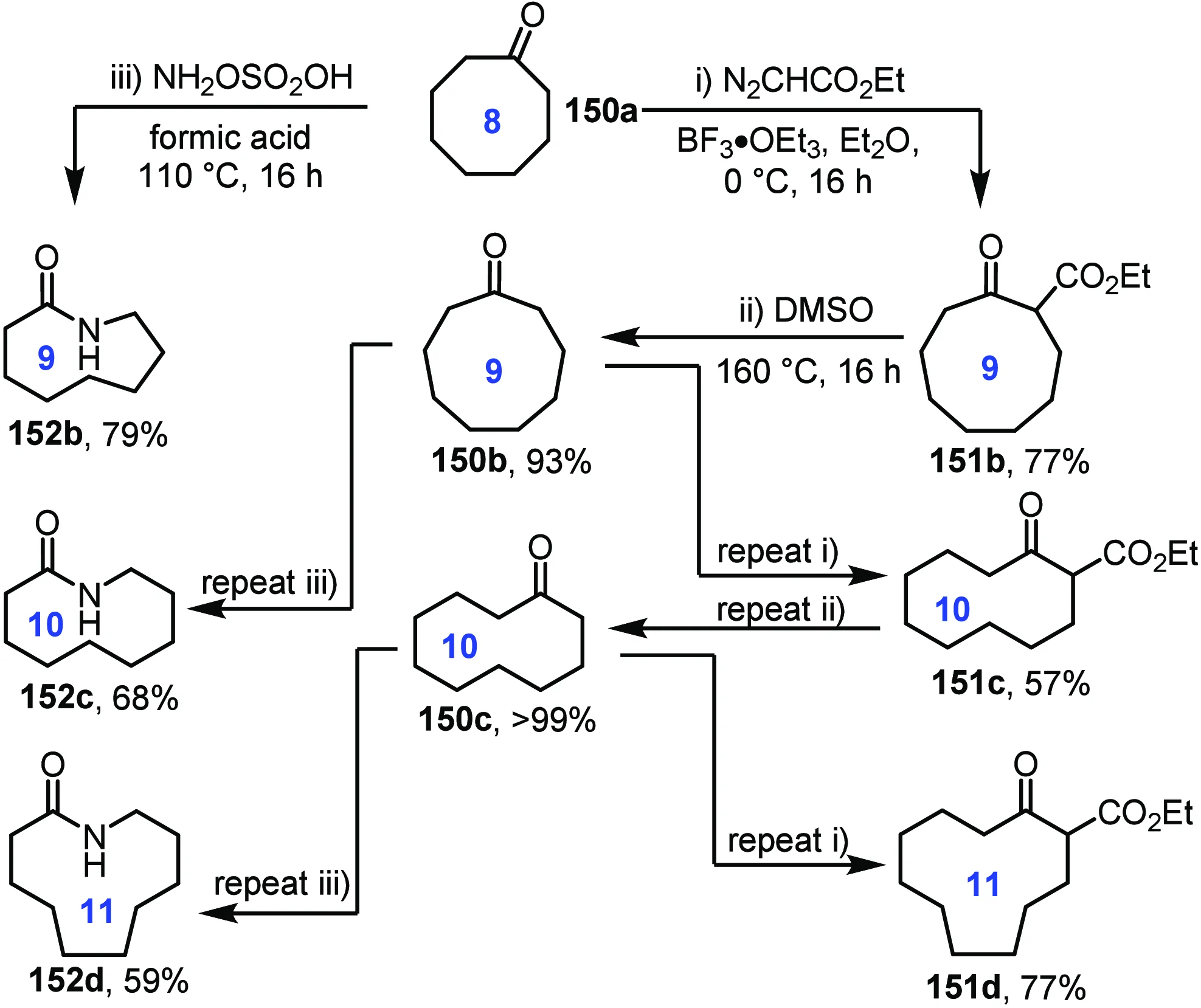
Iterative Homologation of Cyclic Ketones into Cyclic β-Keto Esters and Lactams

Scheme 24 summarizes a conjugate addition/ring expansion (CARE) cascade method that is capable of performing the iterative four-atom ring expansion reactions of lactams.^108–112^ The CARE method was introduced as a protecting-group-free extension to the SuRE methods described above and summarized in Schemes 21 and 22. This approach starts with *N*-acylation of a lactam staring material using acryloyl chloride **154** (Scheme 24A). The resulting imide **155** can then react with various primary amines, to undergo conjugation addition and form an intermediate amine **156a**, primed to undergo ring expansion *in situ* (**156a** → **156b** → **157**). The scope of the method is broad, especially with respect to the primary amine reaction partner, with a wide range of functionalized amines group compatible. This feature facilitates the efficient synthesis of large libraries of diversely functionalized medium-sized rings from commercially available primary amines and their salts. CARE reactions are typically performed in RT in methanol and are insensitive to air and moisture; indeed, the reaction even works using water as the reaction solvent. Following ring expansion, the amide motif (circled) in the starting material **153** is regenerated in the ring-expanded product **157**. Thus, the process can be performed iteratively to allow access to larger ring products; for example, macrocycles **158a**–**c** were each prepared from 2-piperidone following three iterations of the CARE sequence, using different amines for each iteration (Scheme 24B). Further extensions to the CARE approach are described later in the review (see Schemes 42, 43, and 47 in section 4).

**Scheme 24:**
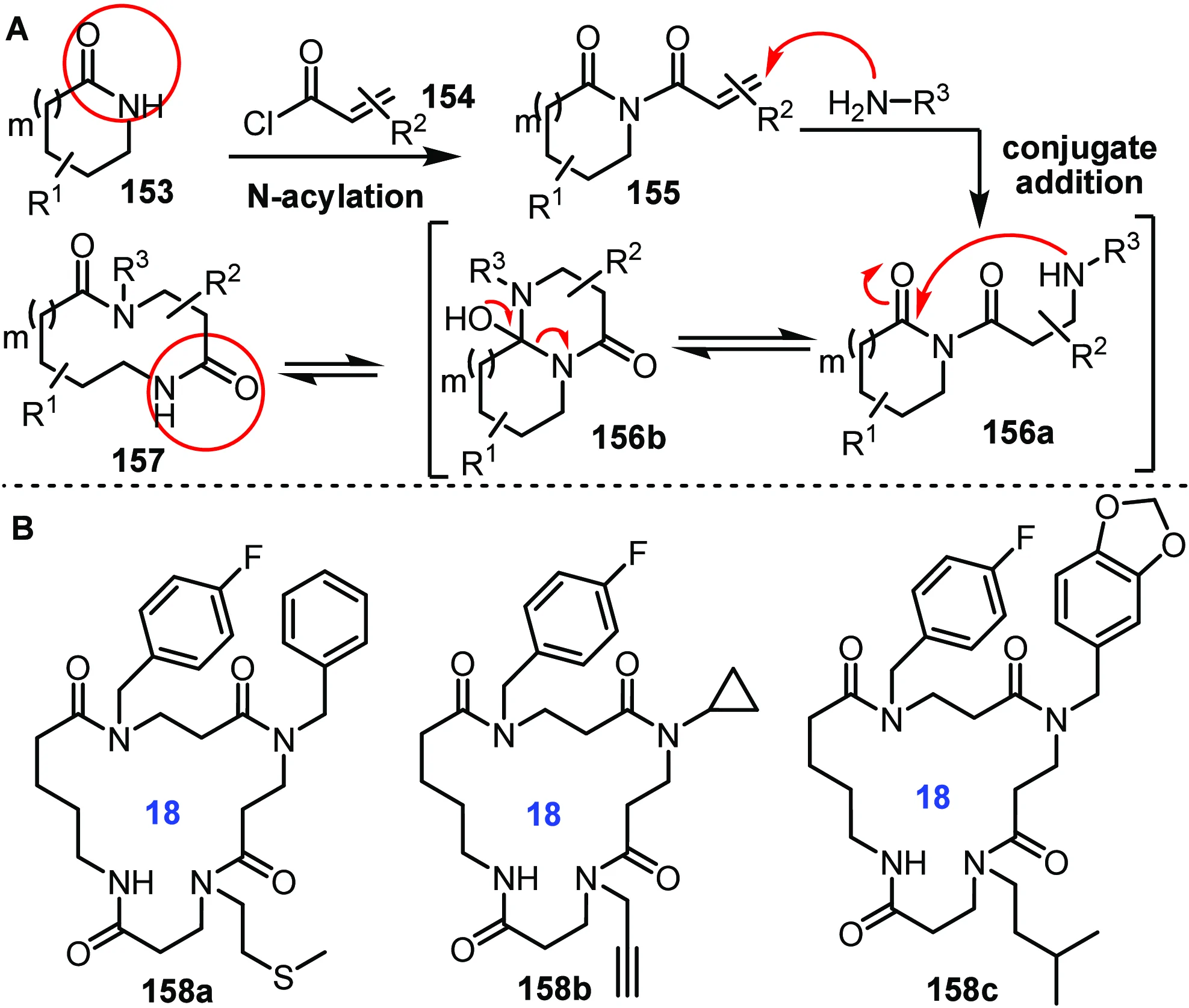
(A) CARE Reactions of Acryloyl Imides; (B) Representative Products Prepared by Iterative CARE Reactions

### 3.6 Iterative Homologation of Boranes and Amines

Williams and co-workers implemented an impressive one-pot iterative homologation method to expand 9-BBN, as part of their studies into hyperstable alkenes (Scheme 25).^113^ This approach is based on the iterative homologation method of Brown and co-workers described earlier (see Scheme 14). Thus, the reaction of 9-BBN derivative **159** with (bromomethyl)­lithium enables conversion into ring-expanded product **160**, now comprising a bridged bicycle made up of two seven-membered rings. Two further iterations using the same reagent then followed to promote further expansion (**160** → **161** → **162**). A fourth ring expansion was then performed, using alternative reagent (dichloromethyl)lithium to form bicycle **163**, and subsequent treatment with aqueous sodium hydroxide and hydrogen peroxide furnished the secondary alcohol **164** in an impressive 30% overall yield from 9-BBN. Homologue **167** was synthesized in much the same way, with an additional bromomethyl)lithium homologation (**162** → **165**) added to the sequence.

**Scheme 25:**
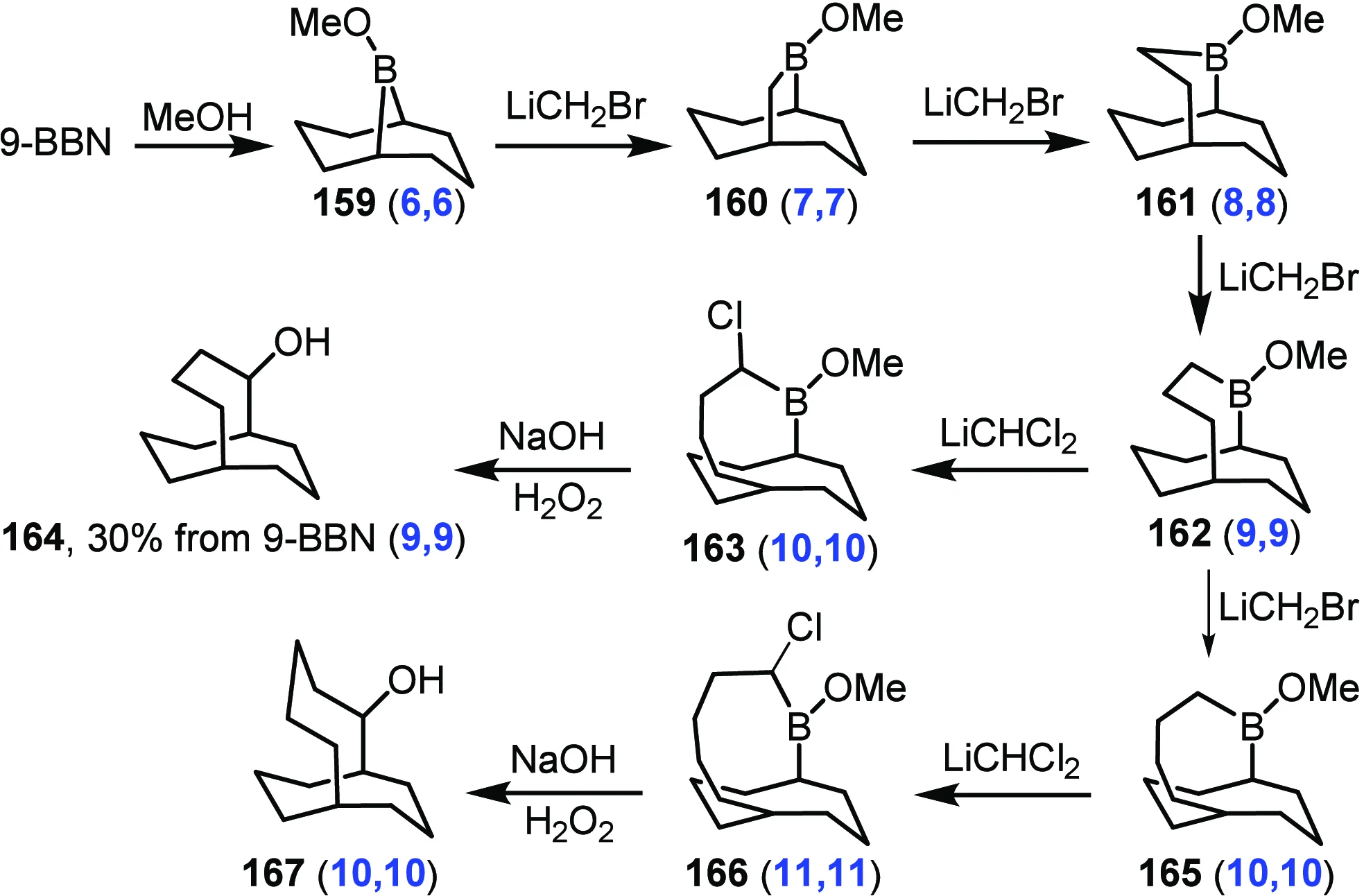
One-Pot Iterative Homologation of 9-BBN

Wang and co-workers developed an innovative method for modular alkyl growth in amines, via the selective insertion of alkynes into C–C bonds.^114^ In this study, various cyclic and acyclic amines were shown to undergo a two-atom homologation upon reaction with electron deficient alkynes. A representative example is summarized in Scheme 26. The reaction hinges on the use of a Lewis acidic borane catalyst, which serves to abstract hydride from the amine and enable a formal [2 + 2] cycloaddition to afford strained intermediate **169**, primed to undergo ring expansion to form eight-membered-ring product **170**. Subsequent one-pot ester hydrolysis, hydrogenation and decarboxylation then promotes conversion into cyclic amine **171**, resulting in the net insertion of two methylene groups with respect to the starting material **168**. Two further iterations of this ring expansion, hydrolysis and hydrogenation sequence enabled further ring expansion to sequentially form 10- and 12-membered cyclic amines **172** and **173** in good overall yields.

**Scheme 26:**
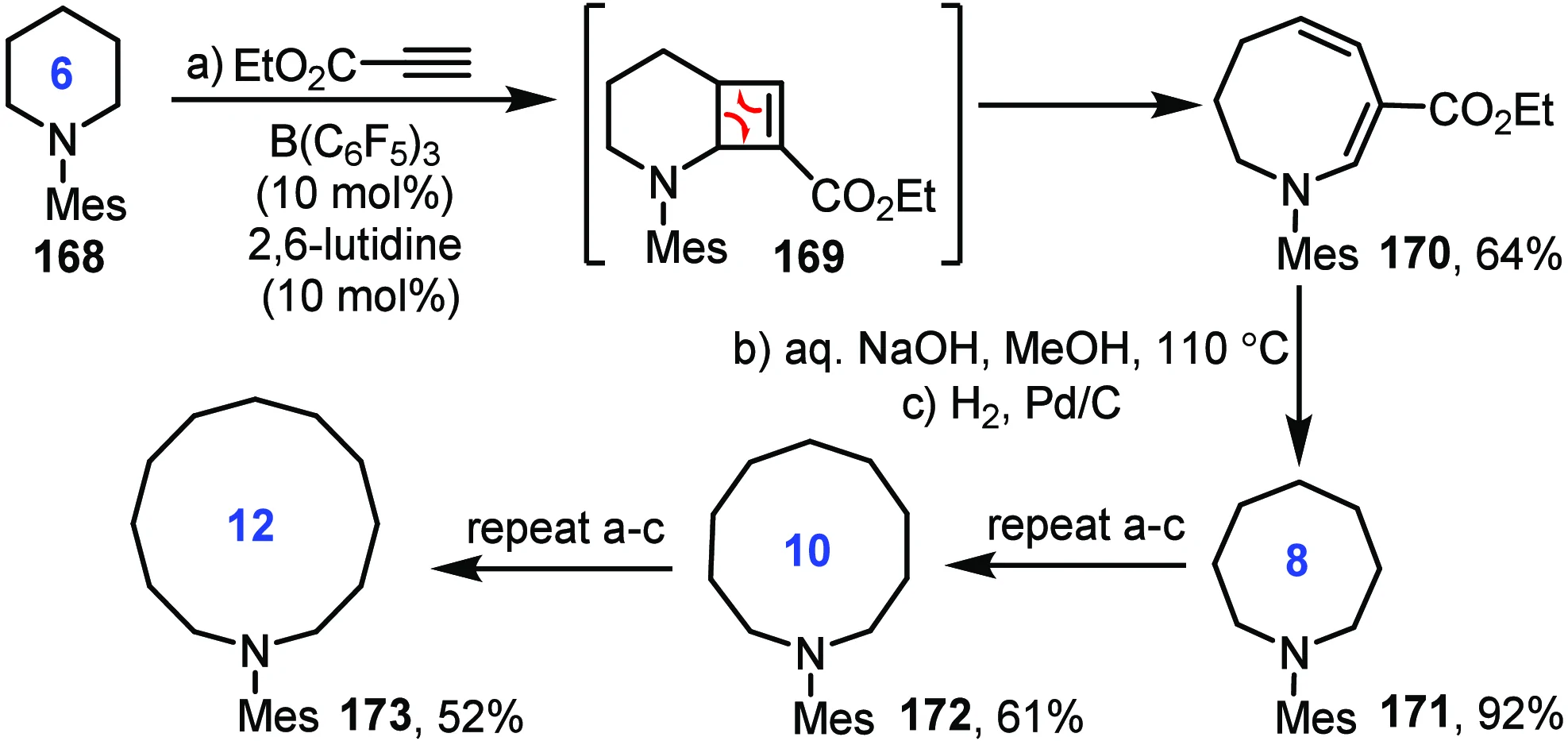
Modular Alkyl Growth in Cyclic Amines by Alkyne Insertion into C–C Bonds

### 3.7 Iterative Ring Expansion in Bioconjugation

The iterative CARE strategy introduced earlier in Scheme 24 was recently extended to enable its use for the selective bioconjugation of proteins on their N-terminus, to afford novel protein–macrocycle conjugates.^115^ The bioconjugation of several proteins was reported, exemplified in Scheme 27 in a sequence culminating in the modification of the human CCL5 chemokine—a protein which binds to the G-protein-coupled chemokine receptor CCR5. Using the standard CARE method, two 10-membered-ring products **175a** and **175b** were synthesized from six-membered-ring acryloyl imide **174.** Then, following conversion into imides **176a** and **176b**, each was used for the CARE modification of the N-terminus of CCL5 via a second ring expansion. In both cases, CARE provided access to 14-membered macrocycle-modified CCL5, bearing either an ethyl ester (**177a**) or an aryl fluoride (**177b**) side chain. When CCR5 stimulation by the macrocycle–CCL5 conjugates was compared to wild-type CCL5, similar agonistic abilities were observed, indicating that the macrocycles do not disrupt the binding interaction in the ligand pocket. Furthermore, the 14-membered aryl fluoride-modified macrocycle conjugate **177b** did not elicit any CCR5 phosphorylation of the Ser349 residue, which is the target of G-protein-coupled receptor kinases (GRKs) prior to agonist-mediated endocytosis.^116–118^ Thus, **177b** does not induce CCR5 phosphorylation but can still trigger CCR5 endocytosis. This demonstrates that the installation of macrocycles on CCL5 can decouple CCR5 phosphorylation and downmodulation requirements and constitutes a novel approach for dissecting chemokine ligand–receptor interactions.

**Scheme 27:**
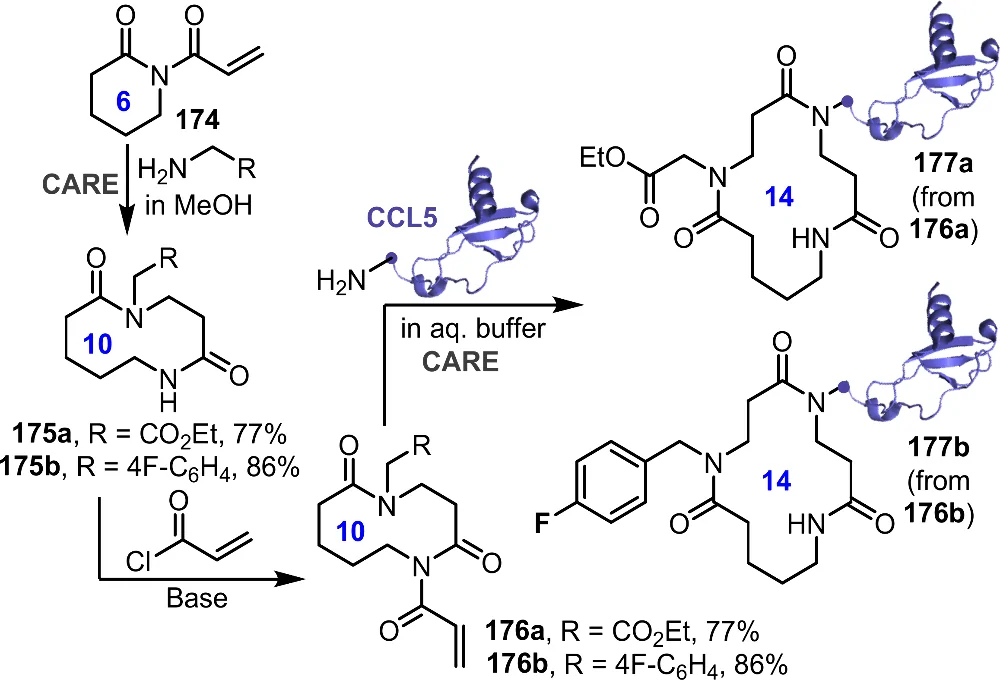
N-Terminal Protein–Macrocycle Conjugates Enabled by Conjugate Addition/Ring Expansion Cascade Reactions

## 4 Consecutive Ring Expansion Reactions

This section is focused on consecutive ring expansion strategies. As with the iterative ring expansion approaches described in section 3, this section focuses on strategies for the synthesis of medium-sized rings and macrocycles via the controlled and sequential insertion of linear fragments into ring-enlarged products. However, rather than performing the same (or very similar) ring expansion methods iteratively, this section describes methods in which more fundamentally different ring expansion methods have been applied consecutively. By using different ring expansion methods, the requirement to regenerate the key functional group needed to affect the first ring following ring expansion is removed. Thus, utilizing different ring expansion methods in this way opens up more opportunities to perform consecutive ring expansion reactions directly, often without the need for additional synthetic steps in between, by using reactive handles already present in the cyclic product following the first ring expansion. Therefore, of the three classes of successive ring expansion featured in this review (sections 2–4), this approach arguably has the greatest potential to be used to generate diverse functionalized medium-sized rings and macrocycles.

An early example of consecutive ring expansion reactions being used during a natural product synthesis can be found in the work of Schmid and co-workers in their total synthesis of spermidine alkaloid desoxo-indandenine (Scheme 28).^119^ Starting from 13-membered cyclic ketone **178**, sequential conjugate addition with acrolein and reductive amination afforded ketone **180**, which under mildly basic, aqueous conditions rearranged to ring-expanded product **181**. Next, hydrolysis and a reductive Nef reaction removed the nitro group to form lactam **182**, and electrolysis was used to remove the tosyl groups to form **183**. This set up the second ring expansion, where treatment of the resulting diamine with *p*-toluenesulfonic acid (*p*-TSA) at reflux in xylene produced a 1:1 mixture of 17- and 21-membered lactams **183** and **184**.

**Scheme 28:**
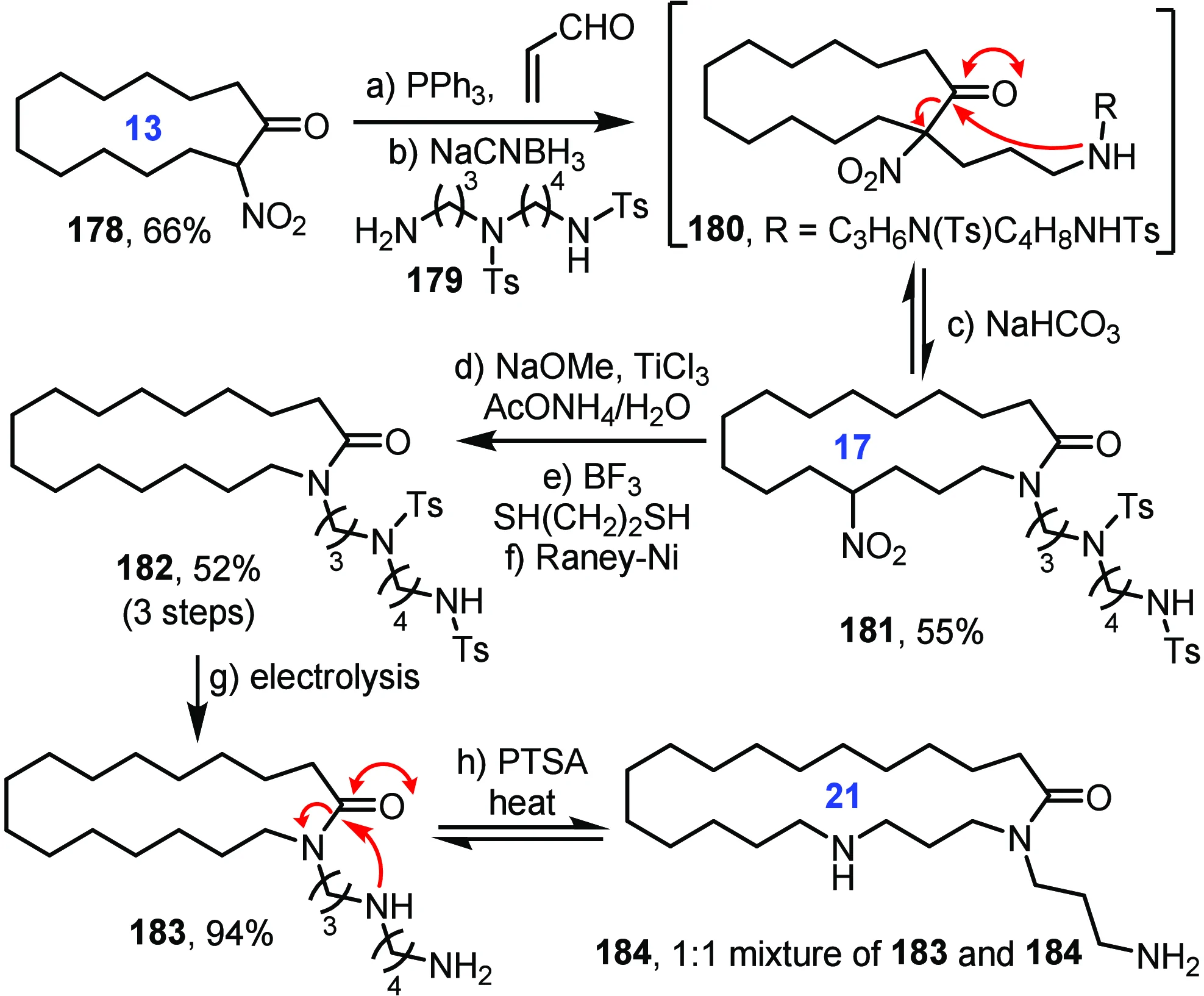
Synthesis of 21-Membered Lactam via Consecutive Reversible Ring Expansion Reactions

Wasserman and co-workers reported an impressive consecutive ring expansion sequence during their total synthesis of celacinnine,^120^ an important macrocyclic alkaloid in the spermidine family of natural products (Scheme 29).^121–123^ Two different reductive methods for the ring expansion of bicyclic systems were reported to generate nine-membered ring **191**. One of these methods was the reduction of cyclic amidine **185** with sodium cyanoborohydride. The same product **191** could also be formed via the reductive N–N bond cleavage of bicyclic compound **190**, itself formed from ethyl cinnamate **188** and cyclic hydrazine **189**. Selective alkylation of the amide group of **191** was possible under strongly basic conditions, and following phthalimide cleavage using hydrazine and reaction with NaOH, a transamidation reaction took place to the form 13-membered-ring spermidine skeleton **195** in good yield. Acylation with cinnamyl chloride then completed the total synthesis of celacinnine **196**.

**Scheme 29:**
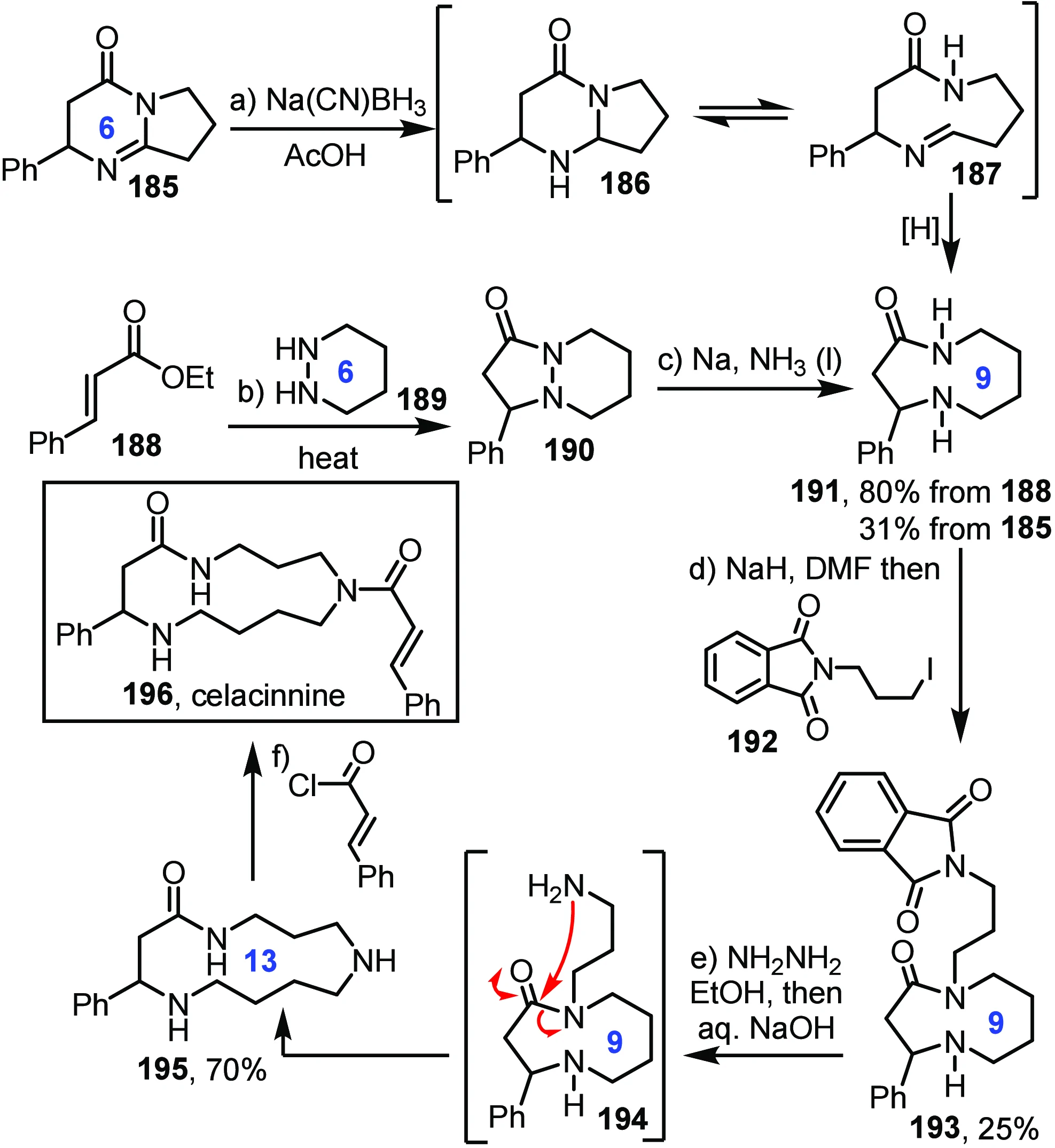
Sequential Ring Expansion Reactions in the Total Synthesis of Celacinnine

An impressive example of the use of sequential ring expansion reactions was reported by Ikeda and co-workers.^124^ In this work, a Grob-type fragmentation to form eight-membered-ring cyclic diene **200** was followed by an oxidative fragmentation to form macrocycle **201**, an advanced precursor to the natural product (±)-phoracantholide M (**202**). The key cyclobutane intermediate **198** was formed via a [2 + 2] cycloaddition reaction (Scheme 30).

**Scheme 30:**
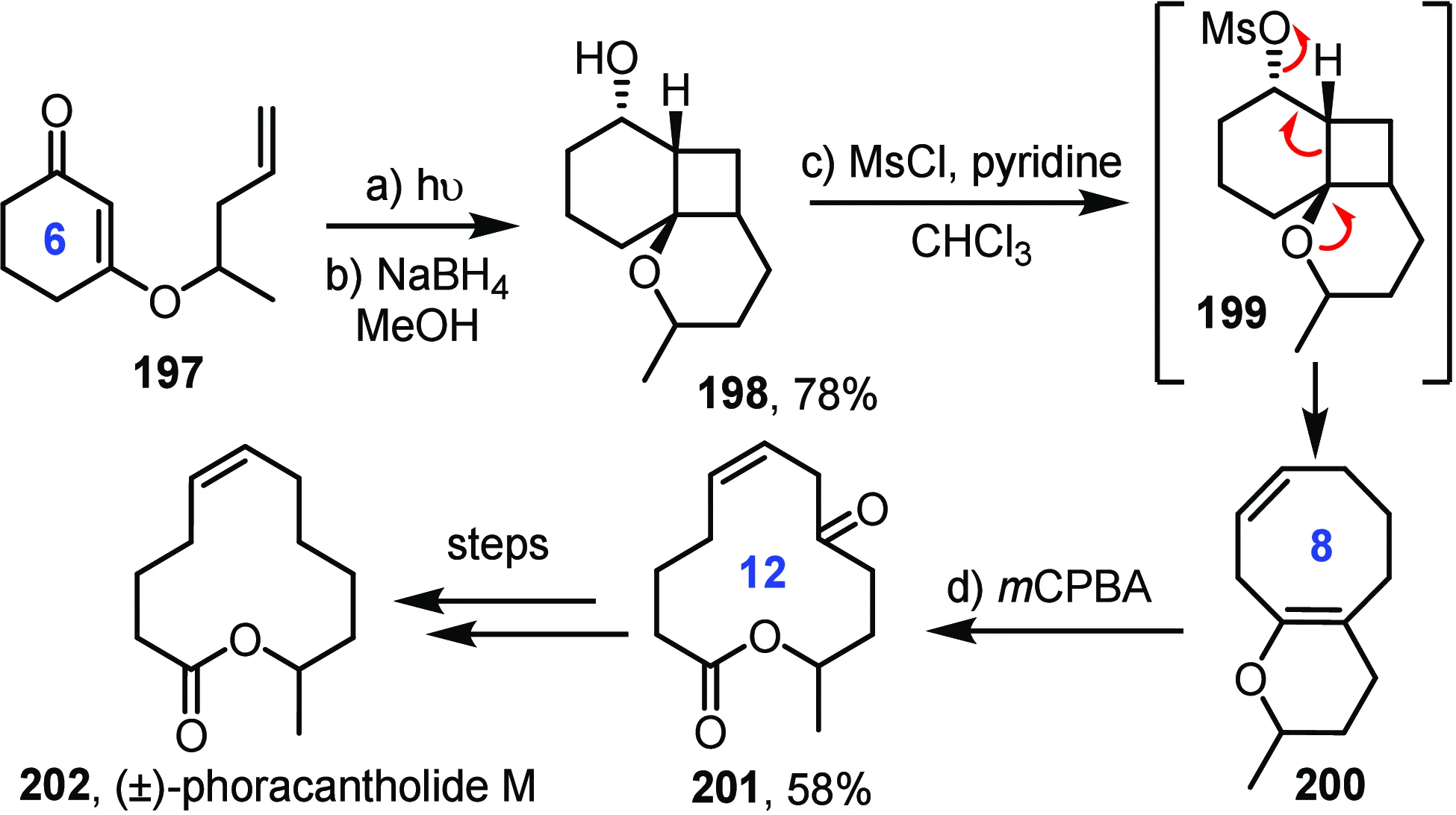
Grob/Oxidative Fragmentation Sequence

Vedejs and Reid^125^ used two different sulfur-mediated ring expansion reactions as part of their total synthesis of the carbocyclic cytochalasin natural products (Scheme 31). Thus, iodide **203** was heated in acetonitrile/K_2_CO_3_ to form sulfonium ylide **204**, which spontaneously underwent a 2,3-sigmatropic rearrangement to form **205**. Then, a second ring expansion reaction was performed via methylation of sulfide **205** using Meerwein’s salt followed by treatment with Zn/acetic acid to cleave one of the C–S bonds and furnish ring-expanded product **207**. This was then converted into the final target molecule cytochalasin D (**208**) via three additional steps.

**Scheme 31:**
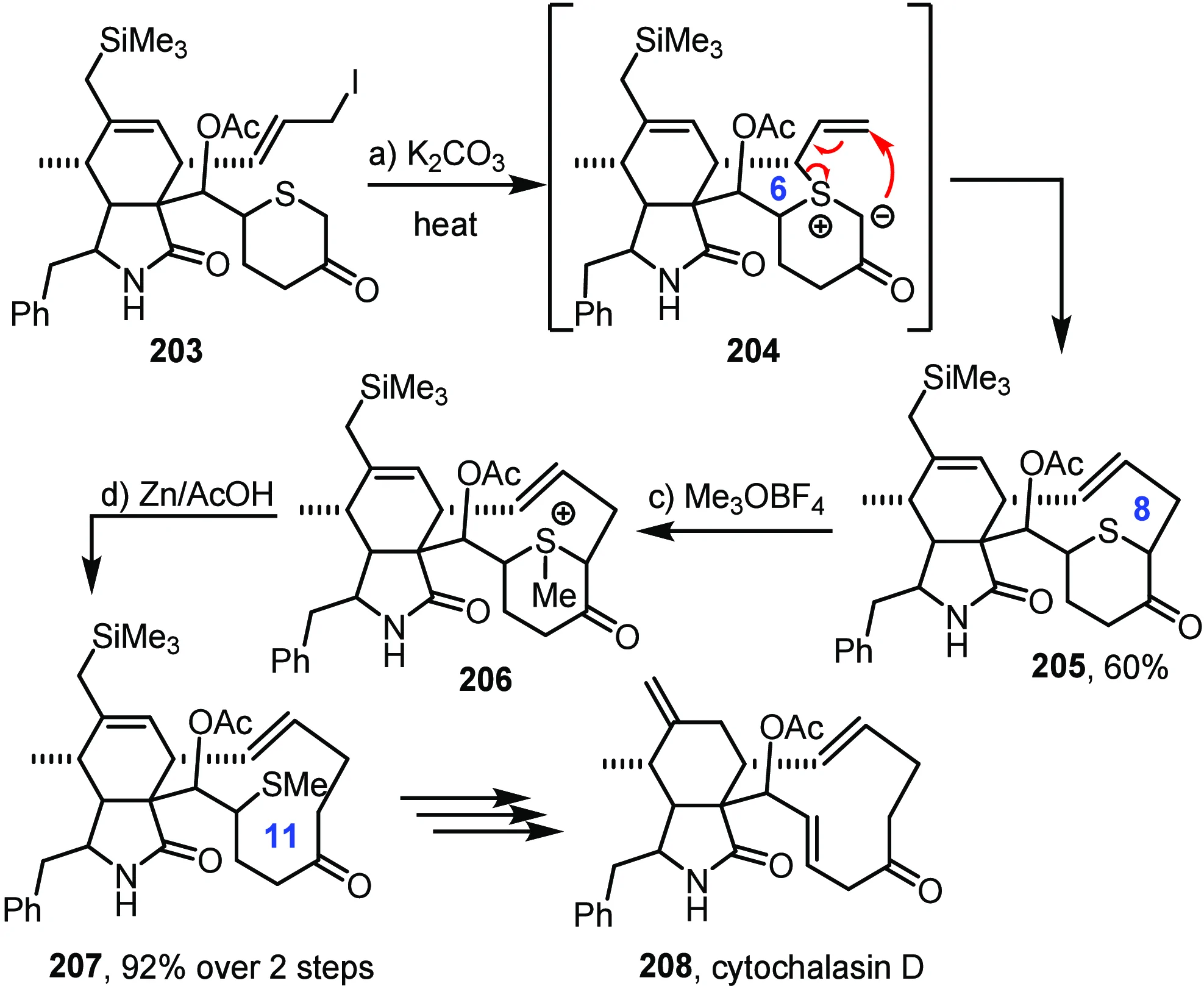
Consecutive Sulfur-Mediated Ring Expansion Reactions in the Total Synthesis of Carbocyclic Cytochalasin D

Dowd and Choi used consecutive ring expansions to convert cyclododecanone **209** into 15-membered macrocycle **216** (Scheme 32).^126^ The sequence started with a two-atom ring expansion method described by Huebner and co-workers.^127^ Thus, the reaction of enamine **210** and methyl propiolate affords cyclobutene intermediate **211**, which undergoes a thermally driven ring expansion to form 14-membered product **213**. Subsequent hydrogenation and alkylation then afforded alkyl iodide **214**. This substrate is set up to undergo one-atom ring expansion via tributyltin hydride-mediated halogen atom abstraction, cyclization, fragmentation and hydrogen atom transfer to afford the 15-membered-ring product **216**. This type of ring expansion—commonly referred to as the Dowd–Beckwith reaction—represents one of the most important and well-used classes of ring expansion in organic synthesis.^6,128,129^ Other radical ring expansion reactions of this type are featured later.

**Scheme 32:**
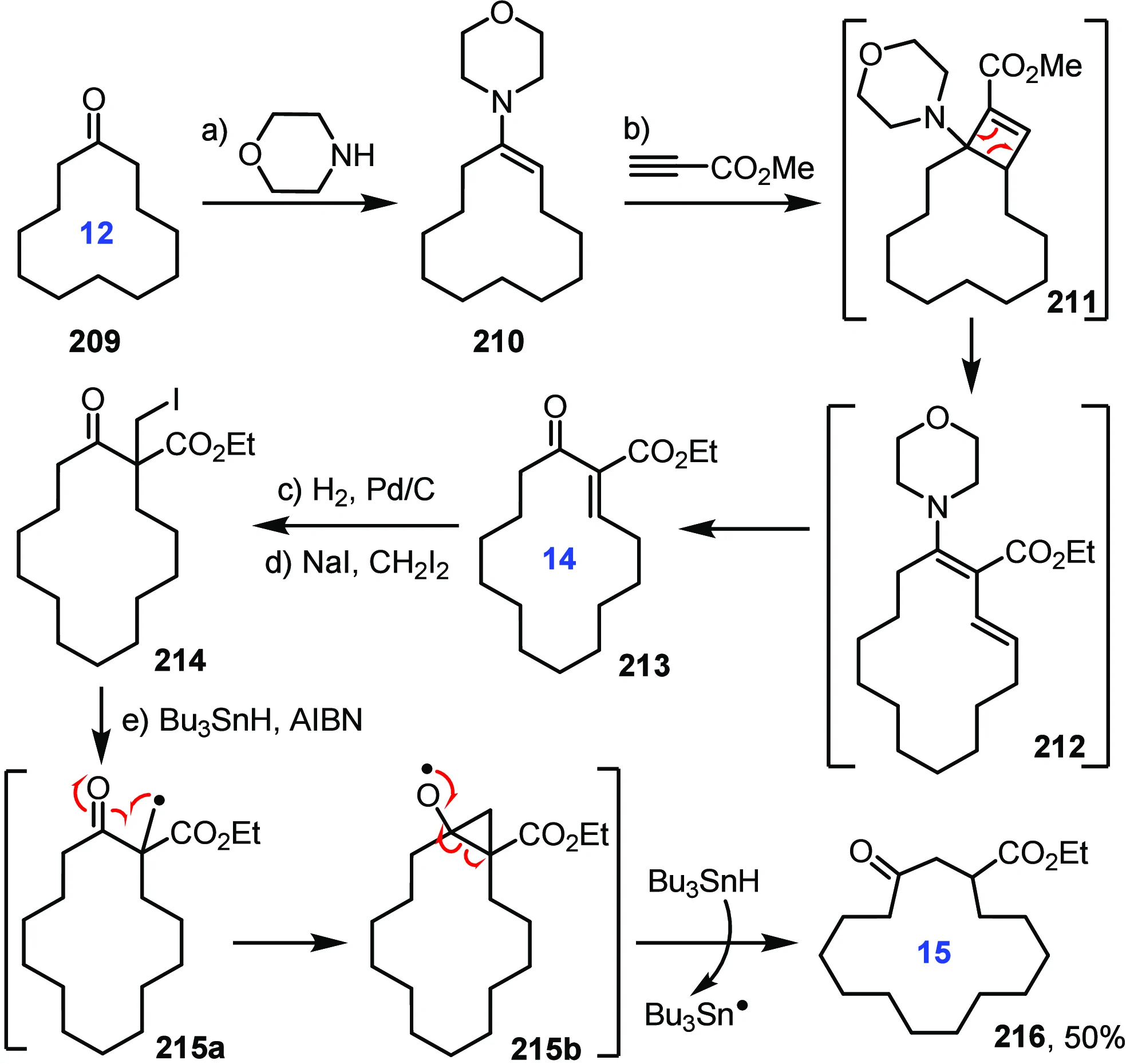
Consecutive Ring Expansions in the Synthesis of Macrocyclic Keto Ester **216**

Zhang and Dowd^130^ reported a double ring expansion process which involves both radical-mediated side chain insertion and Grob fragmentation (Scheme 33). In this study, cyclobutanone **217** is reacted under classical Dowd–Beckwith reaction conditions using AIBN and Bu_3_SnH. Tributyltin radical-mediated halogen atom abstraction leads to the formation of primary radical **218a**. This reactive radical then reacts via cyclization and fragmentation (**218a** → **218b**) to complete the first ring expansion reaction, with release of strain in the cyclobutyl ring, and formation of a more stable tertiary radical intermediate, both likely to help provide a driving force for ring expansion. From **218b**, H atom abstraction and dechlorination promoted by the tributyltin radical afforded the reduced cyclic ketone **219**. Then, lithium aluminum hydride reduction of the ketone, followed by mesylation and desilylation afforded compound **220**, which is set up to undergo a second ring expansion reaction, via a base-mediated Grob fragmentation, to form 11-membered-ring ketone **221**.

**Scheme 33:**
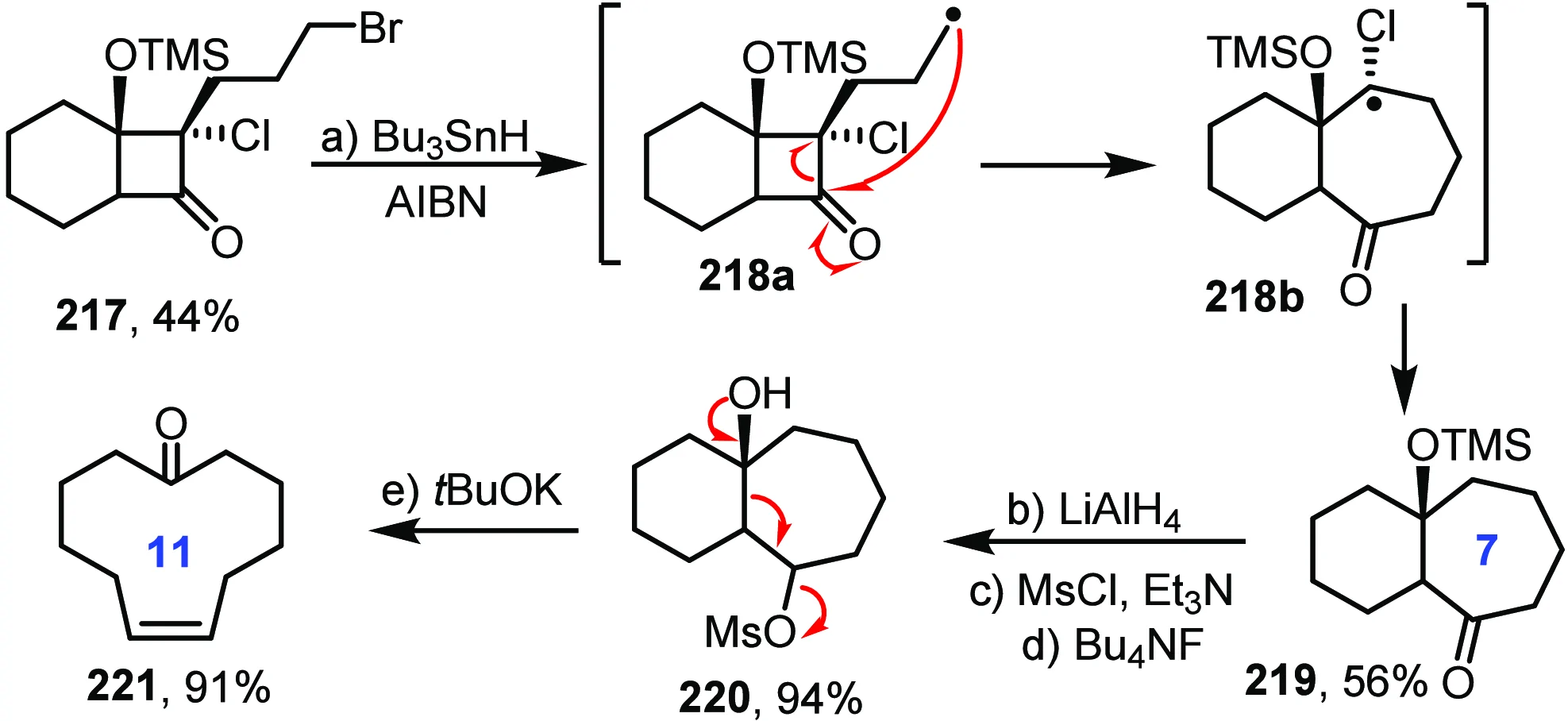
Consecutive Radical Ring Expansion and Grob Fragmentation Sequence

Thommen and co-workers^131^ developed an impressive approach to 15-membered ketones via consecutive Grob fragmentations (Scheme 34). Starting from tricyclic diol **222**, tosylation using *n*-butyllithium afforded **223**, and the first Grob fragmentation was performed upon treatment with potassium *tert*-butoxide to furnish 11-membered-ring product **224** as the major product. Then, lithium aluminum hydride reduction of **224** followed by tosylation afforded **225**, to set up a second Grob fragmentation, again promoted by potassium *tert*-butoxide, to form 15-membered-ring dienone **226** in high yield.

**Scheme 34:**
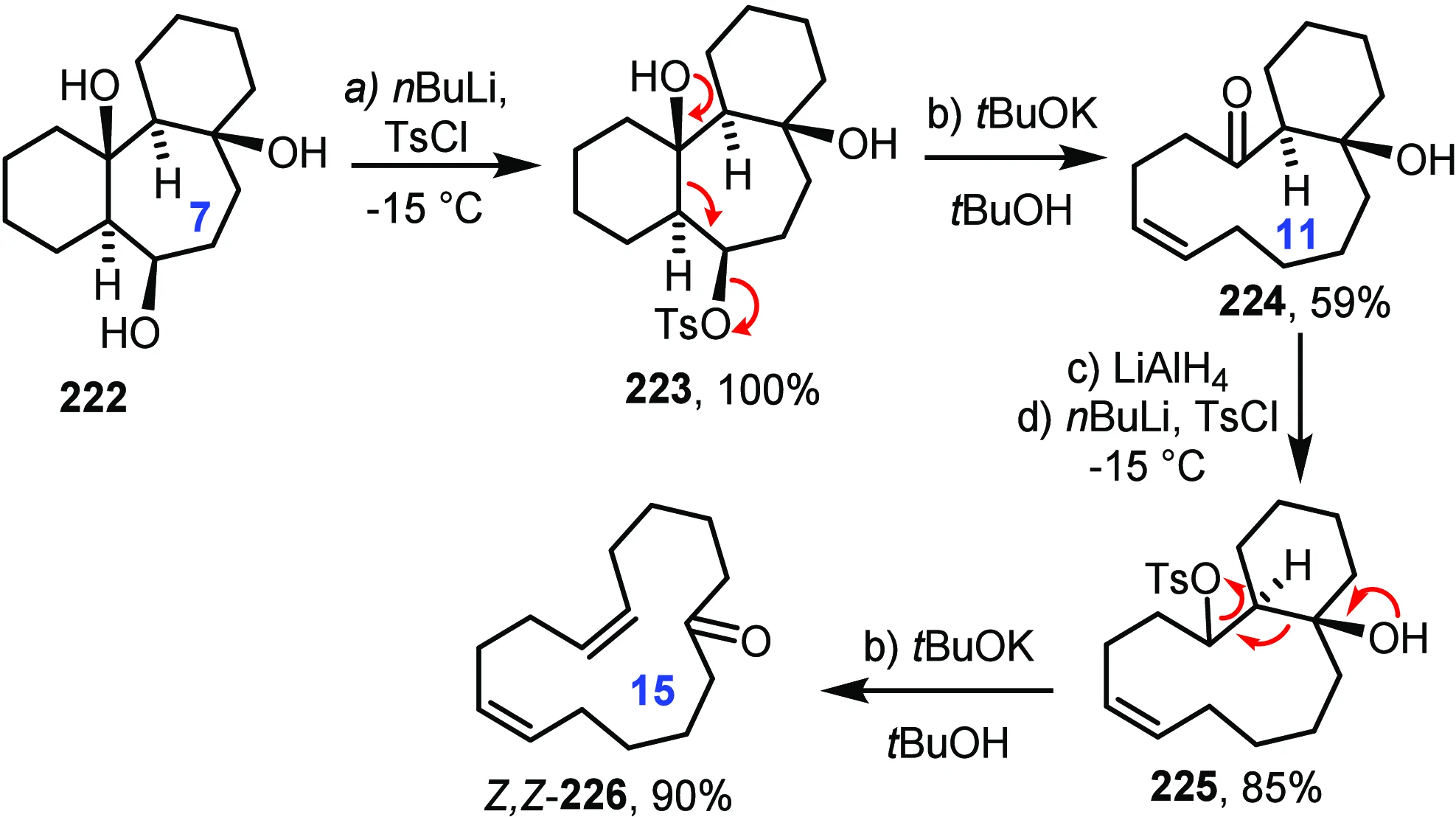
Consecutive Grob Fragmentation Sequence

Posner and co-workers^132^ reported a strategy whereby sequential ring expansions can be used to form medium-sized lactones, including the synthesis of natural product (−)-phoracantholide J (Scheme 35A). In this report, silyl enol ether **227** was treated with methyllithium to form the corresponding enolate, and then trapped with epoxide in the presence of boron trifluoride to form bicyclic hemiketal **229**. This was followed by oxidative fragmentation using diacetoxyiodobenzene and iodine, to form eight-membered-ring lactone **230**. Treatment with TBAF revealed primary alcohol **231** and this set up the second ring expansion reaction, which occurred via a spontaneous translactonization to form hydroxy lactone **232**; the thermodynamically favorable change in ring size (8- to 10-membered) likely facilitated this facile ring expansion. Radical deoxygenation was then used to complete the synthesis of (−)-phoracantholide J (not shown).

**Scheme 35:**
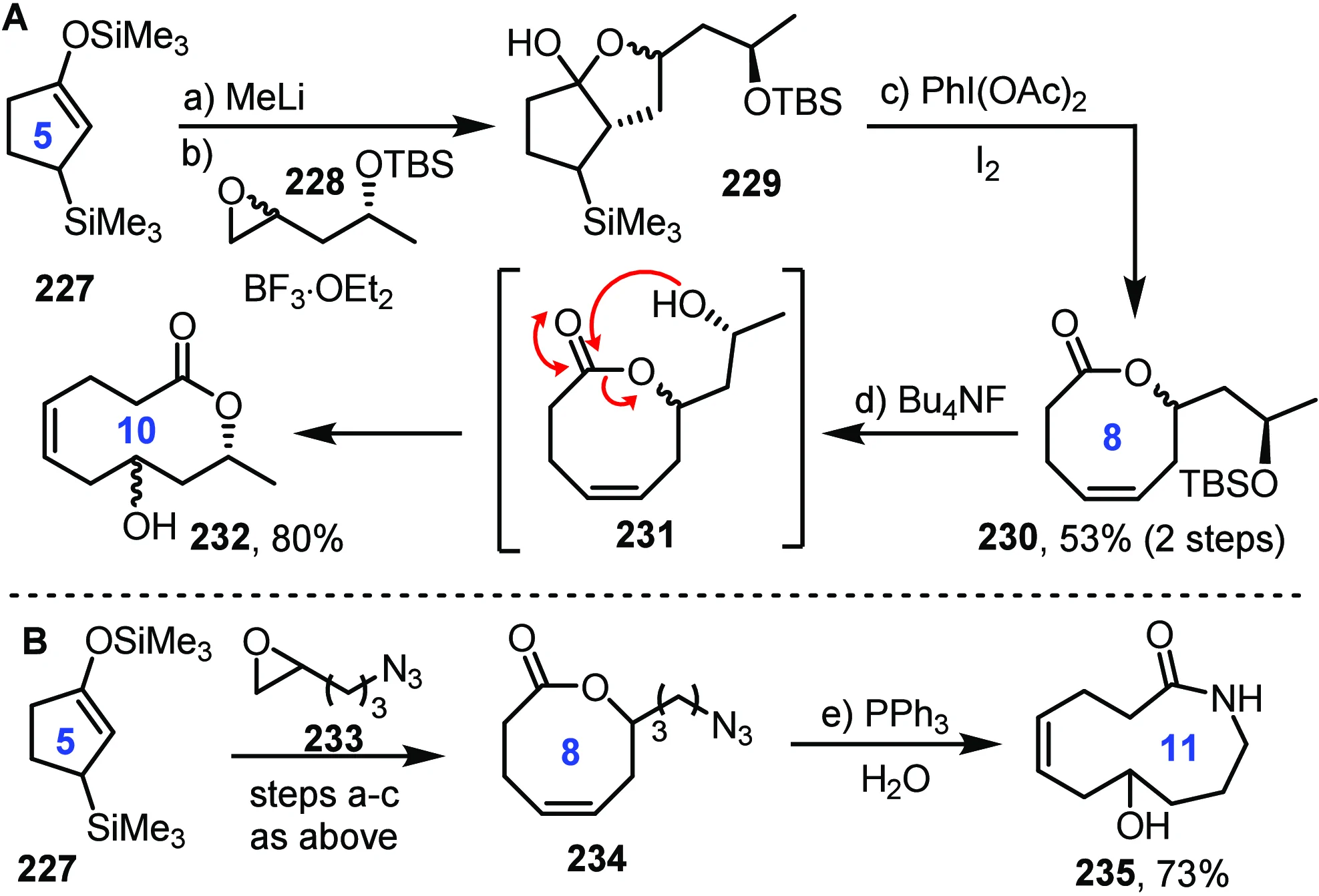
Consecutive Ring Expansions during the Total Synthesis of (−)-Phoracantholide J and Lactam **235**

The same team later went on to report a related sequence to make 11-membered-ring lactam **235**.^133^ In this study, silyl enol ether **227** was reacted in much the same way as in their previous work, but using azide-tethered epoxide **233**, to afford lactone **234** after three steps. Notably, this synthesis was performed on gram scale, demonstrating the scalability of the sequence. Then, Staudinger reduction of the azide triggered an 8- to 11-membered ring expansion to form **235**, presumably driven by the relief of transannular ring strain and formation of a strong amide bond (Scheme 35B).

Liu and Yeung developed an approach for sequential ring expansion via DBH-mediated semipinacol rearrangement, followed by a classical Dowd–Beckwith one-atom ring expansion.^134^ The sequence was used to synthesize a range of medium-sized-ring and macrocyclic cyclic ketones, typified by the overall three-step conversion of cyclotridecanone **236** into the natural product muscone (**239**), an important ingredient in perfumery (Scheme 36).The same team also reported that a third ring expansion step can be added to the sequence; this was demonstrated in the stepwise conversion of 12-membered **209** into 13-membered **240** via a semipinacol reaction, the conversion of **240** into 14-membered **241** via a Dowd–Beckwith reaction, and a second semipinacol reaction to form 15-membered-ring product **242**.

**Scheme 36:**
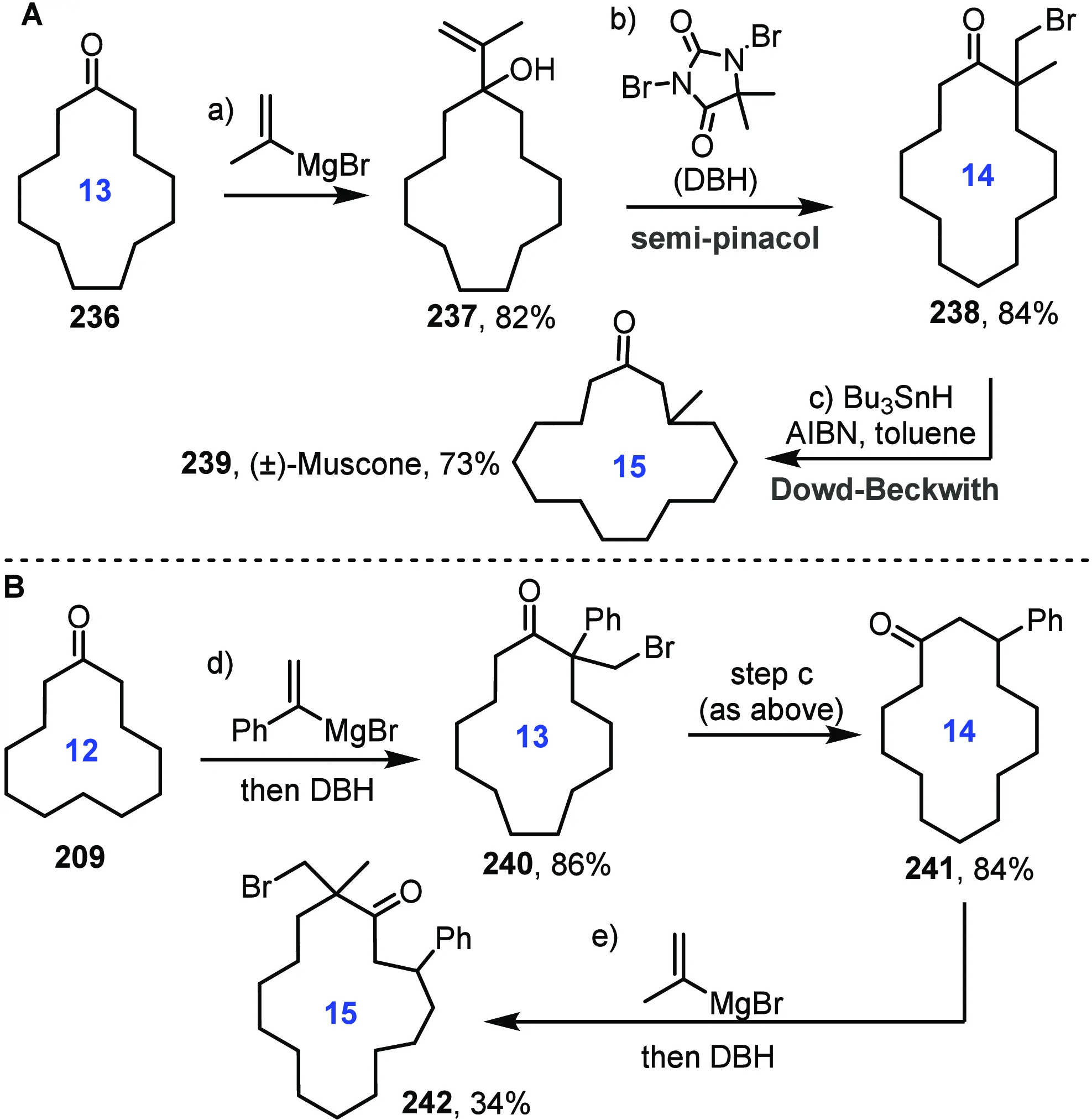
Sequential Semipinacol and Dowd–Beckwith Ring Expansion Reactions

Clayden and co-workers reported a method to access medium-sized rings via a three-atom ring expansion of lithiated ureas.^135,136^ In some cases, this was followed by a second ring expansion, via an acid-catalyzed fragmentation reaction (Scheme 37). The sequence starts with the synthesis of urea **244** from indoline **243**. Next, reaction with LDA and DMPU (to form carbanion **245**) promoted a migratory ring expansion to form ring-expanded urea **246**. The ring expansion reaction is likely to be driven by an increase in anion stability during rearrangement. The transformation is stereospecific and is proposed to operate via a concerted associative mechanism, rather than a stepwise S_N_Ar type process. Urea **246** can then undergo ring expansion for a second time, following treatment with *p*-TSA, to form 11-membered **248** an acid catalyzed fragmentation, as shown in Scheme 37.

**Scheme 37:**
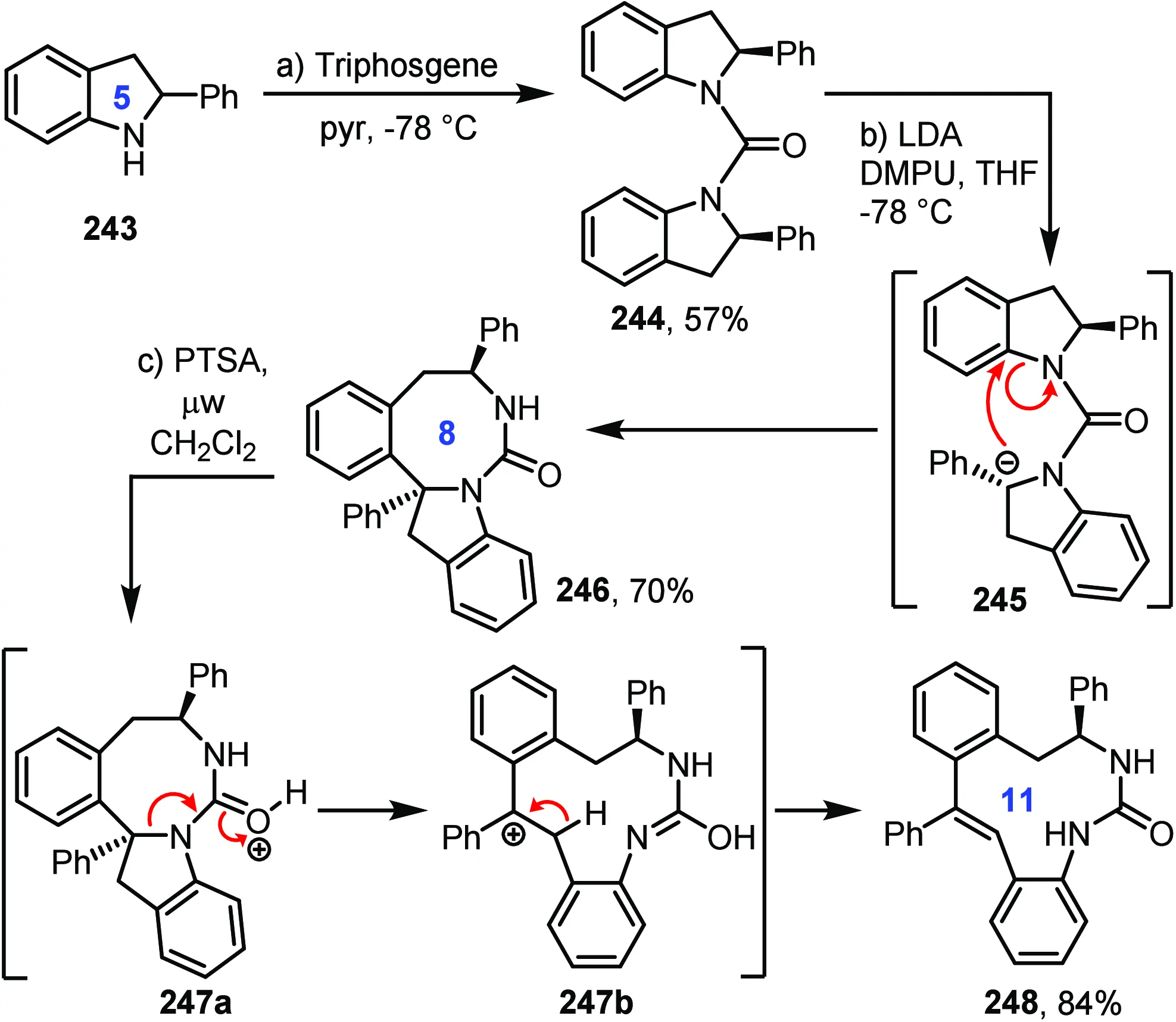
Consecutive Ring Expansion of Urea **245**

Dong and co-workers reported a consecutive ring expansion method for the conversion of indanone **249** into eight-membered-ring lactam **253** (Scheme 38).^137^ This study was primarily focused on the development of a rhodium-catalyzed direct insertion of ethylene into 1-indanones. The reaction involved oxidative addition (to form **250**), 2π insertion to give an enlarged metallocycle (**251**), and C–C reductive elimination to enable an overall novel two-carbon ring expansion strategy to form seven-membered cyclic ketones **252**. This “cut-and-sew” type approach is notable for operating on relatively unstrained 1-indanone starting materials; previous methods of this type typically rely on more strained three- and four-membered-ring systems.^138^ In a single case, one of the seven-membered cyclic ketone products formed in this way (**252**) was further expanded to eight-membered-ring lactam **253**, following reaction with sodium azide under acidic conditions.

**Scheme 38:**
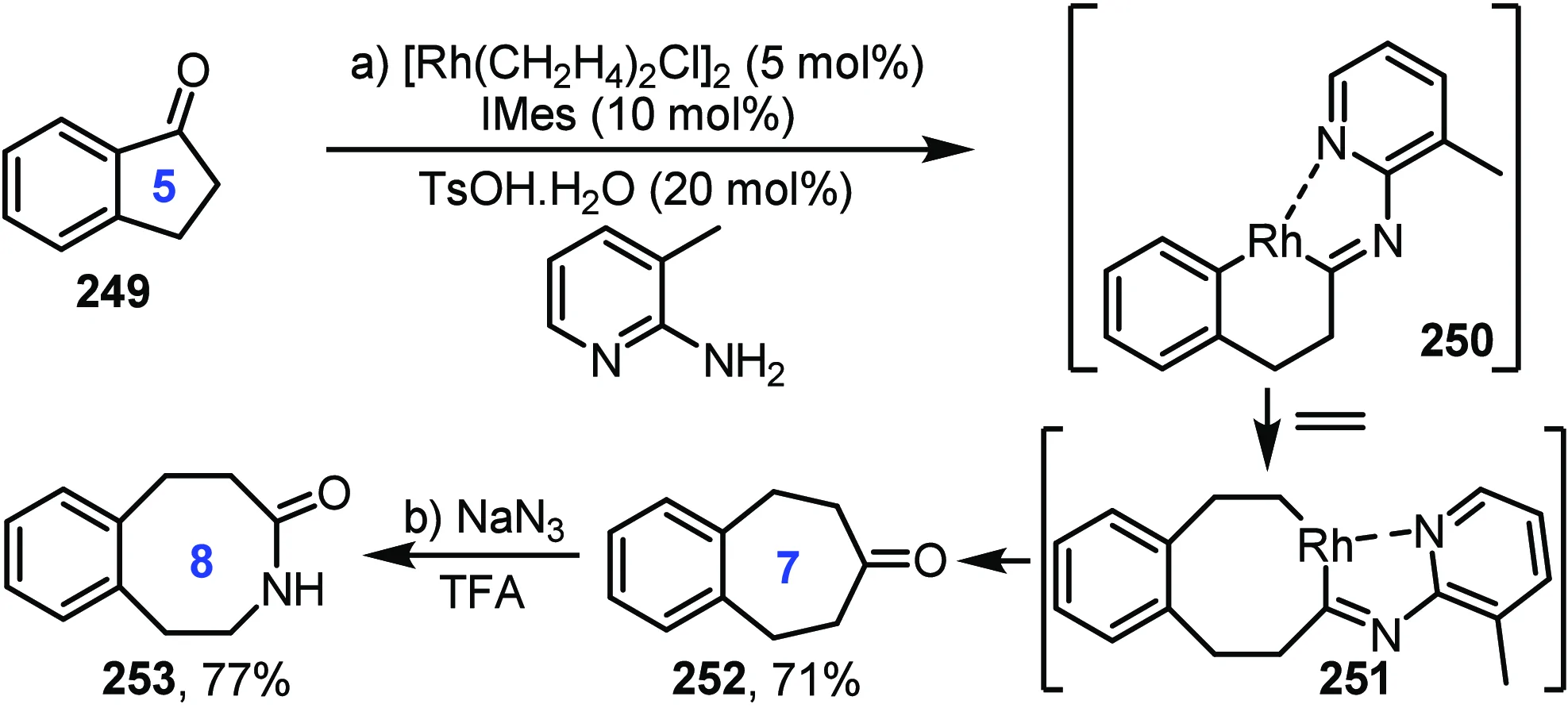
Consecutive Ring Expansion of Indanone **249**

Wang and co-workers reported an elegant consecutive ring expansion strategy for the total synthesis of marine alkaloids motuporamines A and B (**255** and **258**, Scheme 39A,B).^139^ Each synthesis involves a cyclic β-keto ester ring expansion of the type described earlier in the review and summarized in Scheme 20. Starting from cyclooctenone **150a**, a Tiffeneau–Demjanov-type one-atom ring expansion homologation was used to form nine-membered-ring β-keto ester **151b**. Acylation with acid chloride **129** and Fmoc cleavage followed, enabling spontaneous ring expansion to form 13-membered ring **254** in good yield, which in turn was converted into motuporamine A salt **255** by standard methods. In a similar way, enamine **256** was converted into 10-membered-ring β-keto ester **151c** using a two-atom ring expansion method reported by Brannock and co-workers (Scheme 39B).^140^ The same type of β-keto ester ring expansion was then used to form 14-membered ring **257**, followed by conversion into motuporamine B salt **258**. Ketone **150c**, a key intermediate within Scheme 39B, could also be used in a consecutive ring expansion sequence without activation via addition of the ethyl ester moiety (Scheme 39C). Here, use of a Beckmann rearrangement (notably, under different conditions to those previously used on the same compound in Scheme 23), enabled the one-atom ring expansion to lactam **152d**. Following this, conversion to 2-vinyl benzylamine **259** enabled an aza-Cope rearrangement analogous to those in Scheme 16, furnishing the desired 15-membered product **260** in 45% yield following careful optimization of reaction solvent.^141^

**Scheme 39:**
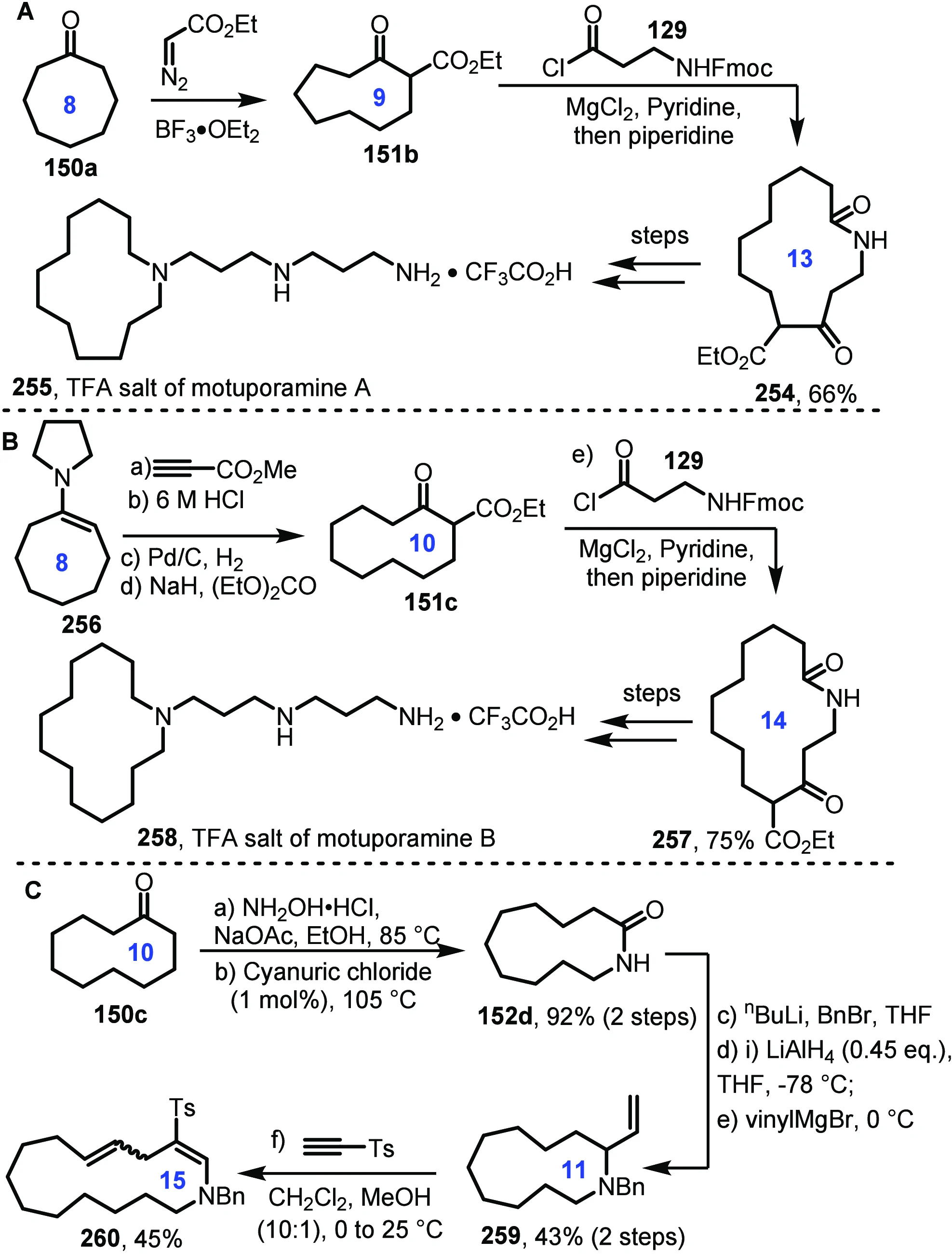
(A, B) Consecutive Ring Expansion in the Total Syntheses of (A) Motuporamine A and (B) Motuporamine B; (C) Consecutive Beckmann and Aza-Cope Reactions

Consecutive ring expansion strategies have been demonstrated to be a powerful tool in the synthesis of marine alkaloids (Scheme 40). Huang and co-workers^142^ used the aza-Claisen rearrangement of 2-vinyl amide **261** to give stereocontrolled access to 10-membered lactam **262**. This lactam then underwent functionalization to introduce a pendant amine moiety, forming compound **265**. Amine deprotonation then allowed ring expansion via a zip reaction to form 14-membered system **266**, which is likely driven by both reduction of transannular strain and the formation of a more stable nitrogen anion within the amide product. Simple amide reduction then allowed the formation of (+)-(*R*)-haliclorensin (**267**). The closely related 10-membered natural product isohaliclorensin (**264**) was also accessible via an adaptation of the same route.

**Scheme 40:**
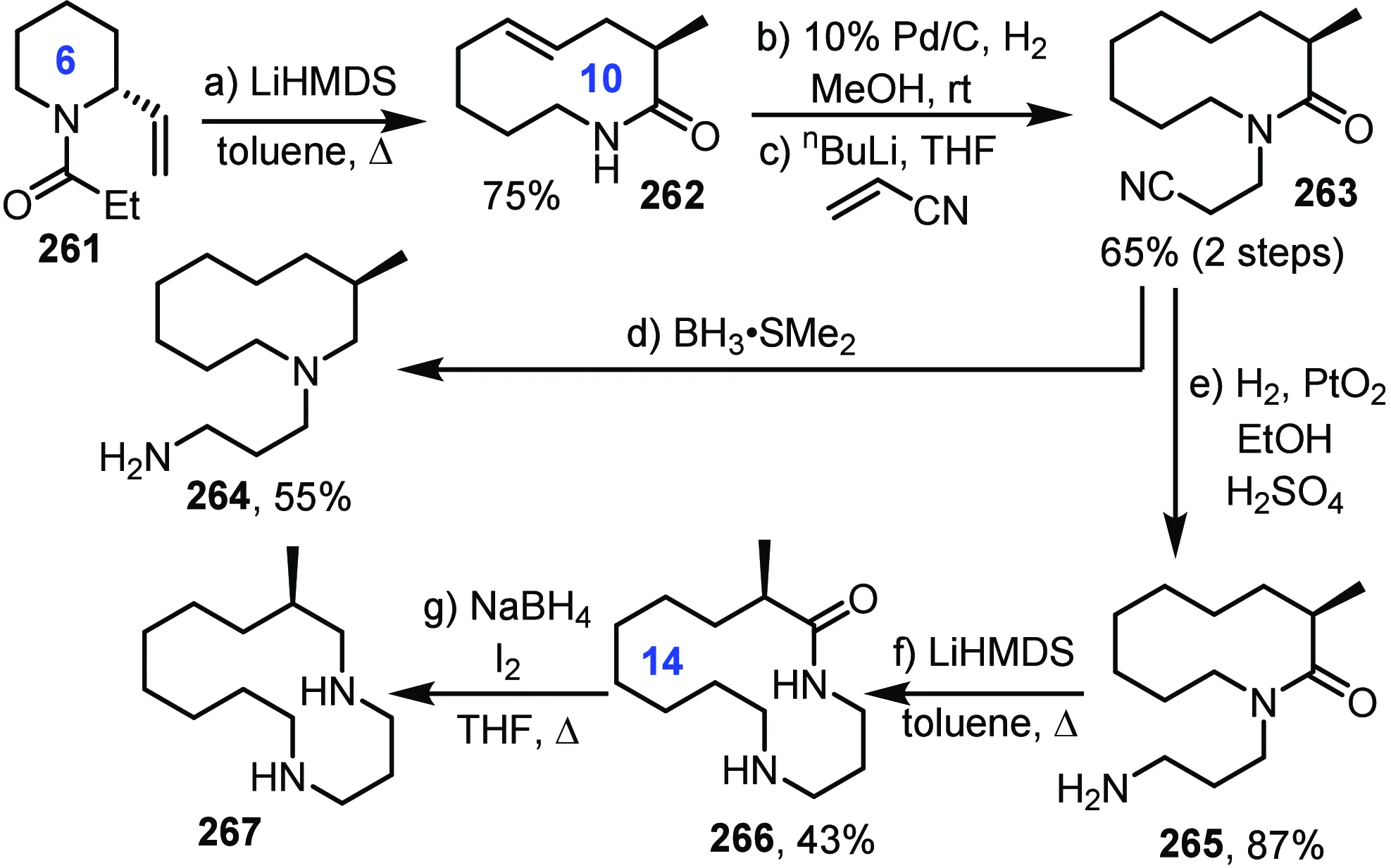
Use of Consecutive Ring Expansion Reactions in the Synthesis of the Haliclorensin Alkaloids

Harrowven and co-workers reported an original consecutive ring expansion sequence to form azocines and benzoazocines (Scheme 41).^143^ The medium-sized-ring formation step relies on a photochemically induced [1,3]-sigmatropic rearrangement, with several examples reported, typified by the ring expansion of six-membered **271** into **272** under UVA irradiation. The six-membered precursors were also prepared using ring expansion via a series of thermal rearrangement reactions starting from four-membered-ring precursors of the type **268**.^144^ The full sequence can be telescoped and performed under continuous flow conditions to afford the medium-sized-ring product **272** in an impressive 74% yield from **268**.

**Scheme 41:**
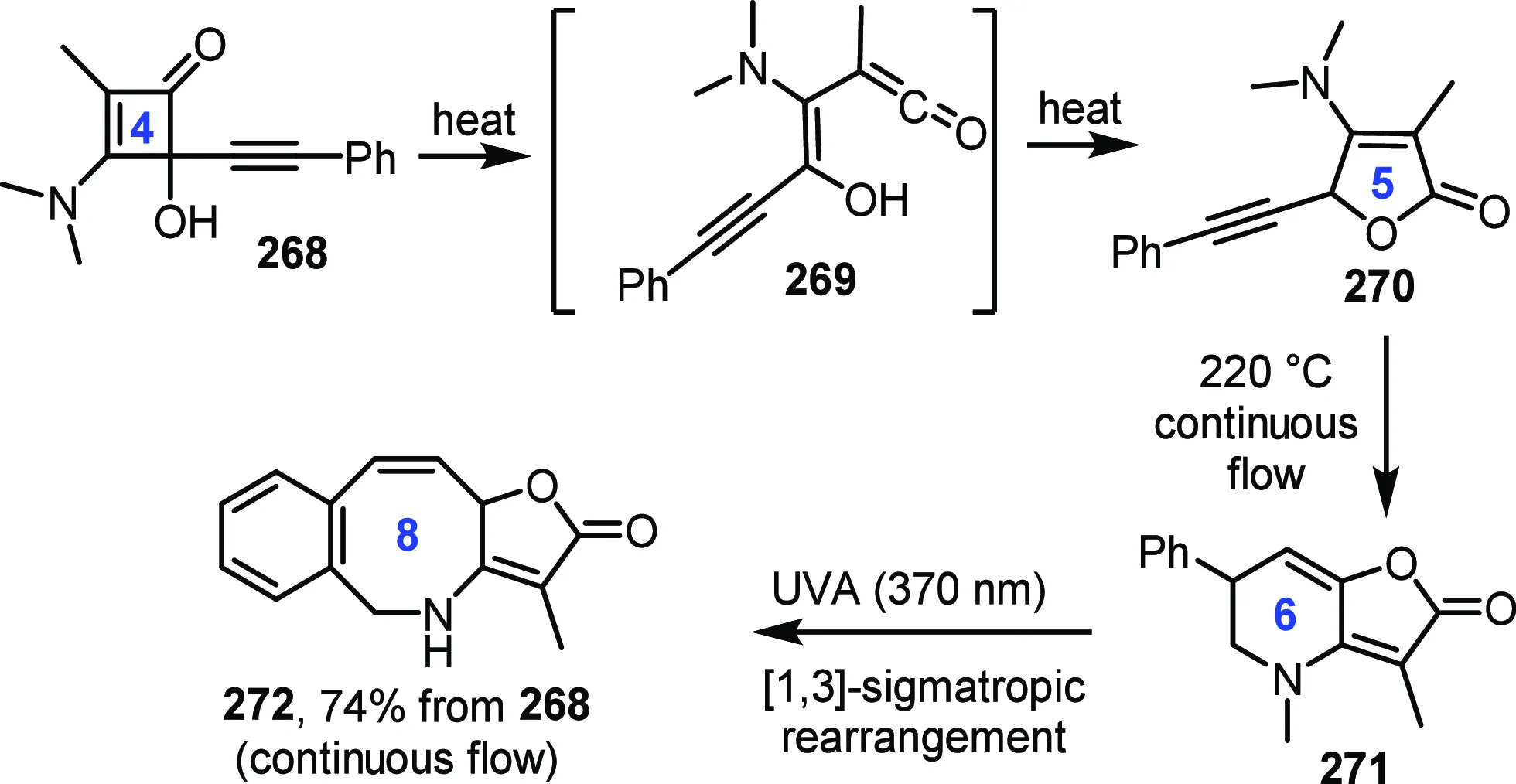
Consecutive Thermal and Photochemical Ring Expansion Reactions

An extension to the CARE methodology introduced earlier in the review (Scheme 24) was used as part of a consecutive ring expansion to macrocyclic lactones, starting from acryloyl imide **273** (Scheme 42A).^109^ The first ring expansion was performed by reacting imide **273** with 20 equiv of nitromethane and DBU in CH_2_Cl_2_ at RT; this promotes a conjugate addition (**273** → **274a**) followed by deprotonation and ring expansion (**274a** → **274b** → **275**) to form **275** in 89% yield. The nitroketone product **275** was then expanded further using a procedure adapted from a published method by Stach and Hesse,^145^ performed by reacting **275** with 1,4-benzoquinone and DBU. This promotes a conjugate addition and spontaneous ring expansion cascade reaction via a phenolate intermediate. Using this method, **275** was converted into 15-membered-ring lactone **277** in 75% yield. Macrocycles **278** and **279** were also prepared in comparable yields using the same approach (Scheme 42B).

**Scheme 42:**
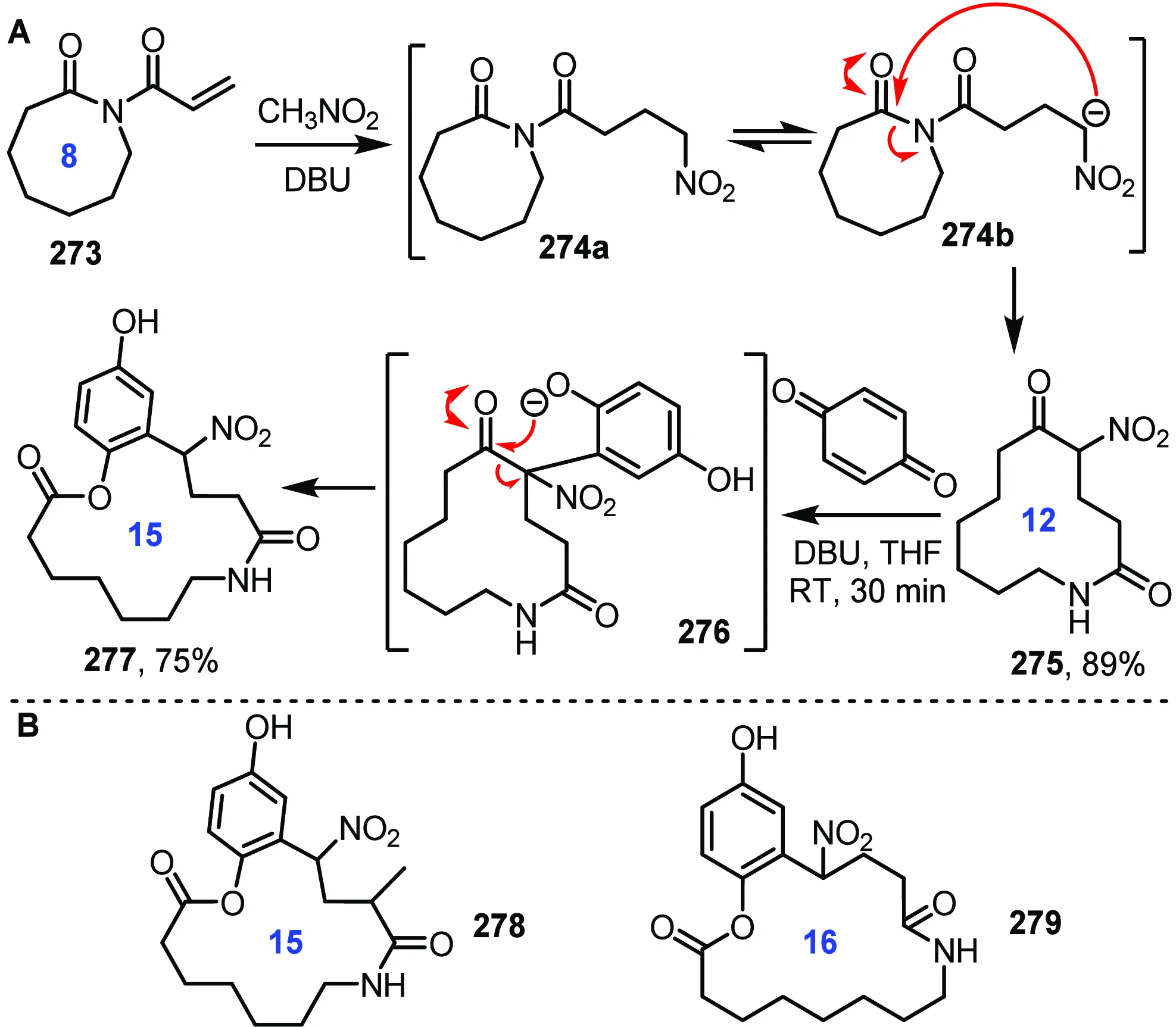
(A) Consecutive Cascade Ring Expansion Reactions for Macrolactone Synthesis; (B) Example Products

Additional extensions to the CARE approach have also been used to enable consecutive ring expansion methods based on C–N, C–C, and C–O bond formation (Scheme 43).^109^ For example, the CARE reaction of acryloyl imide **173** with 4-fluorobenzylamine and subsequent *N*-acylation with acryloyl chloride enables the formation of 10-membered-ring acryloyl imide **280** using the approach described earlier in the review (see Scheme 24). The divergent ring expansion of **280** was then demonstrated, enabling the formation of three different 14-membered-ring products **281**, **282**, and **283**; these reactions were done either by repeating the CARE reaction using 4-fluorobenzylamine (step a), the nitromethane CARE method described above (step b), or dihydroxylation of the alkene of **280** using AD-mix-β and subsequent ring expansion (similar to Scheme 22).

**Scheme 43:**
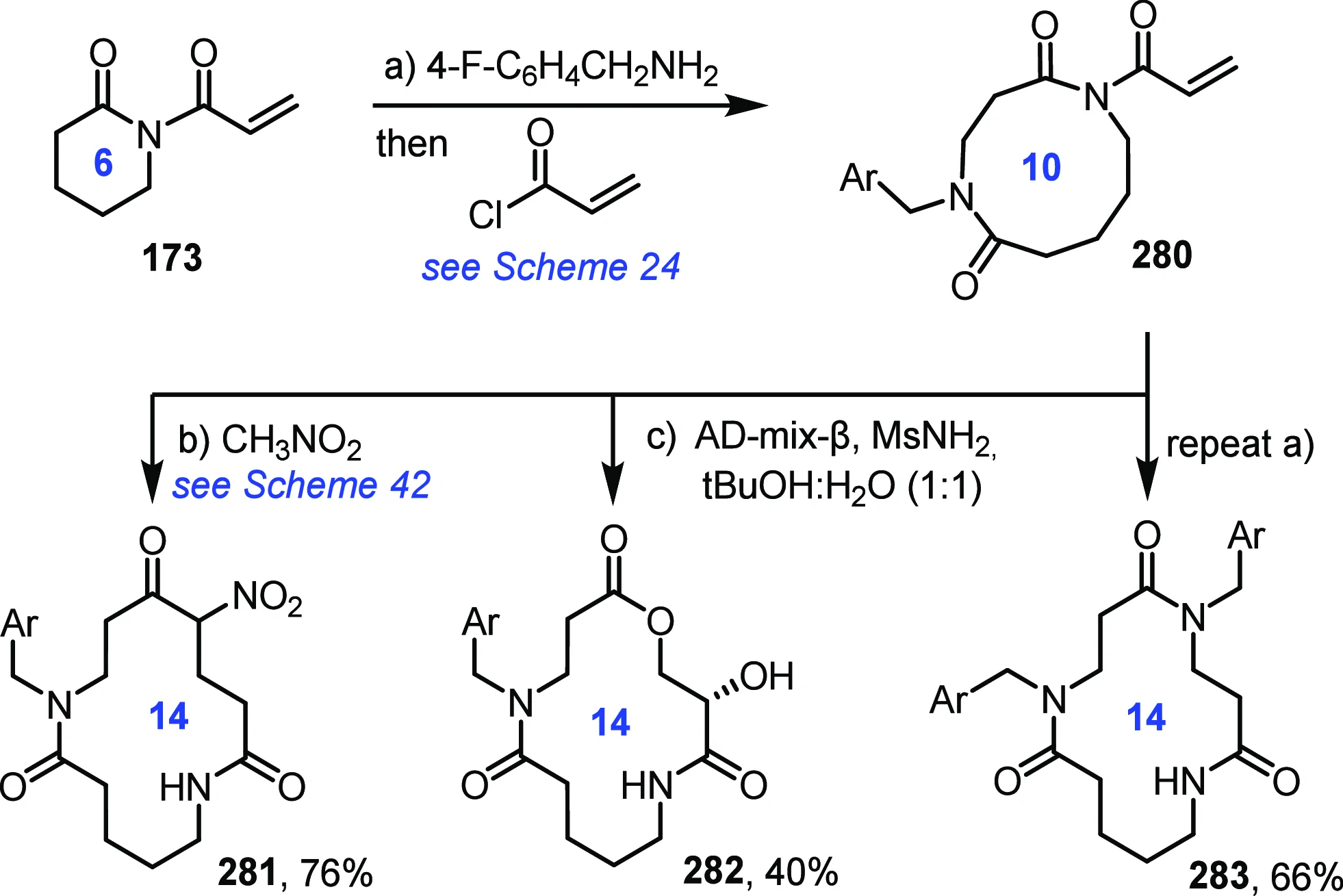
Consecutive Cascade Ring Expansion Reactions of Acryloyl Imides

Three consecutive ring expansion reactions were used during the synthesis of the macrocyclic framework of the natural product solomonamide (Scheme 44).^146^ This approach starts with the NaIO_4_-mediated oxidative ring expansion of commercially available tetrahydrocarbazole **284** to form nine-membered-ring lactam **285** in 98% yield using a method adapted from that of Dolby and Booth.^147^ This is then followed by two further three- and four-atom SuRE-type lactam ring expansion reactions of the type summarized earlier in Schemes 21 and 22. This sequence enabled the incorporation of various amino acid- and hydroxy acid-derived linear fragments into 15- to 17-membered ring-enlarged solomonamide-like macrocyclic frameworks (e.g., representative products **287**–**289**).

**Scheme 44:**
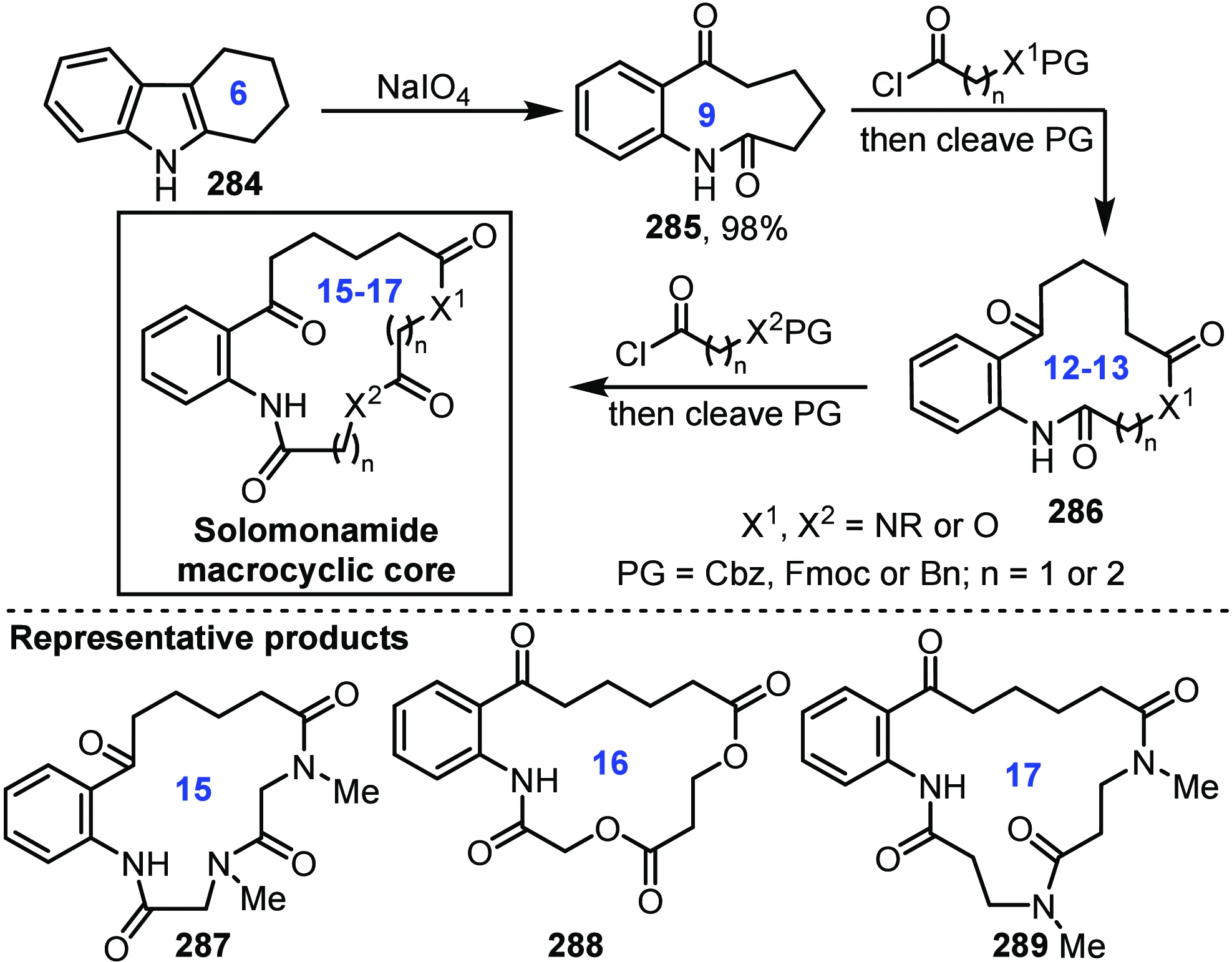
Consecutive Ring Expansion Reactions in the Synthesis of the Solomonamide Framework

De Paolis and co-workers reported an ingenious consecutive ring expansion strategy to prepare macrocycle **295** (Scheme 45).^148^ First, cyclic diketone **290** was reacted with organocatalyst **291** in a three-atom ring expansion to form nine-membered-ring lactone **293**; the product is formed with high *er* and *dr*, with the organocatalyst enabling efficient desymmetrization of the prochiral starting material and epimerization to form *trans-* **293** as the major diastereoisomer. Then, zinc-mediated nitro reduction enabled the conversion of **293** into indole **294**, which was then reacted with NaIO_4_ to effect oxidative cleavage of the annulated indole and form 12-membered-ring product **295**.

**Scheme 45:**
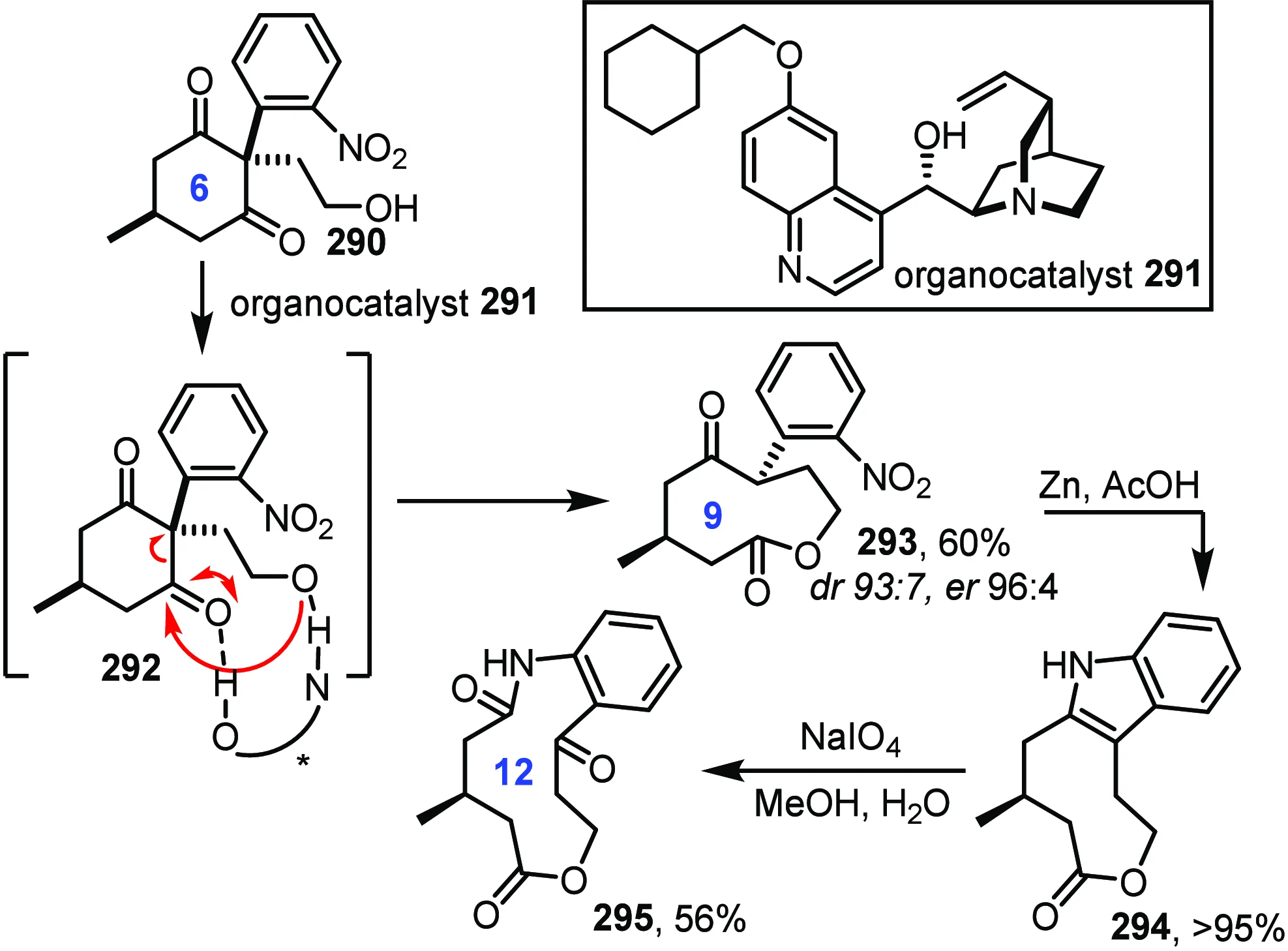
Consecutive Ring Expansion of 1,3-Cyclohexanedione **290**

Knowles and co-workers reported the use of consecutive ring expansions to convert simple norbornene system **296** into tricyclic lactam **300** (Scheme 46). First, a one-atom ring expansion with concomitant bridging annulation was performed using an oxidative cleavage/double Mannich reaction sequence to form ketone **298** with high levels of stereocontrol, overall converting a five-membered ring into a six-membered ring. The ester moiety then underwent a one-pot deprotection/activation sequence to form acyl ammonium intermediate **299**, where ring strain drove the fragmentation to form the eight-membered-ring system **300**.^149^

**Scheme 46:**
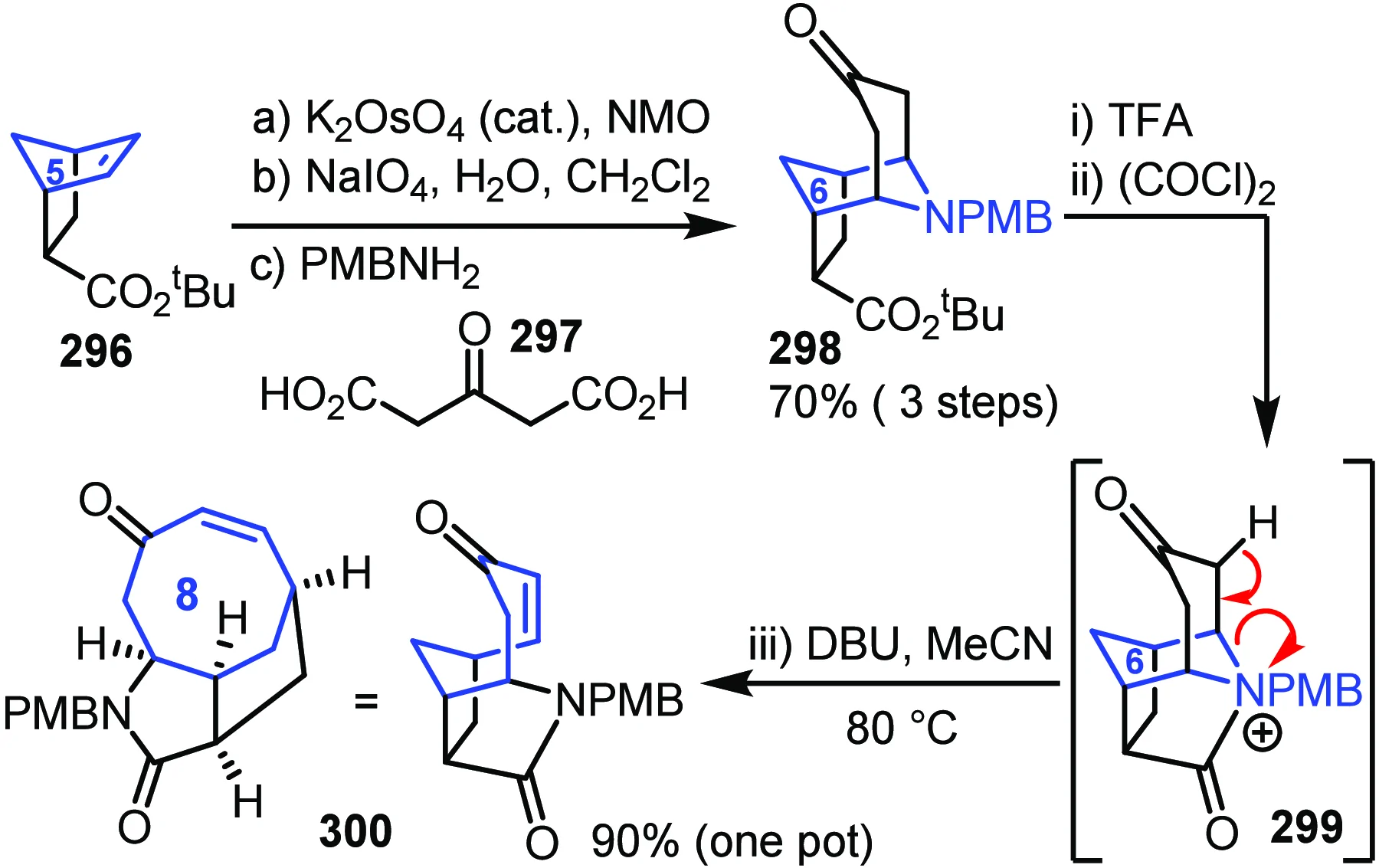
Consecutive Ring Expansions in the Synthesis of Tricyclic Lactam **300**

A novel consecutive ring expansion was recently reported based on the application of two distinct classes cascade ring expansion methods described earlier in the review (see Schemes 9–11 and 24). Together, these reactions were combined to form a general, modular approach for the synthesis of macrocyclic sulfonamides (Scheme 47).^150^ The sequence starts from easy to prepare linear substrates of the form **301**, which contain a terminal carboxylic acid group, an internal tertiary amine and a terminal vinyl sulfonamide. The first cascade ring expansion (**301** → **302a** → **302b**) is then initiated via activation of the carboxylic acid using T3P, with the vinyl sulfonamide group of **301** acting as a terminal nucleophile for the cascade, to form medium-sized sulfonamides **302b**. These products tended to be relatively unstable and hence were typically reacted directly in a second cascade ring expansion via a CARE approach to form macrocyclic sulfonamides **304**. The macrocyclic final products were found to be much more stable than the intermediate medium-sized rings, and this approach was validated through the synthesis of 42 diversely functionalized 13- and 14-membered macrocyclic sulfonamides **304**.

**Scheme 47:**
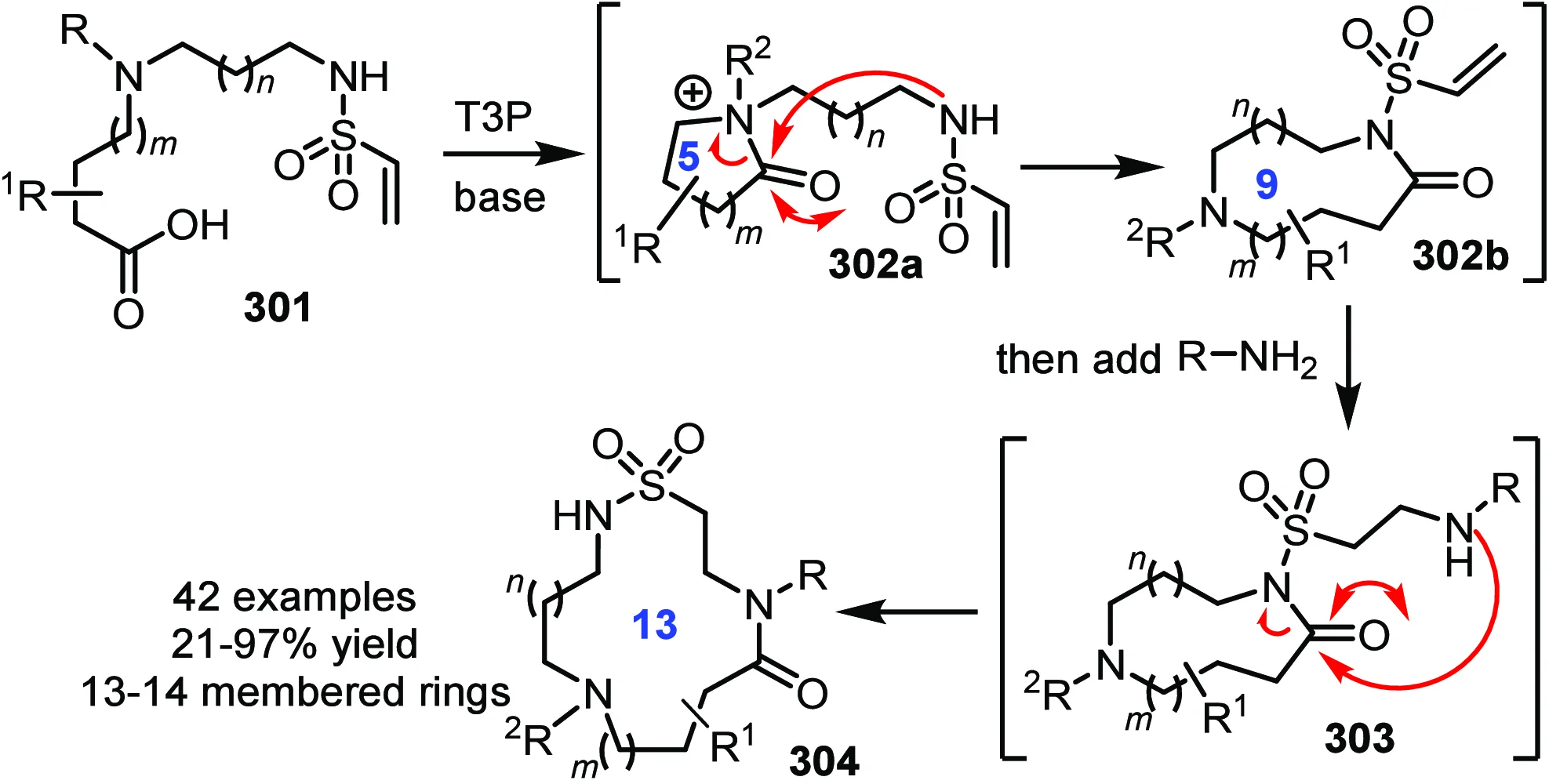
Consecutive Cascade Ring Expansion Reactions for Macrocyclic Sulfonamide Synthesis

In recent work, the same two classes of ring expansion were also combined in another consecutive ring expansion sequence to synthesize the proposed structure of the 13-membered-ring macrocyclic alkaloid celacarfurine (**309**) (Scheme 48).^151^ Following the synthesis of linear precursor **306**, reaction with T3P promoted a cascade ring expansion of the type described earlier in Scheme 9, to form nine-membered-ring lactam **307**. Then, a SuRE reaction followed to convert **307** into 13-membered macrocycle **308**. Protecting group cleavage and *N*-acylation completed the synthesis of proposed structure of celacarfurine **309**, although the data for synthetic sample did not match those of the isolated material.^152^ In addition to **309**, a series of 12- and 13-membered analogues were also produced, with X-ray crystallographic data supporting the formation of the macrocyclic scaffold in several cases. Interestingly, one the oldest consecutive ring expansion sequences in this section (Wasserman and co-workers’ 1980 study, see Scheme 29)^120^ and this most recent example were both used to target 13-membered macrocyclic alkaloids in the spermidine family.

**Scheme 48:**
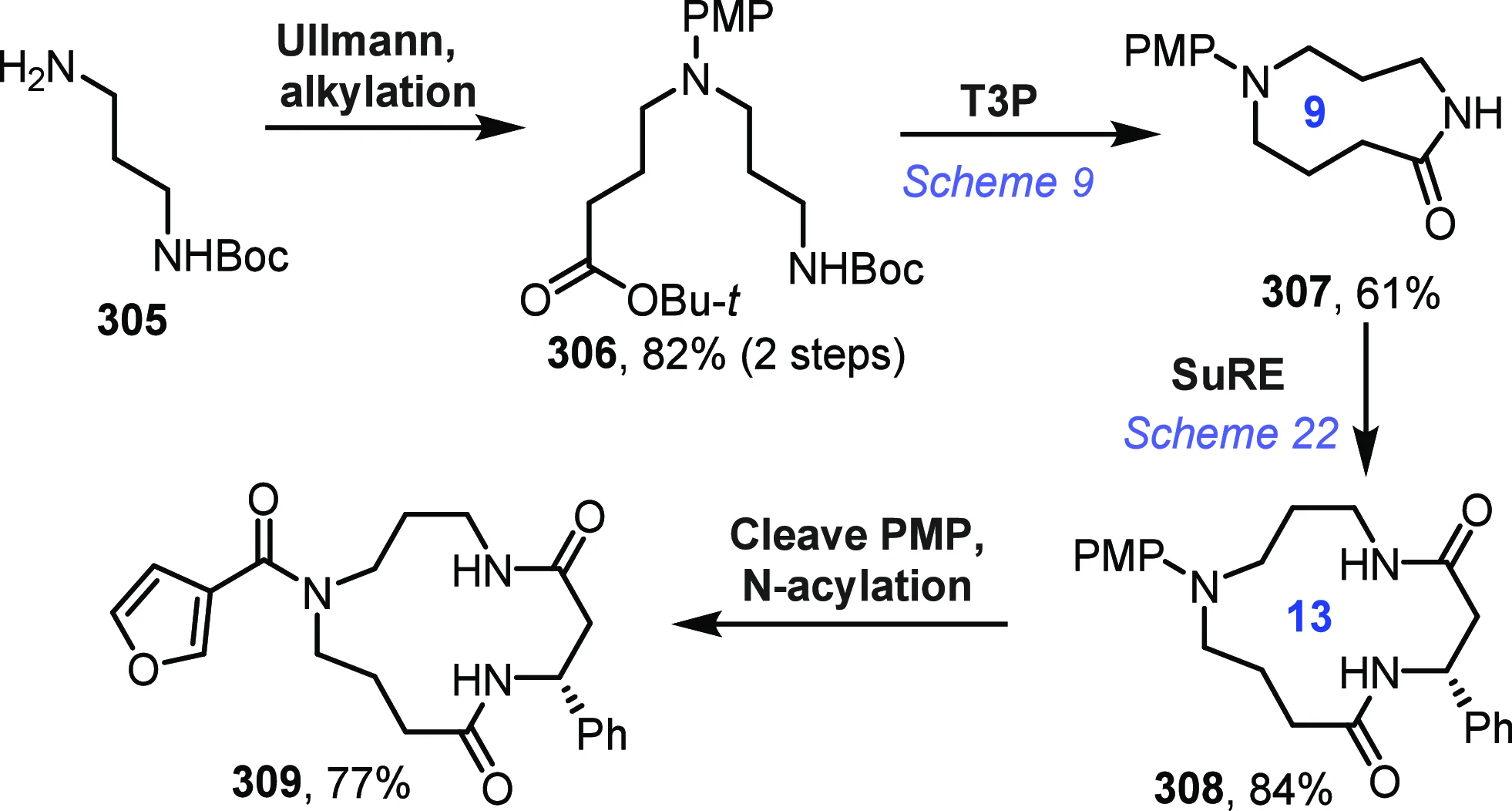
Consecutive Ring Expansion Strategy for the Synthesis of the Proposed Structure of Celacarfurine

## 5 Conclusion and Perspective

As our toolbox of ring expansion methods grows, so does our ability to design syntheses of increasingly complex macrocyclic products. This enables new cyclic products to be generated via nonobvious retrosynthetic disconnections based on ring growth, e.g., via cascade reactions of the type described in section 2. The iterative ring expansion methods described in section 3 are also important in this regard. The ability to “grow” macrocycles via the controlled insertion of linear fragments offers a fundamentally different strategy compared with classical end-to-end cyclization of a long linear precursor. The consecutive ring expansion methods in section 4 expand this concept to different reaction types, and arguably have the greatest potential, given the increased range of products accessible by combining different ring expansion modes.

Highlighting the power of ring expansion methods to improve the efficiency of large ring synthesis was one of the key factors that motivated the writing of this review. In addition, another major driver for bringing this material together was to help identify future opportunities for researchers seeking to apply successive ring expansion strategies. By considering the general approaches discussed in this review, it is easy to imagine scenarios in which methods where a single ring expansion step has been demonstrated can be adapted to enable iterative ring expansion. For example, Chen and co-workers^153^ developed an impressive ring expansion sequence based on a ruthenium-catalyzed C–C bond formation/retro-aza-Michael addition/lactamization sequence (e.g., **310** → **312**) using glycine-derived azlactone **311** as the two-carbon synthon. Subsequent stepwise global reduction (e.g., **312** → **313** → **314**) allowed the overall conversion of five- and six-membered cyclic amines into their two-carbon-expanded homologues (Scheme 49). Although not reported in Chen’s study, reapplication of the same sequence to eight-membered-ring product **314** should allow the sequence to be performed iteratively, e.g., to form 10-membered ring **315**, and thus enable a five-step approach to perform iterative two-carbon homologations.

**Scheme 49:**
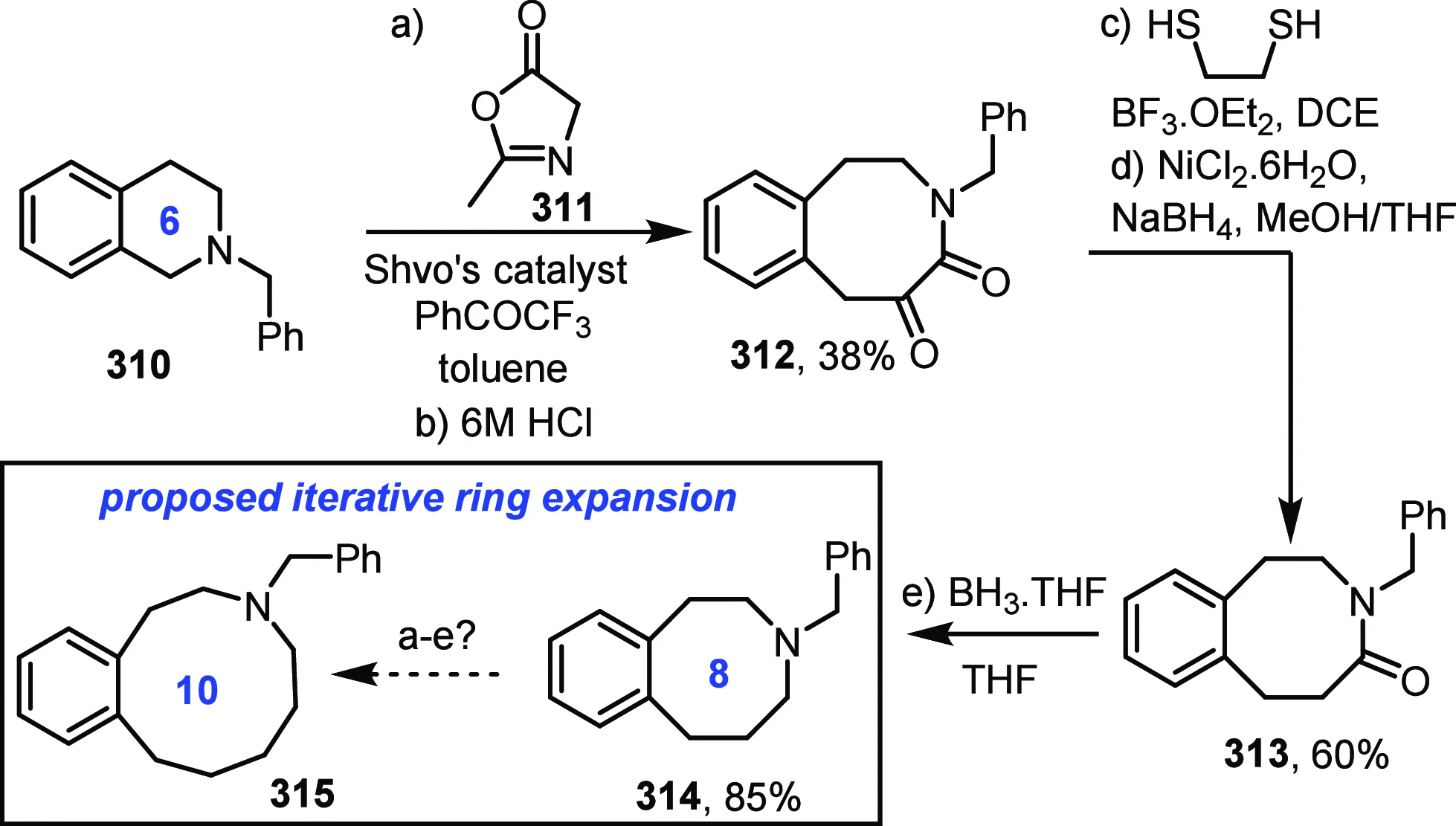
Ring Expansion of Saturated Cyclic Amines and a Proposed Iterative Ring Expansion Extension

Opportunities to apply established ring expansion consecutively will inevitably grow as new methods emerge and develop. Indeed, this is already happening, with successful examples of different ring expansions developed within the same lab being applied consecutively featuring in this review (e.g., Schemes 43, 47, and 48).

Combining compatible ring expansion approaches developed in different laboratories will expand the range of accessible products even further, and many potentially viable consecutive ring expansion sequences can be envisioned by combining methods featured in this review. For example, Back’s four-carbon ring expansion of cyclic amine **91** into nine-membered ring **316** could be followed by Chen’s two-atom cyclic amine homologation method to form 11-membered ring **317** (Scheme 50A). Similarly, six-membered-ring starting material **100** could potentially be converted into 19-membered macrocycles of the form **320** by applying the methods of Suh, Unsworth, and Rousseau in sequence (Scheme 50B). Combination with other approaches so far not used in consecutive ring expansion is also possible. For instance, Knowles’ two-carbon ring expansion could form viable substates for use in Unsworth’s SuRE methodology, allowing the double ring expansion of pyrrolidine **321** into 11-membered system **325**, which is itself functionalized to allow iterative ring expansion (Scheme 50C).^154^ The same combination of methodologies could also provide access to unusual polycyclic systems that remain largely unexplored by sequential ring expansion strategies, such as a ring expansion/cycloaddition sequence forming amino acid **323**, which could engage with SuRE to yield the 7,5,16-fused tricyclic system **326** (Scheme 50C).

**Scheme 50:**
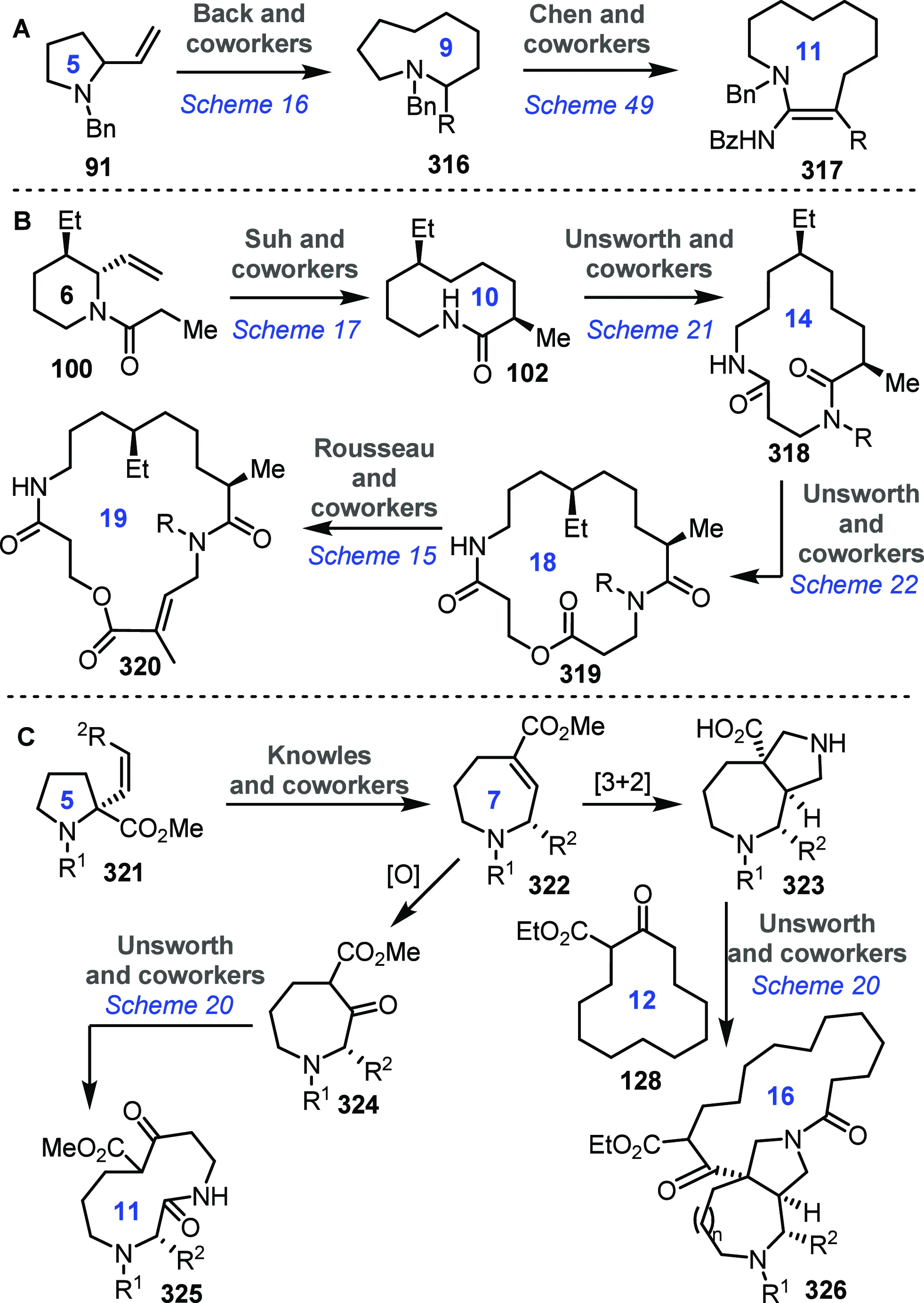
Potentially Viable Consecutive Ring Expansion Sequences Based on Established Methods

In summary, the ring expansion approaches described in this review clearly demonstrate the value of ring expansion reactions for the preparation of functionalized medium-sized rings and macrocycles. In the majority of cases, operation by a ring expansion enables the large ring products to be prepared at standard reaction concentrations and avoid impractical high dilution. This is one of the major drawbacks of classical end-to-end macrocyclization methods that can limit their utility, especially on scale.

Ring expansion methods hold a key place in the history of organic synthesis,^1–26^ and driven by ever-increasing interest in the synthesis of large-ring molecules for diverse applications,^33–50^ the future of the field looks bright. The strategies described in this review suggest that the scope of successive ring expansion will only continue to grow.

## Abbreviations

p-TSA: *p*-toluenesulfonic acid
KAPA: potassium 3-aminopropylamide
TMEDA: *N*,*N*,*N*′,*N*′-tetramethylethylenediamine
DMF: dimethylformamide
EvAS: *Emericella variecolor* astellifadiene synthase
PPi: inorganic pyrophosphate
T3P: propylphosphonic anhydride
DCM: dichloromethane
DMAP: 4-dimethylaminopyridine
EDC: 1-ethyl-3-(3-(dimethylamino)­propyl)­carbodiimide
HOBt: 1-hydroxybenzotriazole
TFA: trifluoroacetic acid
DBU: 1,8-diazabicycloundec-7-ene
LDA: lithium diisopropylamide
TMS: trimethylsilyl
Ts: tosyl
THF: tetrahydrofuran
LHMDS: lithium hexamethyldisilazide
MOM: methoxymethyl
pyr: pyridine
DCE: 1,2-dichloroethane
SuRE: successive ring expansion
Fmoc: 9-fluorenylmethoxycarbonyl
PG: protecting group
Cbz: benzyloxycarbonyl
Boc: *tert*-butoxycarbonyl
Fm: 9-fluorenylmethyl
Trt: trityl
Bn: benzyl
DMSO: dimethyl sulfoxide
CARE: conjugate addition/ring expansion
CCL5: C–C motif chemokine ligand 5
CCR5: C–C motif chemokine receptor 5
DMPU: 1,3-dimethyl-3,4,5,6-tetrahydro-2­(1*H*)-pyrimidinone
Ms: mesyl
AIBN: azobis­(isobutyronitrile)
TBAF: tetra-*n*-butylammonium fluoride
TBS: *tert*-butyldimethylsilyl
Phen: phenanthroline
DCB: 1,4-dicyanobenzene
DBH: 1,3-dibromo-5,5-dimethylhydantoin
IMes: 1,3-bis­(2,4,6-trimethylphenyl)­imidazol-2-ylidene
PMB: *p*-methoxybenzyl
PMP: *p*-methoxyphenyl
